# Photocatalysis Based on Metal Halide Perovskites for Organic Chemical Transformations

**DOI:** 10.3390/nano14010094

**Published:** 2023-12-28

**Authors:** Metikoti Jagadeeswararao, Raquel E. Galian, Julia Pérez-Prieto

**Affiliations:** Institute of Molecular Science, University of Valencia, C/Catedrático José Beltrán 2, 46980 Paterna, Valencia, Spain; jagadeeswararao.metikoti@uv.es

**Keywords:** photocatalysis, metal halide perovskite, heterostructures, organic chemical transformations

## Abstract

Heterogeneous photocatalysts incorporating metal halide perovskites (MHPs) have garnered significant attention due to their remarkable attributes: strong visible-light absorption, tuneable band energy levels, rapid charge transfer, and defect tolerance. Additionally, the promising optical and electronic properties of MHP nanocrystals can be harnessed for photocatalytic applications through controlled crystal structure engineering, involving composition tuning via metal ion and halide ion variations, dimensional tuning, and surface chemistry modifications. Combination of perovskites with other materials can improve the photoinduced charge separation and charge transfer, building heterostructures with different band alignments, such as type-II, Z-scheme, and Schottky heterojunctions, which can fine-tune redox potentials of the perovskite for photocatalytic organic reactions. This review delves into the activation of organic molecules through charge and energy transfer mechanisms. The review further investigates the impact of crystal engineering on photocatalytic activity, spanning a diverse array of organic transformations, such as C–X bond formation (X = C, N, and O), [2 + 2] and [4 + 2] cycloadditions, substrate isomerization, and asymmetric catalysis. This study provides insights to propel the advancement of metal halide perovskite-based photocatalysts, thereby fostering innovation in organic chemical transformations.

## 1. Introduction

**Figure 1:**
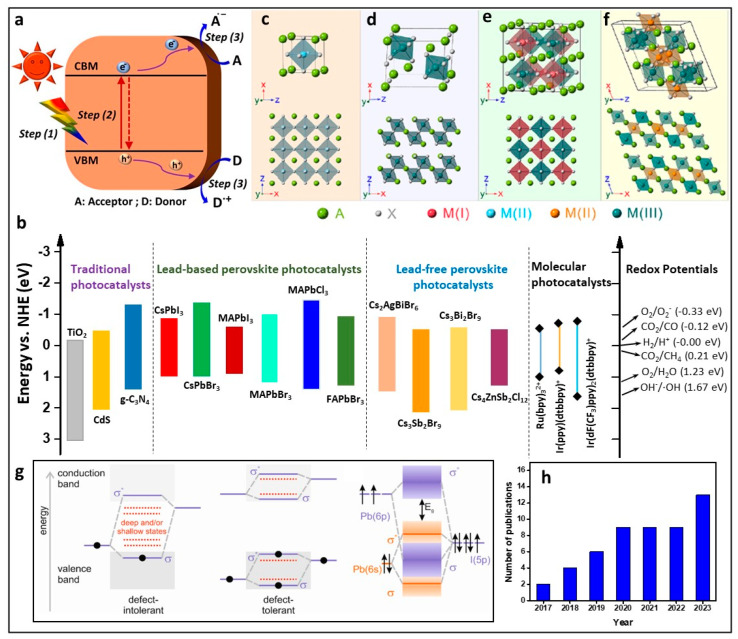
(**a**) Schematic demonstration of an organic reaction using perovskite NC under visible light illumination; (**b**) the energy land shape of various traditional semiconductors, lead-based and lead-free perovskites, and molecular photocatalysts; (**c**) AM^II^X_3_ 3D cubic perovskite; (**d**) A_3_M_2_^II^X_9_ 2D layered perovskite; (**e**) A_2_M^I^M_2_^III^X_6_ 3D cubic double perovskite and (**f**). A_4_M^II^M_2_^III^X_12_ layered double perovskite; top and bottom of the (**c**–**f**) show the unit cells of different perovskite crystal structures and the corresponding crystal structure models viewed from the [010] zone axis; (**g**) electronic band structure of conventional semiconductors (left side) and perovskites (middle and right side); (**h**) the number of publications per year from 2017 to 2023. (**c**–**f**) Reproduced with permission [12]. Copy right: 2020, American Chemical Society.

Solar energy, with its abundant availability, serves as the quintessential source of renewable power, offering a clean and accessible solution to the environmental challenges posed by fossil fuels and rising pollution levels [1]. Harnessing this energy through photocatalysis, a pivotal concept in the quest for global environmental sustainability, holds remarkable potential for effortlessly generating, storing, and releasing renewable energy on demand, contributing to a cleaner ecosystem and alternative energy solutions. Photocatalysis centres around specialized materials called photocatalysts, which generate electron–hole pairs (excitons) upon absorbing photons, thereby creating free radicals that participate in chemical reactions, as shown in Figure 1a, without the need for high temperatures or harsh conditions.

There are two types of photocatalysis: homogeneous and heterogeneous photocatalysis. The first one involves soluble photocatalysts, such as polypyridyl complexes of elements, such as ruthenium and iridium, as well as organic dyes like eosin Y and 9,10-dicyanoanthracene salts. These compounds smoothly interact with reactants (substrates) in the same phase, offering a wealth of opportunities for organic synthesis. The excited states induced by these photocatalysts initiate reactions that facilitate the formation of valuable carbon–carbon and carbon–heteroatom bonds, including chiral compounds via a stereo-selective process [2,3]. Moreover, photocatalysis has simplified the synthesis process by requiring a smaller amount of catalyst (1 mole% or less), using environmentally benign reagents, and operating under mild reaction conditions. Additionally, photocatalysis enables the formation of novel compounds that would otherwise be challenging or impossible to synthesize using conventional methods; for instance, C–H bond activation in saturated alkanes [3,4]. Nonetheless, the challenge lies in the intricate separation of homogeneous photocatalysts, which obstruct their reusability. Moreover, molecular photocatalysts are costly, thus imposing limitations on large-scale production in industries. Hence, there is a pressing need to innovate and advance novel heterogeneous photocatalysts that can demonstrate outstanding activity, on par with that of the existing noble metal-based catalysts, and provide high reaction yields under open-air conditions while addressing these shortcomings.

On the contrary, heterogeneous photocatalysis employs solid materials that exist in distinct physical phases from that of the substrates. This approach holds promise for directly functionalizing organic substrates, thereby engendering the creation or transformation of valuable organic molecules. The inherent advantages of solid photocatalysts, such as recyclability and easy separation from the medium, make heterogeneous photocatalysis an appealing avenue for sustainable synthesis initiatives. Among the catalysts used in this type of photocatalysis, solution-processable nanoparticles, nanorods, and nanosheets of materials such as TiO_2_, g-C_3_N_4_, metal sulphides, and perovskite oxides have undergone extensive scrutiny due to their high surface-to-volume ratio, high absorption cross-section, and suitable valence and conduction band energy levels, as shown in Figure 1b [5,6]. However, these materials exhibit absorption in the UV region and slight absorption in the visible, thereby rendering them unsuitable for visible light-driven photocatalytic applications. In search of alternatives, Cd-based II-VI semiconductor nanocrystals (NCs) have been explored for photocatalytic applications in the oxidation/reduction of organic compounds [7,8,9] due to their strong absorption in the visible light spectrum, high surface-to-volume ratio, and tuneable valence band maximum (VBM) and conduction band minimum (CBM) due to the quantum confinement effect (QCE). However, the presence of intra-mid gap states stemming from the non-passivated surface states in these Cd-based NCs, as shown in the left side of Figure 1g, leads to undesirable, non-radiative recombination of excitons, thus diminishing their activity [10]. Rectifying this issue requires extensive surface passivation to get rid of the intra-mid gap states; this involves complex synthesis procedures, such as the addition of an inorganic shell that improves their stability but reduces the charge transfer and, consequently, limits their application in photocatalysis [11].

On the other hand, perovskite materials with the AM^II^X_3_ stoichiometry, where A represents methylammonium (MA^+^), formamidinium (FA^+^), Cs^+^ (caesium); M^II^ represents Pb^2+^, Sn^2+^; and X represents Cl^−^, Br^−^, and I^−^, exhibit a unique three-dimensional (3D) crystal structure. In this structure, the M^II^-site cation is surrounded by six halide ions, forming octahedral units that share corners, thus resulting in a large void with cuboidal octahedral shapes. This void is occupied by the A-site cation. The crystal structure of perovskite (AM^II^X_3_) is depicted in Figure 1c. By changing the composition of AM^II^X_3_, the VBM and CBM can be adjusted, thus tuning the bandgap, as shown in Figure 1b. Furthermore, in stark contrast with conventional chalcogenide semiconductors, Pb-based perovskites exhibit a defect-tolerant electronic structure. This distinctive feature arises from the intricate interplay between a Pb lone pair s orbital and an iodine (halide) p orbital leading to anti-bonding coupling in the perovskite lattice. The outcome is the emergence of a bandgap between two antibonding bands, as illustrated on the right side of Figure 1g. Notably, this band structure directs structural defects, such as those originating from A-site cation vacancies and halide vacancies, to occupy energy levels within the VB and CB rather than lying within the bandgap [13]. The defect tolerance observed in lead halide perovskites is also, in part, ascribed to the ionicity of bonding [14]. Additionally, these materials exhibit outstanding characteristics, including excellent charge carrier transport [15,16], 3D connectivity, low excitonic binding energy, and simple solution processibility, making them ideal for various optoelectronic applications [15,17,18,19,20,21,22]. These materials have also shown promise in photocatalytic H_2_ generation [23], CO_2_ reduction [24], and organic transformations, including oxidation, C–C, C–N, C–O coupling reactions, and the formation of chiral organic compounds [25]. It is worth noting that perovskite-based photocatalysts also produced novel organic compounds, which would not have been possible by means of molecular photocatalysts [26]. This highlights the interest of the promising applications of MHP for photocatalytic organic transformations. However, concerns about the thermal and moisture stability of Pb-based perovskites have encouraged exploration of alternative materials.

Substituting Pb^2+^ with bi-valent metal ions, such as Sn^2+^ and Ge^2+^, is challenging due to their easy oxidation into Sn^4+^ and Ge^4+^, respectively. Incorporating metal ions with the ns^2^ electronic configuration, such as Bi^3+^ and Sb^3+^, results in an A_3_M_2_^III^X_9_ layered structure, as shown in the Figure 1d, in which the charge imbalance is addressed but at the expense of the disruption of the 3D perovskite structure.

To maintain both the structure and charge neutrality, A_2_M^I^M_2_^III^X_6_ double perovskites have been developed, as shown in the Figure 1e; they exhibit improved thermal stability but suffer from wide bandgaps and reduced photocatalytic performance. A recent breakthrough by Solis-Ibarra et al. introduced layered double perovskites (LDPs) composed of environmentally friendly elements exemplified by Cs_4_CuSb_2_Cl_12_ [27]. This innovative approach involves replacing M^I^ with M^II^ in 3D double perovskites, leading to the creation of LDPs with the stoichiometry A_4_M^II^M_2_^III^X_12_, thereby generating a new formula (e.g., A = Cs^+^ and Rb^+^; M^II^ = Ti^2+^, V^2+^, Cr^2+^, Mn^2+^, Fe^2+^, Co^2+^, Ni^2+^, Cu^2+^, Zn^2+^, and Cd^2+^; M^III^ = Sb^3+^ and Bi^3+^). These LDPs feature repeating layers of [M^III^Cl_6_]^3−^-[M^II^Cl_6_]^4−^-[M^III^Cl_6_]^3−^-vacancy, as shown in Figure 1f. Therefore, Cs_4_CuSb_2_Cl_12_, Rb_4_CuSb_2_Cl_12_, and Cs_4_ZnSb_2_Cl_12_ LDPs exhibit an optimal direct bandgap (1–1.8 eV) that holds promise for photocatalytic applications, coupled with impressive stability against humidity, light, and heat [28,29].

The existing body of literature has mainly focused on reviewing the photocatalytic activity of H_2_ generation and CO_2_ reduction as well as dye degradation. Comparatively, less attention has been devoted to exploring organic transformations [23,30,31,32,33,34,35]. In addressing this gap, this review aims to focus on recent developments, underscoring a growing interest that is indicated by the rising number of publications on perovskite-based organic chemical transformations from 2017 onwards, as illustrated in Figure 1h. Specifically, the review delves into organic transformations utilizing both MHP bulk materials and NCs. Additionally, it extends its scope to heteronanocrystals resulting from combinations with other structures, such as TiO_2_, g-C_3_N_4_, CdS, Mxene, among others. By scrutinizing these particular areas, the review endeavours to offer valuable insights into the application of perovskite materials in catalysing organic transformations. The temporal focus from 2017 to 2023 enables an exploration of recent advancements, contributing to the fundamental understanding and unlocking the potential of perovskite-based photocatalysis in the realm of organic transformations.

Bulk perovskite materials have a smaller surface area than perovskite NCs (PNCs), and consequently, they exhibit lower photocatalytic activity, as demonstrated by Wu et al. For example, the yield of disulphide product formation in a thiophenol coupling reaction was <8% when using CsPbBr_3_ bulk powder, but it increased up to 98% when using the NCs [36]. The high surface-to-volume ratio of the NCs provides a large active surface area, thus increasing the chances of an interaction between the photocatalyst and reactants. Moreover, PNC composition and size, and therefore the conduction and valence band edges of perovskite NCs, can be readily adjusted [37], thereby enabling favourable redox processes. In addition, the capping of PNCs with organic ligand(s) allow for their dispersion in organic solvents, thus expanding their potential applications. Furthermore, one notable advantage of PNCs is their ease of separation from organic mixtures by centrifugation, which facilitates the recovery and purification of the PNCs. Notably, perovskite NCs can be easily synthesized at room temperature under ambient conditions [38,39] using simple synthetic procedures, such as ligand-assisted re-precipitation (LARP) and emulsion methods, to yield a large number of NCs and different morphologies.

## 2. Factors Effecting the Photocatalytic Performance

In the context of organic synthesis, the manipulation of the reaction condition is pivotal for achieving the desired product with an optimal yield. To unlock the full potential of PNCs for their photocatalytic activity, it is imperative to comprehensively assess various aspects, including the impact of PNC size and bandgap tuning on its photocatalytic performance, interaction with solvents, and considerations related to acidity, ions, and moisture.

### 2.1. Effect of Size of NCs on Photocatalytic Activity

The use of semiconductors as photocatalysts requires them to exhibit a high absorption coefficient and ensure the photogenerated charge carriers reach the surface promptly with a minimum recombination. In addition, a suitable band alignment is crucial to enable the efficient charge transfer for oxidation and reduction processes. The use of small NCs is pivotal to achieving these objectives; their diminutive size facilities the swift transport of the photogenerated charge carriers to the surface. Semiconductor NCs exhibiting QCE are particularly promising candidates for photocatalysis.

#### 2.1.1. Size Dependent Photocatalytic Activity of Lead Halide PNCs

**Figure 2:**
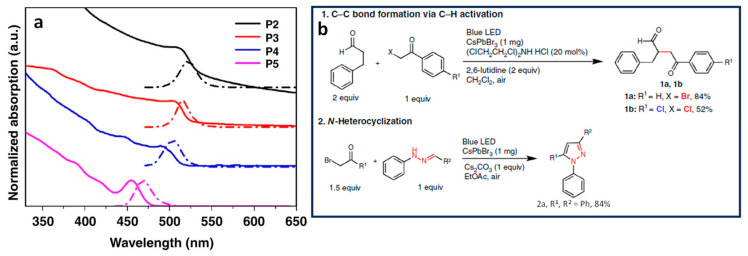
(**a**) UV–visible absorption and PL spectra (dashed lines) of different-sized CsPbBr_3_ NCs, P2-P5; (**b**) formation of Csp^3^-Csp^3^ and Csp^3^-Nsp^3^ bonds shown in red colour and their respective yields in reaction 1 and 2.

It is widely recognized that strong QCE occurs when the NC size is smaller than the Bohr radius of the material. The Bulk Bohr exciton radius has been calculated using the equation $a_{b}=\varepsilon_{r}\left(m_{0}/µ\right)a_{0}$, where $a_{b}$ is the exciton Bohr radius, $\varepsilon_{r}$ is the relative dielectric constant of the bulk semiconductor, $m_{0}$ is the mass of a free electron, µ is the reduced mass of the exciton, and $a_{0}$ is the Bohr exciton radius of the hydrogen atom (0.529 Å). The calculated Bohr radius for CsPbBr_3_ is 3.5 nm. The calculated Bohr exciton radius for CsPbBr_3_ is 3.5 nm [37,40]. Figure 2 shows the dependence of the UV–visible and photoluminescence (PL) spectra of different-sized CsPbBr_3_ NCs intended for Csp^3^-Csp^3^ and Csp^3^-Nsp^3^ bond formation and their respective yields.

CsPbBr_3_ NCs, under quantum confinement, exhibit size-dependent properties. Zhu et al. conducted a study on the catalytic performance of organic compounds using different sizes of CsPbBr_3_ NCs. They prepared octylamine-capped NCs with different edge lengths, which exhibited bulk-like NCs from 2 to 100 nm (P1) and quantum-confined NCs, such as oleylamine-capped NCs with sizes of 14, 9, 6, and 4 nm (P2, P3, P4, and P5, respectively). Figure 2a shows the size-dependent absorption and PL of the different-sized CsPbBr_3_ NCs and their use as photocatalysts for Csp^3^-Csp^3^ bond formation via C–H bond activation (see reaction 1 and 2 in Figure 1b).

The results showed that smaller PNCs exhibited a higher initial activity for C–H bond activation, leading to the formation of product 1a in Figure 1b. Interestingly, P2–P4 NCs achieved product yields of 54–64% within 40 min, but the prolonged reaction time did not increase the yield significantly. On the other hand, P1 initially showed a slower production of 1a in Figure 1b but improved the yield up to 85% with a prolonged reaction time. This disparity was attributed to the small NCs, whose higher surface area-to-volume ratio enabled a swifter reaction rate during the initial stages. Moreover, it was observed that moisture in the solvent interacted more substantially with the smaller NCs as opposed to the larger ones, this resulting in progressive perovskite degradation and, hence, activity decrease. In addition, P5 exhibited a yield of 8%, primarily owing to its limited absorption of visible light and its increased susceptibility to degradation. In contrast, PNCs were stable when the reactions were performed under a pre-dried, non-halide solvent as ethyl acetate. Product 2a in Figure 2b was produced in an 87% and 86% yield with P1 and P2 after 6 and 2 h of reaction, respectively. This underscores the pivotal role of smaller-sized NCs in achieving high yields in shorter time frames.

#### 2.1.2. Size Dependent Photocatalytic Activity of Lead-Free Halide PNCs

**Figure 3:**
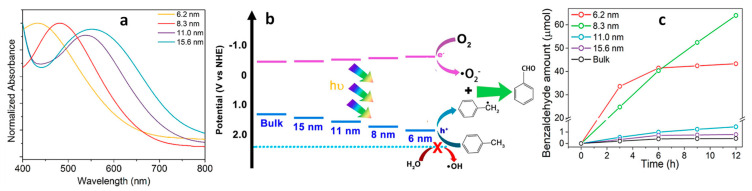
(**a**) UV–vis absorption spectrum of various-sized Cs_4_ZnSb_2_Cl_12_ layered double perovskite (LDP) NCs; (**b**) scheme of the energy levels of the conduction and valence band edges for bulk and different-sized Cs_4_ZnSb_2_Cl_12_ NCs and mechanism of photocatalytic toluene oxidation to benzaldehyde formation; (**c**) time-dependent photo catalytic activity of benzaldehyde formation using bulk and NCs of Cs_4_ZnSb_2_Cl_12_. Reproduced with permission. Copyright: 2023, American Chemical Society.

To the best of our knowledge, the systematic study of quantum-confined, size-dependent photochemical activity has not yet been explored using A_3_M_2_^III^X_9_ and A_2_M^I^M_2_^III^X_6_ type NCs [41]. Moreover, A_4_M^II^M_2_^III^X_12_ type NCs, specifically Cs_4_ZnSb_2_Cl_12_ LDP NCs, showed size-dependent optical properties. The calculated Bohr diameter of Cs_4_ZnSb_2_Cl_12_ was 5.5 nm or above (due to µ changes with different crystal facets). Caruso et al. reported different-sized organic capped Cs_4_ZnSb_2_Cl_12_ NCs, namely 6.2 nm, 8.3 nm, 11.0 nm, and 15.6 nm NCs in the form of spherical dots [29]. Figure 3a displays the size-dependent absorption features of Cs_4_ZnSb_2_Cl_12_ NCs and, consequently, the bandgap tuning with the QCE. The authors conducted calculations to determine the CBM and VBM for bulk and various-sized Cs_4_ZnSb_2_Cl_12_ NCs, as presented in Figure 3b. Importantly, QCE was observed to fine-tune the edges of the conduction and valence bands. Notably, the shift in the VBM proved to be more significant than that of the CBM, thus providing an appropriate band alignment conducive to driving the oxidation of toluene. As shown in Figure 3c, smaller-sized NCs exhibited a higher photocatalytic performance than bulk Cs_4_ZnSb_2_Cl_12_. Specifically, the NCs with sizes of 6.2 nm and 8.3 nm exhibited superior performance when compared to larger-sized NCs. Notably, the 6.2 nm NCs displayed the highest activity during the initial 6 h, but their activity declined over time when compared to that of the 8.3 nm NCs. This reduction in activity can be attributed to the gradual degradation of the 6.2 nm NCs when exposed to water released during the prolonged benzaldehyde formation process.

In general, both Pb and Pb-free PNC-based photocatalytic reactions have shown that smaller-sized NCs with suitable light absorption characteristics exhibit high photocatalytic activity. However, this increased activity often increases at the cost of compromising their stability over extended periods. Therefore, it is vital to enhance the stability of smaller NCs for performing chemical reactions under ambient/water conditions. One potential approach involves the application of a thin SiO_2_ layer to shield the perovskite NCs, as suggested in previous studies [42,43]. Another strategy entails the incorporation of these smaller PNCs into the pores of stable materials, such as melamine foam, to address the stability concerns [44]. Further investigation is warranted to comprehensively assess the photocatalytic performance and stability of these composite materials across a range of chemical reactions.

### 2.2. Stability and Reaction Condition Tolerance

In the context of organic synthesis using photocatalysis, the evaluation of perovskite materials becomes crucial, considering factors such as the co-catalyst, atmosphere, and type of solvent.

#### 2.2.1. Role of a Co-Catalyst

CsPbBr_3_ NCs have been used in different reaction mediums to investigate the impact of ion effects and the role of solvents in perovskite photocatalysis. Reaction 1a in Figure 2b involves the use of (ClCH_2_CH_2_Cl)_2_NH_2_Cl as a co-catalyst with CsPbBr_3_ NCs photocatalyst, resulting in the formation of CsPbBr_x_Cl_3−x_ NCs. However, it is worth noting that the co-formation of excess Br ion during reaction 1a in Figure 2b could potentially lead to the subsequent Cl ion exchange with CsPbBr_x_Cl_3−x_, thus resulting in the formation of the more stable CsPbBr_3_ perovskite. Conversely, reaction 1b in Figure 2b, which used Cl-substrates, demonstrated the complete transformation of CsPbBr_3_ NCs to CsPbCl_3_ NCs after the reaction. Furthermore, when non-halide solvents were employed in reaction 2a in Figure 2b, the overall stability of the PNCs (P1 and P4) was enhanced, thus improving the perovskite recyclability.

#### 2.2.2. Role of Acidity

**Figure 4:**
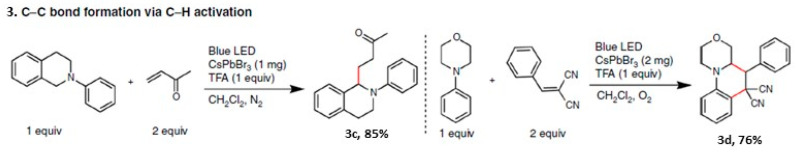
C–C bond product formation (shown in red colour bonds in the products 3c and 3d) and respective chemical yields.

The role of acidity in perovskite photocatalysis is significant. When various organic acids, such as propionic acid, benzoic acid, or trifluoroacetic acid (TFA), are used as co-catalysts or substrates, an enhancement in PL was observed for P1 CsPbBr_3_ NCs up to an optimum concentration. This enhancement has been attributed to the carboxylic acid acting as a capping ligand through strong hydrogen bonding with the halide ions and to a robust interaction between the carboxylic acid and Pb atoms. Interestingly, the optimized concentration of TFA, which exhibits high PL intensity, also led to maximum product yields for specific reactions 3c and 3d in Figure 4. Therefore, non-halide organic acids not only stabilize the PNCs but also enhance the overall catalytic efficiency.

#### 2.2.3. Role of Air

Air tolerance is an important aspect in chemical synthesis. While molecular photocatalysts typically require air-free conditions, PNCs do not. In fact, PNCs exhibit strong quenching with organic substrates when compared to oxygen (O_2_). In contrast, molecular photocatalysts often show significant and competitive O_2_ quenching, thus resulting in a poor catalytic performance. Consequently, PNCs demonstrate catalytic performance one to two orders of magnitude higher for reactions 1, 2, and 3 (in Figure 2b and Figure 4) than that of CdSe and molecular photocatalysts, making them highly efficient in air-tolerant reactions.

### 2.3. Role of Bandgap Tuning of Perovskite NCs on Photocatalytic Organic Reactions

**Figure 5:**
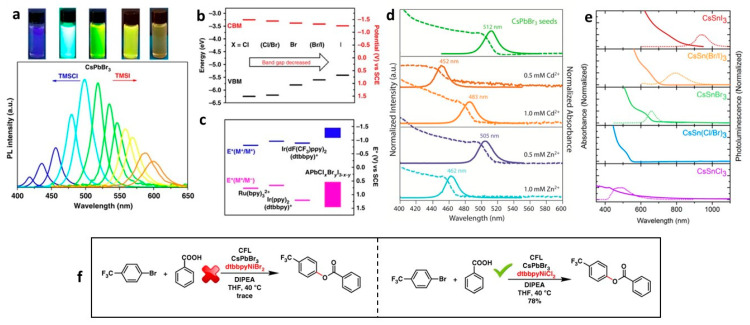
(**a**) Tunability of photoluminescence (PL) spectra of CsPbBr_3_ NCs using anion exchange with trimethylsilyl chloride or iodide and their photographs under UV illumination; (**b**) band edges of APbCl_x_Br_y_I_3−x−y_; (**c**) excited state potential (E*) range of APbCl_x_Br_y_I_3−x−y_ comparing with noble-transition metal photocatalysts; (**d**) absorption (dashed lines) and PL spectra of Zn- and Cd-doped CsPbBr_3_ NCs. Reproduced with permission [45]. Copyright: 2017, American Chemical Society; (**e**) absorption and PL spectra (dashed lines) of CsSnX_3_ NCs. Reproduced with permission [26]. Copyright: 2016, American Chemical Society; (**f**) C–O bond formation with perovskite band-tuning.

Bandgap tuning plays a crucial role in selectively activating organic substrates during photocatalytic reactions. Three strategies can be used to tune the bandgap of metal halide PNCs (AM^II^X_3_), specifically engineering the A- and M^II^-site cations and X-site anion. Out of these three strategies, halide exchange has been promising for tuning the bandgap [14]. Figure 5a shows the colour and PL tuning across the entire visible range of lead halide PNCs by treating the CsPbBr_3_ NCs with chloride and iodide precursors. Figure 5b shows the band alignment of CsPbX_3_ NCs (X: Cl, Br, and I). When the X changes from Cl to I from Br, different energy levels can be accessed, such as the well-known molecular catalysts. This allows for the activation of some interesting photochemical reactions, specifically for C–O bond formation. When CsPbBr_3_ NCs were used in combination with dtbbpyNiBr_2_ as a co-catalyst, only a minimal amount of product was generated, as shown in the reaction in Figure 5f. In contrast, when dtbbpyNiCl_2_ was present alongside CsPbBr_3_, a 78% yield was achieved. This indicates that the anion exchange process, wherein Br is replaced by Cl, shifts the PNC CBM to a higher energy, as shown in Figure 5b. This shift eases energetically favourable electron transfer processes during the reaction. Substantiating this mechanism, the study observed efficient triplet energy transfer from Ir(bpy)_3_ molecular photocatalysts to the substrates, a result achieved through the modification of substituent groups.

B-site engineering of lead halide PNCs (LHPNCs) leads to the tuning of the bandgap in a relatively lower range than halide exchange. For instance, in CsPbBr_3_ NCs, a partial substitution of Pb^2+^ with a smaller transition metal ions, such as Zn^2+^ and Cd^2+^, would increase the bandgap by ~40–60 nm, as shown in the Figure 5d, by contracting the lattice [45]. On the other hand, the introduction of lanthanide metal ions with a +3 oxidation state (e.g., Ce^3+^, Eu^3+^, Sm^3+^, Tb^3+^, Dy^3+^, Er^3+^, and Yb^3+^) [46,47,48] and a transitional Mn^2+^ ion [48,49] leads to multicolour emission peaks related to dopant and hosts emission peaks spanning from visible to the near infra-red (NIR) region by creating new luminescent centres but with slightly less or no changes in the bandgap of the host NCs [47]. In contrast, the complete replacement of Pb^2+^ with Sn^2+^ (CsSnX_3_) allow for the bandgap tuning of lead-free PNCs in the visible and NIR region, as shown in Figure 5e. However, the photocatalytic performance of these doped and CsSnX_3_ NCs for organic transformations are yet to be explored [50]. Furthermore, the halide exchange strategy can also be extended to other lead-free perovskites NCs A_3_M_2_^III^X_9_ and A_2_M^I^M^III^X_6_ to tune the bandgap [51,52].

The influence of the A-site cation on the bandgap in MHP is generally minimal, as the A-site cation does not directly participate in shaping the electronic band structure. However, slight adjustments in the bandgap can be achieved by considering the size differences of the A-site cation, which may lead to alterations in the octahedral tilting and/or lattice contraction or expansion [53,54]. Hazarika et al. demonstrated that introducing Cs into FAPbI_3_ NCs resulted in the fine-tuning of absorption and emission properties, shifting them from 800 nm to 650 nm [55].

## 3. Types of Heterojunctions for Photocatalysis

The superior photocatalytic performance of PNCs compared to bulk perovskite is evident. Nevertheless, the heightened Coulombic attraction force between opposite charge carriers within smaller NCs often results in a pronounced recombination, thereby hampering the migration of charge carriers. To ameliorate this issue and enhance the separation of electron–hole pairs, leading to an improved photocatalytic performance and increased stability of PNCs, diverse heterostructures have been innovatively engineered. These heterostructures incorporate varying band alignments, encompassing Schottky, type-II, and Z-scheme heterojunctions, thereby opening new avenues for advanced materials and enhanced photocatalysis. These heterostructures have been prepared by combining semiconductor–conductor and semiconductor (SC I)–semiconductor (SC II) with unequal band energy levels.

**Figure 6:**
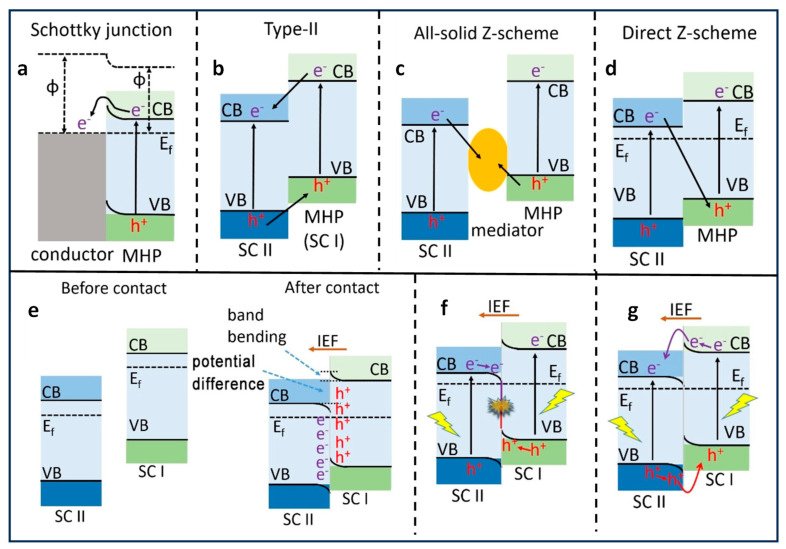
Schematic illustration of the band alignments of several possible heterojunctions. (**a**) Schottky heterojunction; (**b**) type-II heterojunction; (**c**) all-solid-z-scheme hetero junction; (**d**). direct Z-scheme heterojunction; (**e**) the relative band positions and Fermi level of semiconductor (SC) I and SC II before and after contact; (**f**,**g**) After illumination with light, the charge migration follows the Z-scheme and type-II pathways. VB: valence band; CB: conduction band; E_f_: Fermi level (dashed line); IEF: internal electric field. Reproduced with permission [58]. Copyright: John Wiley and Sons.

Various metals, such as Au, Ag, Pt, and carbon-based materials, such as graphene and reduced graphene, possess Fermi energy levels that are relatively positive in comparison to perovskite materials. When these conductive materials are in contact with the perovskite materials, electrons are transferred from the semiconductor to the metal until equilibrium is reached. This electron transfer leads to an upward shift in the energy bands of the semiconductor at the interface, thus resulting in band bending, as illustrated in Figure 6a. This type of junction is commonly referred to as a Schottky junction, and the band bending it creates gives rise to an energy barrier known as the Schottky barrier. The magnitude of the band bending and the Schottky barrier depends on the difference in work function between the metal and semiconductor [56]. It is important to note that the effect of band bending is less pronounced when dealing with semiconductor NCs in contrast to bulk semiconductors. Consequently, establishing Schottky junctions with NCs is considered a promising approach to enhance photocatalysis. Schottky heterojunctions serve a dual purpose in this context; they not only facilitate the extraction of charge carriers by promoting charge separation, but they also act as a co-catalyst, offering active catalytic sites essential for specific photocatalytic processes [57].

When two semiconductors SC I and SC II, each having differing CB and VB energies, come into contact, they can form a type-II heterojunction, as depicted in Figure 6b. This unique type-II band alignment enables the transfer of photogenerated electrons from SC I to SC II and photogenerated holes from SC II to SC I. Recent studies have extensively explored type-II band alignment through the creation of heterojunctions involving perovskites and various semiconductors, such as TiO_2_, graphitic carbon nitride (g-C_3_N_4_), CdS, etc. Depending on the specific characteristics of the semiconductors involved, some of these heterojunctions may even result in the formation of p-n heterojunctions. When p-type and n-type semiconductors with differing Fermi energy levels combine to form a junction, the flat bands of n-type and p-type undergo upward and downward band bending, respectively, at the interface. This thermodynamically favourable band alignment eases the separation of photogenerated electrons and holes, causing them to reside on different sides in distinct semiconductors, thus enhancing the electron and hole separation and thereby increasing photocatalytic performance.

To expedite the separation and transfer of photogenerated electrons and holes, a commonly employed approach is the utilization of a type-II band alignment. However, in type-II heterojunctions, the redox potential of the photocatalyst is often compromised due to oxidation and reduction processes occurring on the semiconductor with lower oxidation and reduction potentials, respectively [59]. In contrast, a Z-scheme heterojunction can maximize the redox potential of the heterostructures while maintaining efficient light absorption [60]. This proves particularly advantageous in cases where high reduction potential CB and oxidation potential VB are required, such as in a water splitting reaction. The band alignment in Z-scheme heterojunctions closely resembles that of type-II heterojunctions, with a key distinction being the direction of photogenerated electron transfer from SC II to SC I as illustrated in Figure 6c, d. Initially, all-solid Z-scheme heterojunctions where two different semiconductors were connected via an electron mediator, such as Ag, Au, and Pt, were reported. Under illumination, photoinduced electrons are transferred from the CB of SC II to VB of SC I through the electron mediator (Figure 6c). Consequently, the photogenerated holes and electrons in SC II and SC I are spatially separated and can be harnessed for various photocatalytic applications. However, the use of such expensive materials as electron mediators restricts their large-scale application in photocatalysis. Subsequently, direct Z-scheme heterojunctions were developed, where two different semiconductors with varying Fermi levels were in direct contact with each other. This results in charge distribution at the interface under dark conditions, causing band bending and the creation of an interfacial built-in electric field (IEF) in direct Z-scheme heterojunctions (Figure 6d). Moreover, a substantial difference in work function between SC I and SC II results in the formation of relatively large barriers that hinder electron transfer from SC I to SC II and hole transfer from SC II to SC I, defining the formation of a direct Z-scheme heterojunction. However, when the difference of work functions of SC I and SC II is smaller, a type-II band alignment can still co-exist with a Z-scheme heterojunction. Researchers have demonstrated the formation of both type-II and Z-scheme heterojunctions using the same perovskite materials. For instance, Tüysüz et al. [61] prepared a type-II heterojunction with CsPbBr_3_/TiO_2_, while Yu et al. established a Z-scheme heterojunction [62]. It appears that two different semiconductors with n-type characteristics can form a Z-scheme heterojunction. 

The photocatalytic activity of MHP and their heterostructures for the most representative organic transformation are summarized in Table 1 and discussed in detail in Section 4, Section 5, Section 6 and Section 7.

## 4. Carbon–Carbon (C–C) Bond Formation

C–C bond formation is a foundational process in organic synthesis, crucial for creating complex molecular structures [63]. Traditional methods, including artificial photoredox catalysis, have advanced, but they often require noble metals or complex procedures [3,64]. Addressing this, NCs emerge as promising photocatalysts for C–C bond formation under visible light. Unlike conventional systems, PNCs offer simplified product separation, eliminating the need for costly noble metal catalysts. Their unique advantage lies in an efficient energy transfer to organic substrates, initiating photocatalytic reactions with long-lived molecular triplets. This introduces a tuneable and versatile photocatalyst, showcasing PNCs as a potential game-changer for sustainable and efficient C–C bond-forming reactions under mild conditions. In the following subsections, we delve into recent advancements in C–C coupling reactions utilizing PNCs as photocatalysts.

### 4.1. α-Alkylation of Aldehydes Using LHPNCs

**Figure 7:**
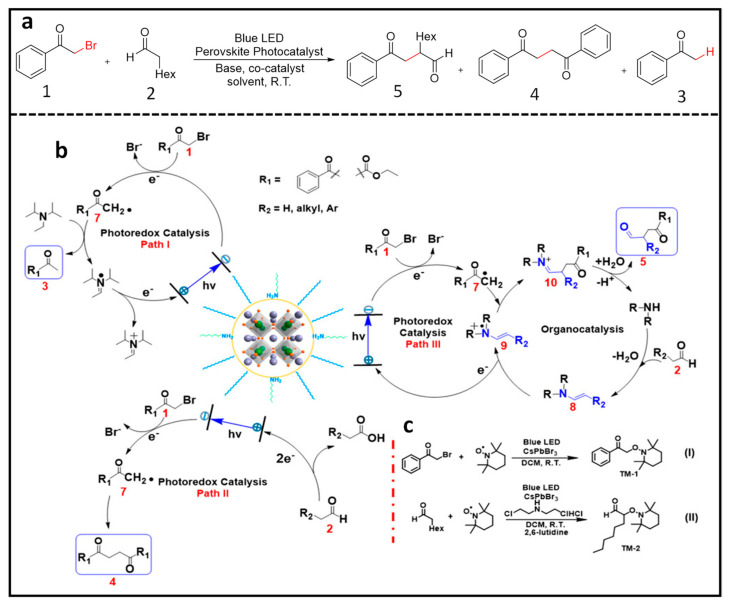
(**a**) Photocatalytic reductive dehalogenation, sp^3^-C coupling, and α-alkylation of aldehydes; (**b**) proposed mechanism for perovskite catalyzed dehalogenation, sp^3^ carbon coupling, and α-alkylation; (**c**) TEMPO trapped experiment for radical intermediate validation. Reproduced with permission [65]. Copyright: 2019, American Chemical Society.

Zhu et al. demonstrated the notable selectivity and high yield achieved in the α-alkylation of aldehydes. This process facilitated the formation of C–C bonds and reduction of C–Br using CsPbBr_3_ and MAPbBr_3_ nanoparticles (NPs) as photocatalysts under blue LED irradiation [65]. Impressively, they reported an exceptionally high turnover number (TON) exceeding 52,000, surpassing the efficiency of catalysts based on noble metals. In this method, octylamine-capped CsPbBr_3_ NPs (P1) were used. Under blue LED illumination in an open-air environment and in the presence of CsPbBr_3_ NPs, dicyclohexylamine as a co-catalyst, and 2,6-lutidine as a base, compounds **1** and **2** (Figure 7a) yielded compounds **3**, **4**, and **5** (Figure 7a). The formation of these three compounds was explained by a radical mediated mechanism involving three pathways. Upon perovskite photoexcitation, the electron is transferred from compound **1** and is reduced, and it eventually forms radical 7 (Figure 7b). In the presence of a sacrificial donor, N,N-diisopropylethylamine (DIPEA), radical 7 transforms into compound **3** (path I in Figure 7b). In the absence of a donor, radical 7 self-couples, resulting in the formation of compound **4** (path II in Figure 7b). The regeneration of the photocatalyst occurs through the oxidation of the electron donor and aldehyde in path I and path II, respectively. In path III (Figure 7b), oxidative quenching with in-situ generated enamine 8 (formed by the reaction between dicyclohexylamine and octanal with a loss of a water molecule) leads to radical cation 9, which, upon reaction with radical 7 followed by hydrolysis, generates the iminium cation 10, which releases product 5 (Path III in Figure 7b) and regenerates the co-catalyst. Additionally, radical trapping experiments were performed to confirm the formation of radical intermediates using TEMPO, as shown in Figure 7c.

**Figure 8:**
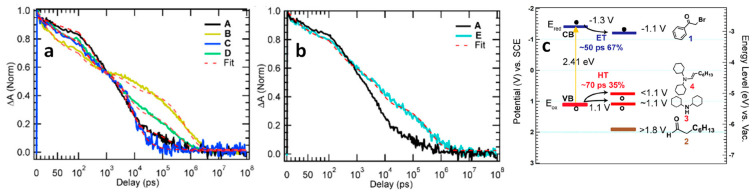
(**a**) Normalized transient absorption (TA) kinetics probed at the centre of the NCs exciton bleach spectrum for A–D, red-dashed traces are fits to kinetics; (**b**) normalized TA kinetics and fits for A and E where A, B, C, D, E represent pure CsPbBr_3_ NCs, CsPbBr_3_ NCs + 2-bromoacetophenone, CsPbBr_3_ NCs + octanal, CsPbBr_3_ NCs + octanal + dicyclohexylamine, CsPbBr_3_ NCs + dicyclohexylamine, respectively; (**c**) electrochemical potential and energy level for reactants and CsPbBr_3_ NCs. Reproduced with permission. Copyright: 2020, American Chemical Society.

To gain a deeper understanding of the reaction mechanism, Wang et al. employed transient absorption (TA) spectroscopy to investigate the charge transfer and reaction dynamics, focusing specifically on the selective formation of product 5 (Figure 7a) [66]. In this study, amine-free CsPbBr_3_ NCs were utilized as photocatalysts to avoid a potential charge transfer to the amine ligand. Figure 8a shows the results of the ground-state bleach dynamics for different systems: pure CsPbBr_3_NCs (A), CsPbBr_3_ NCs + 2-bromoacetophenone (B), CsPbBr_3_ NCs + octanal (C), CsPbBr_3_ NCs + octanal + dicyclohexylamine (D), and CsPbBr_3_ NCs + dicyclohexylamine (E). The analysis revealed that both B, D, and E (Figure 8a,b) exhibited decay times of ~50 ps, ~70 ps, and ~70 ps, respectively, followed by a slower decay pattern compared to pure CsPbBr_3_ NCs (A). Figure 8c provided insights into the reduction and oxidation potentials of the reactants, photocatalysts, and co-catalysts. The energy level of 2-bromoacetophenone was favourable for ultra-fast electron transfer, occurring within ~50 ps. On the other hand, dicyclohexylamine and the in situ-formed enamine facilitated the hole transfer, with a timescale of ~70 ps. However, it was determined that a hole transfer to dicyclohexylamine did not result in the desired oxidized enamine intermediate, eliminating this possibility from the mechanism. Consequently, following the ultrafast electron and hole transfer processes, charge-separated states were formed with a lifetime of approximately 0.8 µs. This 0.8 µs charge-separated state allows the photogenerated-charged radical intermediates to undergo C–C bond formation through a biradical pathway [66].

### 4.2. Benzyl Bromide Coupling Using LHP NCs

**Figure 9:**
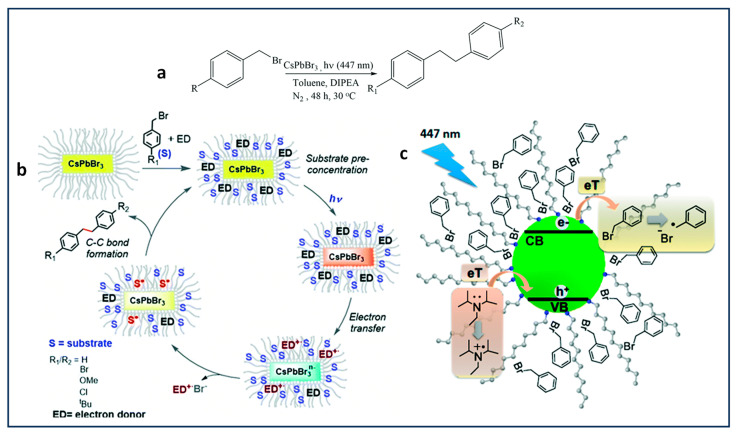
(**a**) Photocatalytic aryl bromide coupling reaction; (**b**) schematic representation of the cooperative action between the NC surface and the capping for the catalysed coupling reaction; (**c**) the process occurring after excitation of the CsPbBr_3_ NCs by vis-lamp in the presence of benzyl halide as electron acceptor and DIPEA as electron donor within the capping.

Pérez-Prieto and co-workers have demonstrated that the use of dodecylamine-capped CsPbBr_3_ NCs, combined with an electron donor, eventually results in photoredox-catalysed homo- and cross-coupling of benzyl bromides under visible light (447 nm) excitation, as shown in Figure 9a [67]. In this study, the authors found the thermodynamically unfavourable band alignment for electron transfer between benzyl bromide (−1.5 V vs. SHE) and CBM of CsPbBr_3_ NCs (−0.96 V vs. SHE). This, in principle, unexpected result was rationalized as due to substrate pre-concentration at the NC surface, facilitated by the van der Walls interaction between the benzyl bromide and alkyl chains of the organic ligand anchored to the NC surface, followed by an electron transfer from the NC to the benzyl bromide, thus eventually generating a high concentration of benzyl radicals close to or at the NC surface and driving the C–C coupling reaction forward.

Moreover, the hole in the valence band of CsPbBr_3_ refilled by an electron transfer from DIPEA facilitated by the favourable HOMO level (−5.76 V) relative to the VBM of CsPbBr_3_ (between −5.85 to −6.4 V), as shown in the Figure 9c. Notably, a TON of 17,500 with a >80% product yield was achieved for the C–C coupling of benzyl bromide. Additionally, the authors discovered that the product yield is influenced by substitution at the para position, with the yield being H, tBu, OMe > Cl > Br.

### 4.3. Stereo Selective C–C Oxidative Coupling Reaction

**Figure 10:**
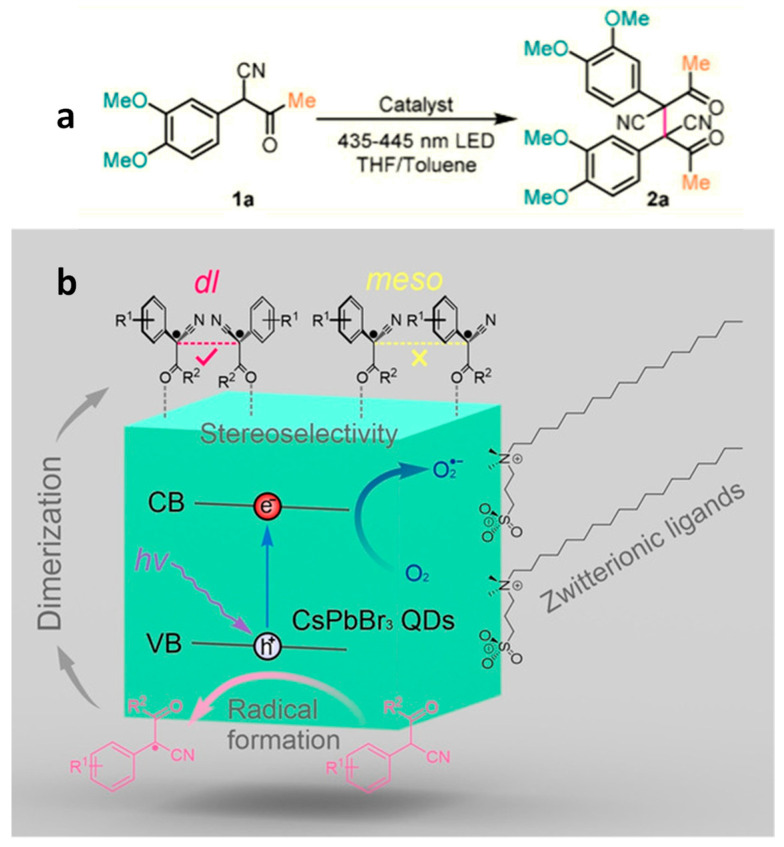
(**a**) Photocatalytic stereo-selective C–C oxidative coupling reaction of 2-(3,4-dimethoxyphenyl)-3-oxobutanenitrile; (**b**) proposed reaction scheme for the stereoselective dimerization of α-keto nitriles photocatalyzed by zwitterion capped CsPbBr_3_ NCs. Reproduced with permission. Copyright: 2020, John Wiley and Sons.

Chen and co-workers demonstrated the first photocatalytic stereoselective C–C oxidative coupling reaction using PNCs [68]. They used 2-(3,4-dimethoxyphenyl)-3-oxobutanenitrile (compound **1a** in Figure 10a) as a model substrate and CsPbX_3_ (X = Br, I) NCs as photocatalyst under visible light illumination. This study revealed the critical role of surface ligands ensuring a stable reaction. In this context, CsPbX_3_ NCs capped with oleic acid/oleylamine (OA/OAm) exhibited impressive results, with a yield of 95% and excellent stereoselectivity of 99%. However, a significant drawback was the rapid degradation of OA/OAm-capped CsPbX_3_ NCs during the photocatalytic reaction. This issue was attributed to the instability of CsPbI_3_ NCs and possible ligand detachment during the post-reaction purification process. To address this challenge, the authors introduced surface modification to transform OA/OAm-CsPbBr_3_ NCs into zwitterionic (ZW) ligand-capped CsPbBr_3_ NCs. This innovation not only improved the recyclability of the reaction, extending its usability to at least three cycles, but also enhanced the reaction kinetics. This enhanced reactivity was explained by the reduction in the density of surface ligand coverage, decreasing from 5.4 nm^2^ for OA/OAm-capped CsPbBr_3_ NCs to 3.0/nm^2^ for ZW- capped CsPbBr_3_ NCs. This reduction facilitated increased substrate adsorption onto the NC surface and lowered the kinetic barrier for the reaction. It is worth noting that ZW ligands strongly adhered to the PNC surface, which enabled multiple washing steps during the NC purification process to remove excess ligands. This feature is advantageous for optoelectronic applications as well [69,70].

Furthermore, the scope of the reaction was expanded by introducing various substituents at different positions on the phenyl ring. They discovered that electron donating groups in the para position plus an extended conjugated π system were essential for the C–C coupling (dimerization) reaction to occur. Figure 10b illustrates the reaction mechanism, which begins with the formation of the substrate (α-aryl keto nitriles) radical due to the oxidation of photoexcited holes in the PNCs and follows with the C–C bond coupling of neighbouring radicals. Depending on the relative configuration of the two radicals on the NC surface, either *dl*- or *meso*-isomers can be formed. However, less steric hindrance between the aryl groups of the radicals in the *trans*-arrangement and an attractive interaction between the *gauche* cyano groups upon molecular structural relaxation drives the reaction kinetically and thermodynamically to the formation of the *dl*-isomer over the *meso*-isomer.

### 4.4. Synthesis of Gem-Dihaloenones by the Activation of C-Br Bonds of CBrX_3_ (X = Cl, Br)

**Figure 11:**
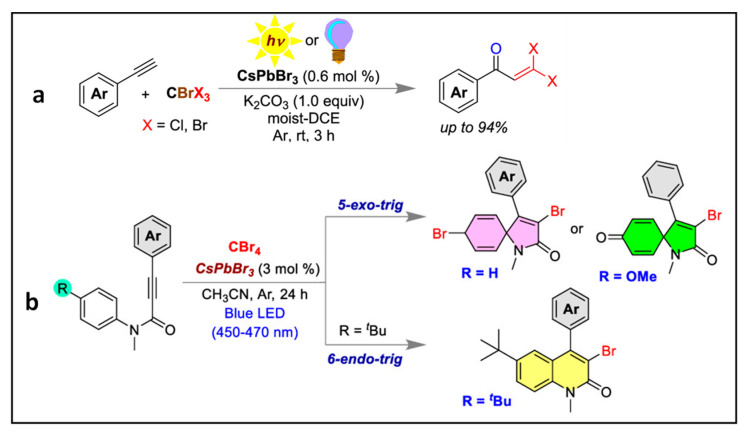
(**a**) Aliphatic C–Br bond activation of CBrX_3_ (X = Br, Cl) using the CsPbBr_3_ NCs. Reproduced with permission. Copyright: 2023, American Chemical Society; (**b**) chemodivergent synthesis of 3,8-dibromo-1-methyl-4-phenyl-1-azaspiro[4.5]deca-3,6,9-trien-2-on or 3-bromo-1-methyl-4-phenyl-1-azaspiro[4.5] deca-3,6,9-trien-2,8-dione or 3-bromo-6-(tert-butyl)-1-methyl-4-phenylquinolin-2(1H)-one using CsPbBr_3_ NCs photocatalyst under visible light irradiation. Reproduced with permission. Copyright: 2023, American Chemical Society.

Mal et al. employed CsPbBr_3_ NCs (PLQY of 93%) for the synthesis of gem-dihaloenones via the activation of C–Br bonds of CBrX_3_ (X = Cl, Br) as shown in Figure 11a, thus achieving a 94% yield of the enone under sunlight [71]. The same group reported chemo-divergent reactions (i.e., obtention of different products by changing the reaction conditions), such as the functionalization of N-methylalkanamides to form 6-endo-trig or 5-exo-trig mode of cyclization via C–Br bond activation of CBr_4_ using CsPbBr_3_ NCs obtained from an unprecedented bromide precursor dibromoisocyanuric acid as shown in Figure 11b. In both cases, an electron transfer takes place from the excited photocatalyst to the reactant to generate a radical anion intermediate.

### 4.5. Aminomethylation of Imidazo-Fused Heterocycles

**Figure 12:**
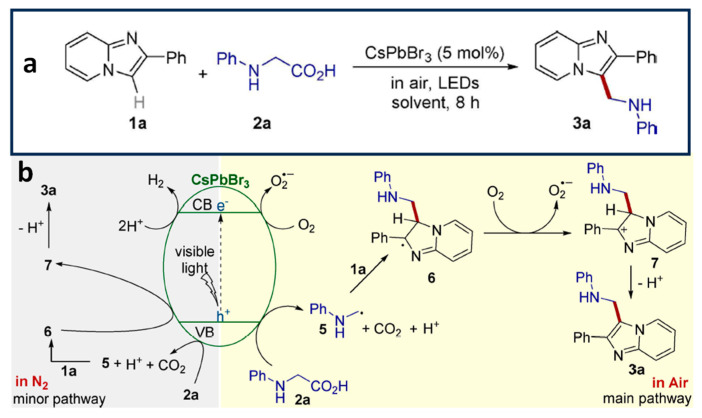
(**a**) Photocatalytic aminomethylation of imidazo-fused heterocycles; (**b**) plausible mechanism of aminomethylation of imidazo-fused heterocycles using CsPbBr_3_ NCs under the irradiation of visible light. Reproduced with permission. Copyright: 2020, John Wiley and Sons.

Shi et al. have reported the direct aminomethylation of imidazo-fused heterocycles through a decarboxylative coupling reaction of N-phenyl glycine (2a in Figure 12a) and imidazo-fused heterocycles (1a in Figure 12a), using octylamine-capped CsPbBr_3_ NCs (P1) [72]. This reaction was carried out under open-air conditions at room temperature and with a high yield (>90%). The mechanism behind this reaction involves radical intermediates. Initially, an electron transfer from 2a (in Figure 12a) to the generated hole results in the formation of intermediate radical 5 (in Figure 12b), accompanied by the release of carbon dioxide and H^+^. Subsequently, the addition of radical 5 (in Figure 12b) to the C=C bond of 1a (in Figure 12a) leads to the formation of radical 6 (in Figure 12b), followed by oxidation to generate radical 7 (in Figure 12b). Eventually, radical 7 (in Figure 12b) undergoes deprotonation to yield the desired product 3a (in Figure 12b). Importantly, the CsPbBr_3_ NCs exhibited excellent recyclability, enabling the reproduction of the product with a high yield for up to five cycles.

### 4.6. Photothermal Suzuki Coupling CsPbBr_3_/Pd

In organic synthesis, the Suzuki coupling reaction is a pivotal method for C–C bond formation. Initially, thermally driven Suzuki coupling reactions utilized Pd complexes as homogeneous catalysts to perform these reactions. Over time, diverse heterogeneous catalysts have been developed to improve such coupling reaction. This was achieved through the integration of photocatalysts, combining Pd NPs with materials such as TiO_2_, SiC, C_3_N_4_, and WS_2_ [73,74,75,76,77]. In this context, Roeffaers et al. employed Pd nanocubes deposited onto CsPbBr_3_ NCs as an efficient photothermal catalyst for Suzuki coupling reaction of iodobenzene with phenylboronic acid as a model reaction under solar irradiation (AM 1.5 G) at 30 °C for 4 h [78]. The 3 wt% Pd/CsPbBr_3_ photocatalyst exhibited the highest conversion, being 11-fold higher than when using CsPbBr_3_ NCs; moreover, it occurred at a nearly 100% selectivity. Furthermore, the 3 wt% Pd/CsPbBr_3_ catalyst displayed an excellent performance for several reactions involving different aryl halides and electron-withdrawing and electron-donating substituents.

Notably, this catalytic performance has been attributed to the synergistic effects of both photoinduced and thermal contributions, with the photothermal activity of 3 wt% Pd/CsPbBr_3_ outperforming both the photoinduced activity of pristine CsPbBr_3_ and the photo-thermal activity of Pd nanocubes at 60 °C, underscoring the unique advantages of this composite. Furthermore, this study elucidated the activation energies of 40.4 and 30.1 kJ mol^−1^ for the thermal and photothermal catalysts, respectively. This information helps to explain the favourable reaction conditions observed in the Suzuki coupling reaction under photothermal catalysis. Stability tests confirmed the sustained activity of the 3 wt% Pd/CsPbBr_3_ composite for up to 24 h.

**Figure 13:**
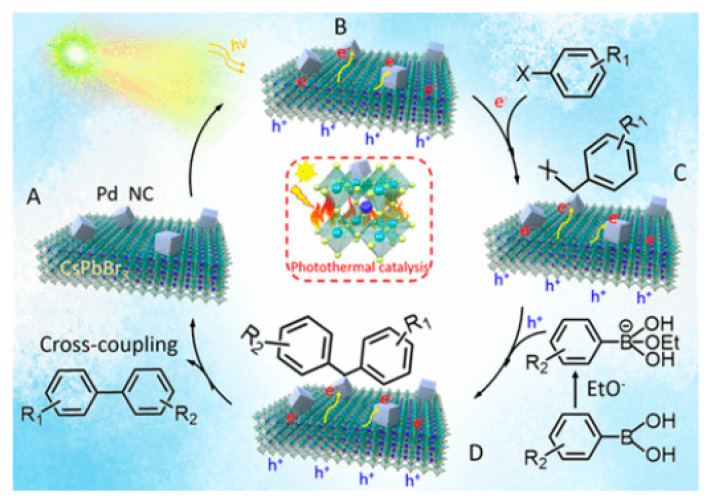
Schematic representation of the sp^2^carbon coupling reaction of iodobenzene with phenylboronic acid using Pd/CsPbBr_3_ Schottky junction photocatalyst. Reproduced with permission. Copyright: 2022, American Chemical Society.

Mechanistic studies corroborated the key role of alkoxides, such as EtO^-^, in triggering the Suzuki coupling reaction. The plausible mechanism involves photoinduced electrons in CsPbBr_3_ transferring to Pd through the Schottky contact [79] as shown in the Figure 13A,B, thus resulting in an electron-rich Pd species that can activate the C–X bond of aryl halides, to produce Pd-adsorbed aryl intermediates (Figure 13C). Simultaneously, phenylboronic acids and alkoxides lead to a boron alkoxide complex, which interacts with photoinduced holes, weakening the carbon–boron bond as shown in the Figure 13C,D. As the activated phenylboronic acid complex migrates to the redox-activated aryl halide, the coupling reaction produces the corresponding biphenyl products (Figure 13A–D).

### 4.7. C–C Bond Cleavage via Decarboxylation and Dehydrogenation Reactions

**Figure 14:**
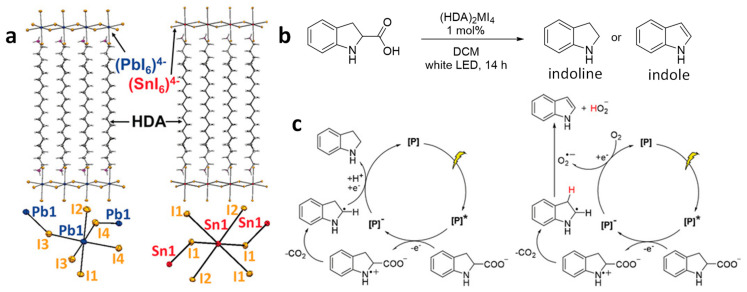
(**a**) Crystal structures of (HDA)_2_PbI_4_ and (HDA)_2_SnI_4_; (**b**) representative decarboxylation and dehydrogenation reactions catalyzed by the (HDA)_2_PbI_4_ and (HDA)_2_SnI_4_ perovskites; (**c**) suggested mechanism for the decarboxylation (left) and dehydrogenation reactions (right). [P] represents the perovskite. Reproduced with permission. Copyright: 2019, John Wiley and Sons.

Soo and co-workers conducted a study involving the C–C cleavage through decarboxylation and dehydrogenation reactions using stable, structurally defined 2D lead and tin iodide perovskites [80]. To enhance the stability of these iodide-based perovskites, they introduced hexadecyl ammonium (HDA) cations as the intercalating layers between the metal halide nanosheets, forming (HDA)_2_MI_4_ (M = Pb, Sn), as illustrated in Figure 14a. It is important to note that the inclusion of the hydrophobic, long HDA cation greatly improves the stability of these (HDA)_2_MI_4_ perovskites in a polar solvent, such as water. However, the stability of (HDA)_2_SnI_4_ is somewhat lower than that of (HDA)_2_PbI_4_. This disparity primarily arises from the susceptibility of Sn^2+^ to oxidize to Sn^4+^. Both 2D perovskites, (HDA)_2_PbI_4_ and (HDA)_2_SnI_4_, exhibit distinct bandgaps, specifically at 2.3 and 1.9 eV, respectively. These bandgap values make them suitable candidates for visible light-driven photochemical reactions.

The authors used these perovskites for decarboxylation and dehydrogenation of indoline-2-carboxylic acid to indoline and indole, respectively, in the presence of O_2_ (Figure 14b). Notably, (HDA)_2_PbI_4_ yielded higher quantities of indoline and indole compared to the lead-free (HDA)_2_SnI_4_. This difference in performance was attributed to the oxidative instability of Sn^2+^ and abundance of traps in the (HDA)_2_SnI_4_ material. Figure 14c provides an explanation of the proposed mechanism, suggesting that photooxidation is the rate determining step in both reactions, with the formation of a superoxide radical playing a crucial role in the dehydrogenation process.

## 5. Doped NCs

Doping serves as a highly effective and versatile strategy in photocatalysis, particularly notable in advancements with halide perovskite photocatalysts. The deliberate introduction of suitable transition metal dopants, through both bulk and surface doping, plays a pivotal role in optimizing photocatalytic processes, notably for CO_2_ reduction and organic synthetic reactions [81]. The success of this strategy lies in its ability to fine-tune material properties, enhancing stability, activity, and selectivity [81]. In the case of CsPbBr_3_, theoretical studies and experimental validations demonstrate that Co and Fe dopants with compatible lattice parameters significantly reduce energy barriers for key reaction steps, showcasing catalytic superiority [82,83]. This enhanced activity is attributed to the adsorption of CO_2_ at doping sites, optimizing the distance to the metal dopant. Experimental confirmation, such as with Fe-doped CsPbBr_3_, further underscores the efficacy of doping in activating photochemical reactions, promoting superior product yields compared to undoped counterparts [84].

### Cu-Doped CsPbBr_3_ NCs for N–N Heterocyclization Reaction

Yan et al. developed a novel photocatalyst that employs transition metal Cu(I) ~ 1% doped CsPbBr_3_ NCs for a *N–N* heterocyclization reaction [85], which is a challenging reaction due to saturated sp^3^ C-based *N–N* rings, which are very labile in nature. The doping of Cu(I) was performed by replacing the Cs atoms on the surface of CsPbBr_3_ NCs. Thus, surface bound Cu(I) ions allow for the accumulation of holes after photoexcitation owing to the fact that their energy levels lie above the VB of CsPbBr_3_ of the NCs.

**Figure 15:**
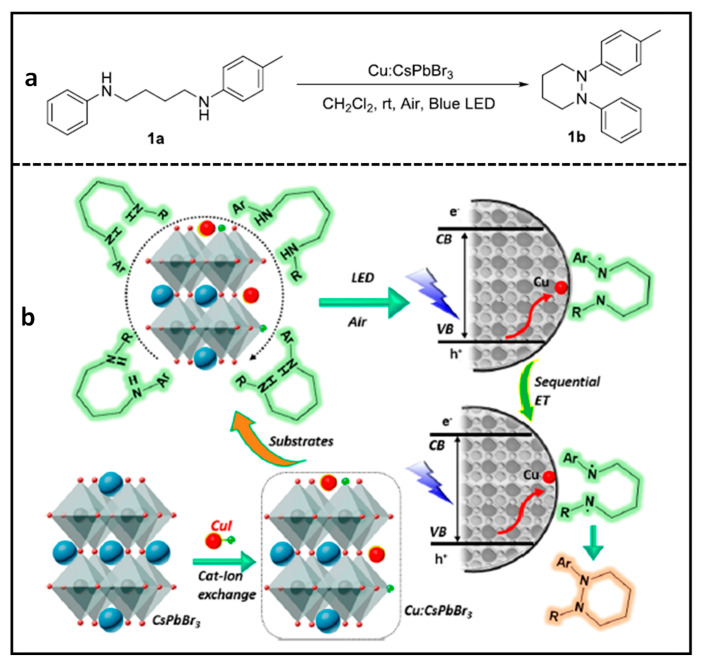
(**a**) Photocatalytic *N*–*N* heterocyclization reaction; (**b**) photocatalytic sequential electron transfer approach for di-radical path N-heterocyclization using Cu-doped CsPbBr_3_ NCs. Red circle represents the Cu(I)-doping in the perovskite crystal structure. Reproduced with permission. Copyright: 2021, American Chemical Society.

These Cu(I)-doped CsPbBr_3_ NC photocatalysts have been used to induce pyridazine formation from reactant 1a (in Figure 15a) with a 90% yield within 18 h, using the conditions mentioned in the reaction. In contrast with a single-carrier transfer in many catalytic reactions, in this reaction, photoinduced holes that were transferred to a diamine substrate form a biradical upon a consecutive inner sphere charge transfer from VB of the NC to surface bound Cu(I) orbitals. Therefore, a multi-charge transfer from the diamine substrate led to the formation of a radical intermediate, which is the key step for the intermolecular biradical formation. As a result, an oxidative intermolecular *N–N* coupling reaction takes place, as shown in the scheme in Figure 15b. It is worth mentioning that product 1b (in Figure 15a) cannot be formed when using a molecular photocatalyst or free Cu in a solution as a co-catalyst. Furthermore, this reaction can be applied to many other aliphatic and aromatic cyclic rings. This study highlights the potential applications of transition metal-doped PNCs in photocatalytic organic synthesis.

## 6. Photocatalytic Polymerization

Poly(3,4-ethylenedioxythiophene) (PEDOT), a widely used conducting polymer in optoelectronic devices, is typically synthesized through the chemical or electrochemical oxidation of 3,4-ethylenedioxythiophene. However, integrating PNCs into a PEDOT matrix using conventional methods poses challenges, risking contamination or damage. Photodeposition, leveraging halide perovskites attributes, offers a clean approach. This process, facilitated by photoinduced electron transfer, enhances optoelectronic properties by efficiently transferring carriers between perovskite QD and the formed polymer matrix.

**Figure 16:**
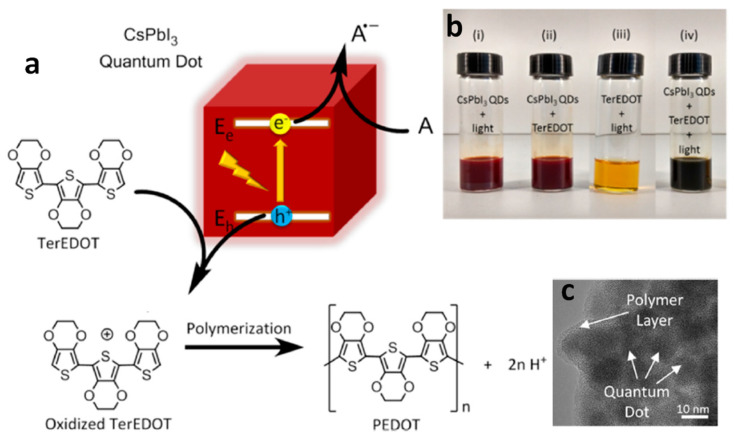
(**a**,**b**) Illustration of the proposed mechanism for photocatalytic polymerization of TerEDOT over CsPbI_3_ NCs under visible light irradiation; (**c**) TEM image of CsPbI_3_ NCs encapsulated by PEDOT and CsPbI_3_ NCs. Reproduced with permission. Copyright: 2017, American Chemical Society.

In this context, Tüysüz et al. were the first to report the photocatalytic polymerization of 2,2’,5’,2”-ter-3,4-ethylenedioxythiophene (TerEDOT) using CsPbI_3_ NCs and visible light illumination [86]. The favourable band alignment between TerEDOT and CsPbI_3_ NCs enabled the transfer of photoexcited holes to TerEDOT, leading to the oxidation of TerEDOT and, ultimately, the formation of poly-(3,4-ethylenedioxythiophene) (PEDOT) in the presence of either benzoquinone or O_2_ as an electron acceptor (Figure 16a). It is noteworthy that, when this reaction is performed in the presence of O_2_, the cubic structure of CsPbI_3_ NCs transformed into an orthorhombic structure, which is unsuitable for optoelectronic applications.

Conversely, in the presence of 1,4-benzoquinone (BQ), the cubic structure of CsPbI_3_ NCs is maintained. Furthermore, the resulting polymer acted as a protective layer, encapsulating the CsPbI_3_ NCs and preserving their cubic structure (Figure 16b). This study emphasizes the utilization of narrow bandgap perovskites for photocatalytic applications. Additionally, Chen and co-workers investigated a similar reaction using CsPbBr_x_I_3−x_ NCs and observed that CsPbI_3_ NCs outperformed CsPbBr_x_I_3−x_ NCs in the photocatalytic polymerization of TerEDOT [87]. This superior performance was attributed to the lower binding energy of CsPbI_3_ NCs (152 meV) compared to CsPbBr_1_._5_I_1_._5_ NCs (472 meV) and CsPbBr_3_ NCs (520 meV). Furthermore, they demonstrated that post-treatment with methyl acetate of oleic acid/oleylamine-capped CsPbBr_x_I_3−x_ NCs accelerates the photocatalytic polymerization of TerEDOT.

## 7. Coupling of Thiols to Disulfides

Disulfides play a crucial role in facilitating the folding of proteins into biologically active conformations [88]. Additionally, they find extensive applications in the fine chemical industry, serving as antioxidants, pesticides, pharmaceuticals, and vulcanization agents [89]. The conventional method for obtaining symmetric disulfides involves oxidative coupling of thiols with oxidants [90]. However, this process often presents challenges, including issues such as over-oxidation; the need for high catalyst-loading, excessive, and costly oxidants; reliance on strong acidic or basic media; and elevated temperatures. The quest for a mild, environmentally friendly, selective, and efficient thiol-coupling protocol remains a significant goal. In this regard, photocatalysis emerges as a gentler pathway to accomplish this crucial transformation [8,91].

**Figure 17:**
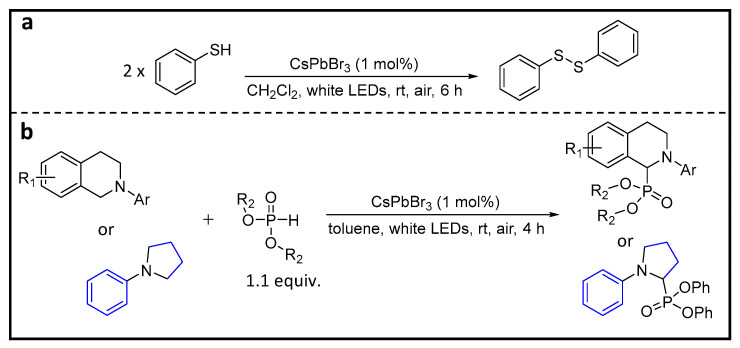
(**a**) Thiol-coupling reaction; (**b**) cross-dehydrogenative coupling reaction between tertiary amines and phosphite esters.

Wu et al. demonstrated the activation of a S–H bond using CsPbX_3_ PNCs [36]. Figure 17 shows the scheme of the oxidative coupling of thiophenol to form the disulfide, using CsPbBr_3_ NCs under the conditions provided in the scheme. Notably, the choice of solvent plays a pivotal role in the reaction success. Changing from CH_2_Cl_2_ to CH_2_Br_2_/toluene/hexane significantly reduces the product yield, likely due to the conversion of the stable CsPbBr_3_ to CsPbCl_3_ (larger bandgap) and its relatively poor stability in various solvent systems. The most optimal solvent combination identified for this coupling reaction is CH_2_Cl_2_/cyclohexane and occurs with a 98% yield. In contrast, the reaction yields for CsPbI_3_ and CsPbCl_3_ are 58 and 12%, respectively. Furthermore, the versatility of this reaction extends to a wide range of substrates, including aromatic, aliphatic, and symmetric and unsymmetric compounds with electron-donating and -withdrawing groups. Remarkably, it consistently provides high yields. The authors proposed plausible mechanisms for this reaction. In this mechanism, thiol S and H coordinates with Pb and Br, respectively. Upon photoexcitation, electrons and holes transfer to H and S, respectively, eventually forming the corresponding radicals. As a result, two thiyl radicals combine to form disulfide, while two H radicals combine to generate H_2_. 

Moreover, researchers have also demonstrated a fascinating cross-dehydrogenative coupling reaction involving tertiary amines, targeting C–H bonds adjacent to amino nitrogen atoms and phosphite esters, as illustrated in Figure 17b.

**Table 1:** Organic transformations photocatalyzed by MHP and their composites/heterostructures compared with conventional semiconductors. C% and S% represent conversion and selectivity, respectively.

Photocatalyst	Irradiation	Organic Transformation/ Photocatalytic Reaction	C (%)	S (%)	Year	Ref
TiO_2_	simulated light irradiation, AM 1.5 G	oxidation of benzylic alcohol	15	95	2018	[92]
FAPbBr_3_	simulated light irradiation, AM 1.5 G	oxidation of benzylic alcohol	15	99	2018	[92]
nano-FAPbBr_3_	simulated light irradiation, AM 1.5 G	oxidation of benzylic alcohol	11	99	2018	[92]
15% FAPbBr_3_/TiO_2_	simulated light irradiation, AM 1.5 G	oxidation of benzylic alcohol	63	99	2018	[92]
15% FAPbBr_3_/SiO_2_	simulated light irradiation, AM 1.5 G	oxidation of benzylic alcohol	13	99	2018	[92]
15% FAPbBr_3_/TiO_2_-M	simulated light irradiation, AM 1.5 G	oxidation of benzylic alcohol	37	99	2018	[92]
15% FAPbBr_3_/TiO_2_	λ ≥ 500 nm	oxidation of benzylic alcohol	13	99	2018	[92]
15% FAPbBr_3_/TiO_2_	without light irradiation	oxidation of benzylic alcohol	0	0	2018	[92]
CdS NCs	2.6 mW 405 nm laser	oxidation of benzylic alcohol	-	99	2019	[93]
(Au@CdS)/Ni	300 W Xe lamp (λ > 420 nm)	oxidation of benzylic alcohol	-	99	2022	[94]
Au_2_S/CdS	6 W blue LED (445 nm)	oxidation of benzylic alcohol	99	100	2022	[95]
g-C_3_N_4_		oxidation of benzylic alcohol		99		[96]
5% NiOx/FAPbBr_3_/TiO_2_	150 W Xe lamp, AM 1.5 G simulated light irradiation	Oxidation of cyclohexane	0.016	>99	2019	[97]
5% NiO_x_/FAPbBr_3_/TiO_2_	150 W Xe lamp, AM 1.5 G simulated light irradiation	Oxidation of cyclooctane	0.032	>99	2019	[97]
CsPbI_3_	Vis LED, 420–700 nm	thiophenol coupled to disulfide	58	-	2018	[36]
CsPbBr_3_	Vis LED, 420–700 nm	thiophenol coupled to disulfide	98	-	2018	[36]
CsPbBr_2_Cl	Vis LED, 420–700 nm	thiophenol coupled to disulfide	98	-	2018	[36]
CsPbBr_0.5_Cl_0.5_	Vis LED, 420–700 nm	thiophenol coupled to disulfide	68	-	2018	[36]
CsPbBrCl_2_	Vis LED, 420–700 nm	thiophenol coupled to disulfide	35	-	2018	[36]
CsPbCl_3_	Vis LED, 420–700 nm	thiophenol coupled to disulfide	12		2018	[36]
CsPbCl_3_	Vis LED, 420–700 nm	thiophenol coupled to disulfide	93	-	2018	[36]
CsPbCl_3_ + Br_2_	Vis LED, 420–700 nm	thiophenol coupled to disulfide	62	-	2018	[36]
Cs_3_Bi_2_Br_9_	visible light ≥ 420 nm	alcoholysis of styrene oxide in isopropanol (IPA)	>99	-	2019	[98]
Cs_3_Bi_2_Br_9_	visible light ≥ 420 nm	alcoholysis of styrene oxide in IPA	>99	-	2019	[98]
CsPbBr_3_	visible light ≥ 420 nm	alcoholysis of styrene oxide in IPA	1	-	2019	[98]
CsPbI_3_	visible light ≥ 495 nm	polymerization of 3,4-ethylenedioxythiophene	32.6	-	2017	[86]
CsPbBr_3_	Blue LED 455 nm	α-alkylation of aldehydes	>99	96	2019	[65]
CsPbBr_3_ NCs	12 W Blue LED, 455 nm	synthesis aldehyde	85	-	2019	[26]
CsPbBr_3_ NCs	12 W Blue LED, 455 nm	synthesis aldehyde	52	-	2019	[26]
CsPbBr_3_ NCs	12 W Blue LED, 455 nm	synthesis tertiary amines	90	-	2019	[26]
CsPbBr_3_ NCs	12 W Blue LED, 455 nm	synthesis tertiary amines	79	-	2019	[26]
CsPbBr_3_ NCs	12 W Blue LED, 455 nm	cyclization of benzaldehyde phenylhydrazone	88	-	2019	[26]
MAPbBr_3_	12 W Blue LED, 455 nm	cyclization of benzaldehyde phenylhydrazone	75	-	2019	[26]
CsPbBr_3_ NCs	12 W Blue LED, 455 nm	cyclization of ethyl (Z)-3-phenyl-3-(phenylamino)acrylate	93	-	2019	[26]
MAPbBr_3_	12 W Blue LED, 455 nm	cyclization of ethyl (Z)-3-phenyl-3-(phenylamino)acrylate	65	-	2019	[26]
CsPbBr_3_ NCs	12 W Blue LED, 455 nm	coupling of benzoic acid with 4-bromotrifluorobenzene	78	-	2019	[26]
CsPbBr_3_	4.6 W Blue LED	photopolymerized styrene	12	-	2018	[99]

## 8. Chiral Perovskite NCs

A material is considered chiral if its mirror image cannot be superimposed onto it. Chirality has been identified, for example, in carbohydrates and amino acids (except for glycine). Chiral catalysts have found extensive use in the asymmetric synthesis of pharmaceuticals. Recently, researchers have explored chiral MHPs to manipulate charge, light, and spin for applications in chiral optoelectronics, ferroelectrics, spin LEDs, spintronics, solar cells, and more [100]. Hybrid perovskites offer more exciting properties compared to inorganic- and organic-based perovskites, including exceptional diffusion lengths and fewer defects. Additionally, introducing chiral organic ligands into the perovskite crystal structure can induce chirality. The transfer of chirality can take place through the establishment of chemical bonds or even via spatial interactions between a chiral and an achiral system.

**Figure 18:**
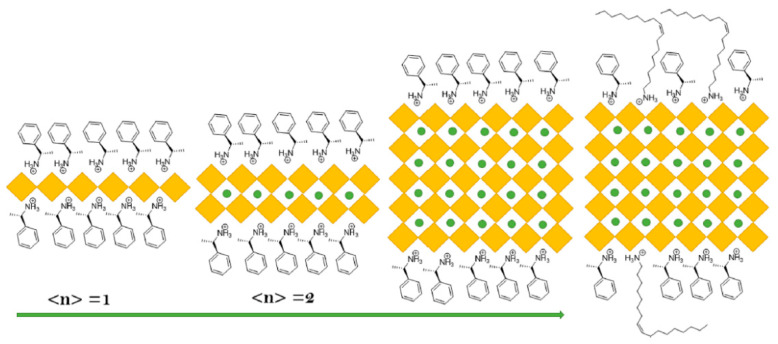
Chiral perovskite NCs. NCs formulated with L_2_(APbBr_3_)*_n_*_−1_PbBr_4_. Right: proposed structure of NCs used in this study where *n* = ∞. Reproduced with permission. Copyright: 2023, American Chemical Society.

Reduced dimension perovskites can be represented by L_2_(APbBr_3_)_n-1_PbBr_4_ where L is surface organic chiral ligands, and A is a monovalent cation; n represents the number of inorganic layers between chiral organic ligands. Colloidal PNCs can be prepared with layers from n = 1 to ∞ by controlling the ration of L, A, and PbBr_2_, schematically shown in the Figure 18; L can be the R- or S-1-phenylethylammonium cation. Notably, the surface properties can be adjusted by the introduction of distinct chiral ligands.

### Production of N–C Axial Chiral N-Heterocycles Using Chiral CsPbBr_3_ NCs

Asymmetric catalysis requires the presence of a chiral-NC surface. Earlier research suggests a specific set of chiral ligands (ammonium-based) for perovskites. These chiral ligands are introduced to the perovskite either through direct synthesis or via post-synthetic ligand exchange. In the case of direct synthesis, chiral ligands impact the crystal structure, while post-synthesis incorporation leads to chirality due to the distortion of the NC surface. Recently, Yan et al. have demonstrated asymmetric photochemical synthesis using R-PEA hybridized perovskite PEA/CsPbBr_3_ NCs as photocatalysts [101]. These NCs were able to oxidize N-arylamine and produce N–C axially chiral N-heterocycles (N-arylindoles) under visible light. A range of R-PEA/CsPbBr_3_ NCs were synthesized using emulsion synthesis, hot injection, or high-energy tip sonication methods. Among them, the tip sonication method exhibited the highest coverage of chiral ligands. The PNC surfaces were coated with both chiral ligands and non-chiral oleylammonium ligands, as depicted in Figure 18.

**Figure 19:**
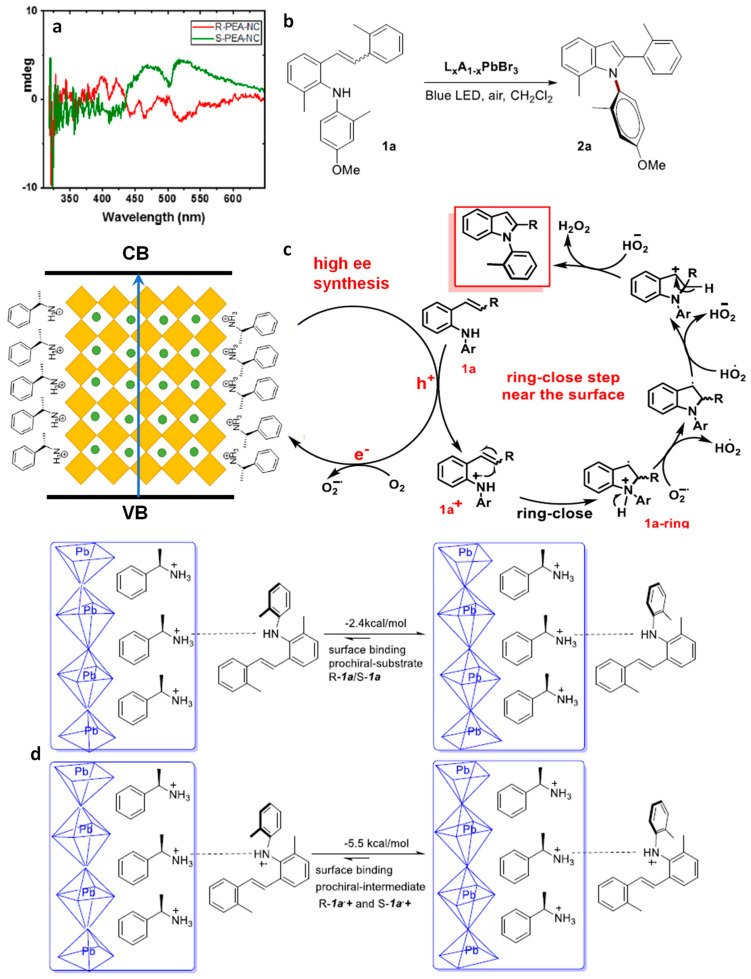
(**a**) Circular dichroism (CD) spectrum of R-NC-3 and S-NC-3; (**b**) illustration of N–C axially chiral atroposelective N-heterocyclization using L_2_(APbBr_3_)*_n_*_−1_PbBr_4_ photocatalyst; (**c**) proposed mechanism for N-arylindole; (**d**) DFT studies, binding energy discrepancies between the chiral surface and substrate 1a (up), 1a+ (after hole transfer). Reproduced with permission. Copyright: 2023, American Chemical Society.

Typically, optically active compounds are assessed using circular dichroism (CD) spectroscopy. The chiral R- and S-forms of CsPbBr_3_ NCs displayed distinct CD signals at 515 nm and demonstrated anisotropy near the bandgap, as illustrated in Figure 19a. This CD response denotes the electronic interaction between the perovskite NC lattice and the chiral ligand. Chiral PEA/CsPbBr_3_ NCs were employed for the conversion of 1a to 2a (in Figure 19). Interestingly, a lower surface coverage with chiral ligands (9%, 17%, and 47%) yielded a higher enantiomeric excess (ee), specifically, 63%, 83%, and 99%, respectively. This suggests that a greater surface coverage enhances enantioselectivity.

Figure 19c presents the proposed mechanism for the formation of highly enantioselective *N*-arylindoles; the substrate 1a approaches the chiral NC surface via the weak hydrogen bonding between the *N*-arylindol 2a and the enantiomerically pure PEA. After light excitation, the photogenerated holes transfer to 1a, resulting in 1a+, which undergoes ring closing on the surface-bound prochiral substrate (in Figure 19c).

Density functional theory (DFT) calculations were conducted to differentiate the preferred attachment of R- and S-1a substrates to the R-PEA chiral surface of CsPbBr_3_ NCs. These calculations revealed a −2.4 kcal/mol preferred binding energy of prochiral N-arylamine (1a) with R-configuration compared to its enantiomeric S-configuration counterpart, binding weakly through H-bonding to the R-PEA chiral surface (in Figure 19d). This suggests that the discrimination step occurs at the beginning of the catalytic cycle. Additionally, differences in surface binding energy after a photoinduced hole transfer indicated a −5.5 kcal/mol preferred binding energy of prochiral-R-1a+ over the prochiral-S-1a+ intermediate (in Figure 19d). Interestingly, during the ring-closing step, both R- and S-forms exhibited the same transition energy. Overall, the binding preference of R-PEA chiral surface of the CsPbBr_3_ NCs governs the N–C axial chirality of the N-heterocycles.

## 9. Triplet Energy Transfer from Dyes and NCs to Substrates

**Figure 20:**
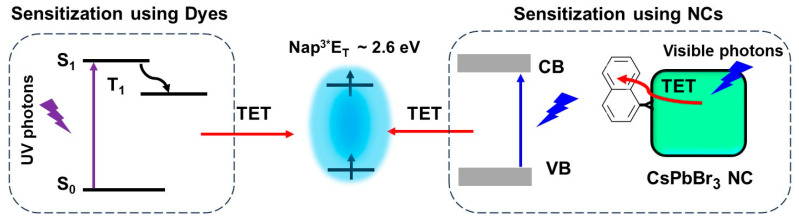
(**Left**) Sensitization of naphthalene triplets (Nap^3*^) using dyes: light absorption populates the first singlet excited state (S_1_), which is followed by intersystem crossing (ISC) to generate the first triplet states (T_1_). These T1 states can subsequently transfer their energy to Nap via Dexter-type triplet energy transfer (TET) mechanism. Considering that the energy of Nap^3*^ is ∼2.6 eV and that there is an estimated energy loss of ∼0.5 eV during the ISC process, it implies that the energy of S_1_ of the dye must be at least ∼3.1 eV, indicating that UV photons are required to drive the sensitization process; (**Right**) Sensitization of naphthalene triplets (Nap^3*^) using semiconductor CsPbBr_3_ NCs: light absorption leads to the generation of band edge excitonic states (X) within the NCs. These excitonic states can directly undergo TET to produce Nap^3*^. This means that visible photons are capable of efficiently driving the sensitization process.

Polycyclic aromatic hydrocarbons (PAHs) are organic compounds extensively explored for their singlet and triplet states in various applications, including photocatalysis, solar energy, light emission, photon up-conversion, and room temperature phosphorescence [102,103,104,105,106]. Nonetheless, the direct generation of their triplet state through optical excitation proves to be challenging due to their inherently dark nature and the inefficiency of intersystem crossing (ISC). Therefore, a sensitization approach has been adopted to generate PAH triplets, employing sensitizers, such as boron-dipyrromethane (BODIPY) dyes and fullerenes [107,108,109]. These sensitizers absorb light to populate singlet states, which then efficiently undergo ISC to populate triplets. Subsequently, these triplets can transfer their energy to PAHs (acceptor) through Dexter-type triplet energy transfer (TET), as illustrated on the left side of Figure 20. However, during the ISC process, there can be an intrinsic energy loss in the order of 0.5 eV or more, which leads to practical issues in visible-to-UV light up-conversion applications [110].

Semiconductor NCs present significant benefits compared to molecular photosensitizers due to their easily achievable preparation, robust photostability, size-dependent electronic and photophysical characteristics, high molar extinction coefficients, and simple post-synthesis functionalization [111]. Moreover, the minimal energy separation of 1 to 15 meV between bright and dark states, as opposed to the singlet–triplet splitting observed in organic molecules, renders NCs promising candidates for the sensitization process at the limit of energy conservation, driven by visible photons [111,112]. Among the diverse arrays of NCs, PNCs have excellent optical properties, such as tuneable bandgaps in the UV–visible region, near unity PL QY, and potential as a triplet photosensitizer [113]. These attributes position PNCs as promising candidates for generating triplet states in target substrates. On the right side of Figure 20, an illustration shows the sensitization of naphthalene using confined CsPbBr_3_ NCs, which possess a bandgap exceeding 2.6 eV, effectively aligning with the triplet energy (E_T_) of approximately 2.6 eV [114]. To achieve efficient TET from NCs to acceptors, the NC bandgap should be greater than the acceptor triplet energy level to be in resonance.

### 9.1. Demonstration of TET from Quantum Confined PNCs to Substrates

The utilization of visible light captured by long-lived triplet excited states has found widespread application in photocatalytic organic synthesis and photon up-conversion across various spectral ranges. An effective strategy involves storing the excitation energy of NCs in long-lived molecular triplet states through TET to PAHs. In this context, the combination of PNCs and PAHs allows for efficient TET, leveraging the strong light-harvesting capability of PNCs and surpassing the short emission lifetimes. This combination facilitates effective energy transfer, enabling the utilization of long-lived triplet states for desired applications in photon up-conversion and other related fields [115]. For example, in a study conducted by Wu et al., the investigation focused on the size-dependent carrier density probability at the surface of CsPbBr_3_ NC and its linear correlation with TET rates [116]. They employed CsPbBr_3_ NCs with varying edge lengths (L), ranging from 11.2 to 3.5 nm, resulting in bandgap variations from 2.43 to 2.73 eV. Triplet acceptor 1-pyrenecarboxylic acid (PCA) was introduced due to its triplet energy (E_T_) of 2 eV and favourable band alignment with CsPbBr_3_.

**Figure 21:**
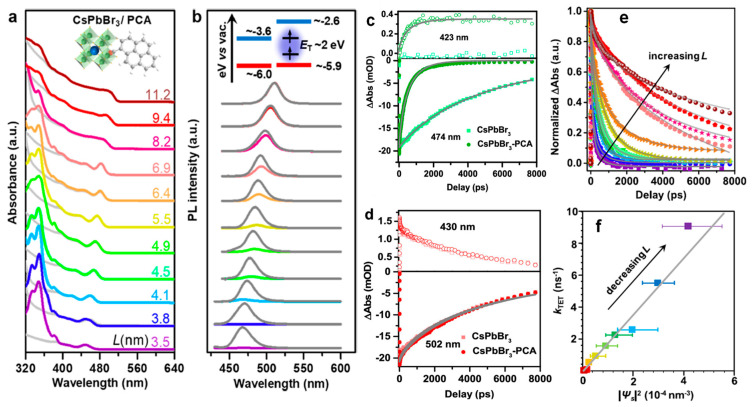
(**a**) Absorption spectra of CsPbBr_3_ NCs (grey solid lines) and NC–PCA complexes (coloured solid lines) with different NC sizes (edge length *L* indicated) dispersed in hexane. The variation between them is due to PCA absorption. The inset illustrates the schematic structures of NCs and PCAs; (**b**) PL spectra of CsPbBr_3_ NCs (grey solid lines) and NC–PCA complexes (coloured solid lines) excited at 420 nm. The inset illustrates the schematic energy level alignment between NCs and PCAs; (**c**) TA kinetics probed at the exciton bleaching (X_B_) centre (∼474 nm; solid symbols) and at the PCA triplet absorption (∼423 nm; open symbols) of L = 4.9 nm NCs (squares) and NC–PCA complexes (circles). The grey solid lines represent their fits using stretched-exponential functions; (**d**) Similar plot as in c for L = 9.4 nm NC–PCA complexes; (**e**) Normalized TA kinetics probed at the X_B_ feature of NC–PCA complexes with varying NC sizes (coloured symbols). The grey solid lines represent their fits; (**f**) size-dependent TET rate (*k*_TET_) plotted as a function of carrier probability density at the NC surface (|Ψ_s_|^2^) for different NC sizes (coloured symbols). The grey solid line depicts a linear fit. Reproduced with permission. Copyright: 2019, American Chemical Society.

All CsPbBr_3_ NCs in the size range of 11.2 to 3.5 nm possessed bandgap energies higher than the ET of PCA, theoretically enabling TET. To explore size-dependent TET, PCA was grafted onto various-sized CsPbBr_3_ NCs via a carboxylic group. Figure 21a shows the absorption spectrum of the CsPbBr_3_-PCA complex (1:1 ratio of different-sized CsPbBr_3_ NCs and PCA), which was compared to that of pristine CsPbBr_3_ NCs, revealing a blue-shift in the lowest-energy absorption peak due to QCE as L decreased from 11.2 to 3.5 nm.

In the analysis of the CsPbBr_3_-PCA complex, the absorption spectrum, depicted by the coloured line in Figure 21a, reflects features from both the CsPbBr_3_ NC and PCA (with PCA absorbing below the 400 nm region). To delve into the mechanism of TET from CsPbBr_3_ NCs to PCA molecules, steady-state PL for the CsPbBr_3_-PCA complex was systematically examined. Figure 21b showcases the steady-state PL spectra of CsPbBr_3_ NCs and the CsPbBr_3_-PCA complex under selective excitation of NCs at 420 nm.

Notably, the PL quenching efficiency witnessed a remarkable increase from 0.6% for bulk-like NCs with an L of 11.2 nm to an impressive 99% for the more strongly confined 3.5 nm-sized NCs. The energy level diagram presented in the schematic inset of Figure 21b elucidates that this substantial PL quenching can be attributed to the efficient TET process from the CsPbBr_3_ NCs to the PCA molecule. This compelling finding underscores the efficacy of TET, particularly in the context of strongly confined CsPbBr_3_ NCs.

Transient absorption spectroscopy (TAS) was employed to monitor TET dynamics. Figure 21c show the kinetics of exciton bleaching (X_B_) in free NCs and NC–PCA complexes. This indicated a significant shortening of the X_B_ lifetime in NC–PCA complexes, accompanied by the appearance of an absorption feature at ~ 423 nm corresponding to the T_1_ → T_n_ transition of PCA triplets. This provided clear evidence of ultrafast TET from NCs to PCAs.

The ultrafast quenching of NC excitons is conclusively attributed to TET from NCs to PCAs. Intriguingly, this ultrafast TET stands in stark contrast to the negligible TET observed in 9.4 nm bulklike NCs, as depicted in Figure 21d. The X_B_ lifetimes in both free NCs and NC–PCA complexes remained similar, and no discernible triplet absorption feature was identified (Figure 21d). This striking disparity in behaviour between small- and large-sized NCs underscores the pivotal role played by the QCE in facilitating efficient TET.

Figure 21e presents the normalized kinetics of the exciton bleaching feature (X_B_) for various CsPbBr_3_-PCA complexes utilized in the study. Notably, the TET rate demonstrates a clear trend increasing with the decreasing size of NCs. Particularly, the strongly confined 3.5 nm NCs exhibit a notably higher TET rate. The QCE primarily influences the energetics (driving force and spectral overlap) and electronic coupling involved in the TET process, particularly if TET follows the well-established Dexter mechanism. To unravel the effect of the driving force and spectral overlap, the TET was examined using similar-sized NCs with varying emission peaks achieved by altering x in the CsPbBr_3−x_Cl_x_ NCs. Intriguingly, NC–PCA complexes with different x values displayed remarkably similar X_B_ recovery kinetics, suggesting that their TET kinetics should also align. This implies that spectral overlap and driving force play negligible roles in the TET for this system. Consequently, the inference is that quantum confinement primarily influences TET through electronic coupling.

Smaller NCs with stronger QCE are known to possess higher carrier probability densities at their surfaces, leading to stronger electronic coupling with attached acceptors. To understand the size-dependent TET rate (k_TET_), the carrier probability density at the CsPbBr_3_ NC surface denoted as |Ψs|^2^ was plotted against k_TET_, revealing an increase from nearly 0 for 11.2 nm-sized NCs to 9.1 ± 0.1 ns^−1^ for 3.5 nm-sized NCs. The linear fitting of this data suggests that the wave function exchange between CsPbBr_3_ NCs and PCA molecules is the dominant factor governing this Dexter-type mechanism. Notably, the absence of a hole transfer pathway from NCs to PCA in this study further underscores the impact of QCE on efficiently mediating TET to PCA molecules with decreasing NC size.

### 9.2. Isomerisation of Surface-Anchored Stilbene

**Figure 22:**
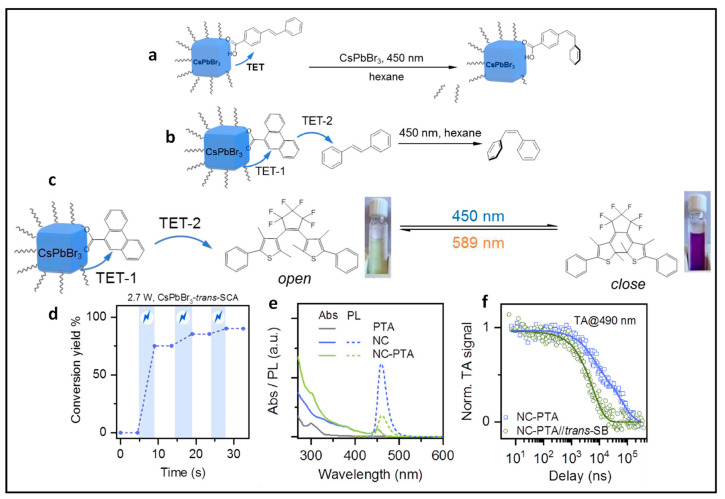
(**a**) Photoisomerization of surface tethered stilbene; (**b**) the 9-phenanthrene carboxylic acid (PTA) ligands are anchored on CsPbBr_3_ NCs surfaces, whereas the stilbene molecules are freely diffusing in solution; (**c**) photoisomerization of diarylethene; (**d**) light-modulated conversion yields under 2.7 W 450 nm illumination, the blue shaded areas correspond with light-on; (**e**) absorption (solid lines) and PL (dashed lines) spectra of CsPbBr_3_ NCs with (green) and without (blue) surface-anchored PTA ligands; (**f**) nanosecond-TA kinetics probed for NC-sensitized ^3^PTA* with (green open circles) and without (blue open squares) trans-stilbene in the solution. Reproduced with permission. Copyright: 2022, John Wiley and Sons.

Wu et al. demonstrated visible light-driven molecular isomerization and cycloaddition reactions via the TET approach from CsPbBr_3_ and CsPbBr_3−x_I_x_ PNCs to the substrates [117]. To showcase photoisomerization, the authors selected trans-4-stilbenecarboxylic acid (trans-SCA), whose carboxylic group anchors to the of PNC surface. The stilbene triplet energy (E_T_) is ~2.13 eV for the trans isomer and ~2.47 eV for the cis isomer. Consequently, 3.5 nm-sized CsPbBr_3_ NCs with an excitonic peak at approximately 455 nm (2.73 eV) were chosen to drive the reaction via TET. Figure 22a illustrates the isomerization of trans SCA to cis SCA in hexane under 5 mW 450 nm illumination. The conversion yield (CY) was calculated based on the absorption coefficients of trans- and *cis*-stilbene. An illumination power of 2.7 W enabled the reaction to reach the steady state within 15 s. The light-modulated CY is depicted in Figure 22d, showing an increase in CY under illumination, ultimately reaching 90.1%. Furthermore, when photoisomerization was conducted with CsPbBr_3−x_I_x_ NCs, whose bandgap slightly exceeded the ET of *trans*-SCA (approximately 2.25 eV), it was intriguingly transformed to cis-SCA at a slower rate than that of CsPbBr_3_ NCs.

### 9.3. Isomerization of Stilbene in Solution Using Relay

Isomerization of organic molecules in a free form, i.e., non-anchored onto the NC surface, was also possible using surface-anchored triplet transmitters, which act as a relay to accept energy from photoexcited NCs and pass it to the annihilator in solution. Wu et al. demonstrated that 9-phenanthrene carboxylic acid (PTA) with ET of ~2.64 eV as the transmitter ligands showed the TET from 3.5 nm-sized CsPbBr_3_ NCs to trans-stilbene as shown in Figure 22b. Figure 22e shows the absorption and PL spectra of CsPbBr_3_ NCs as well as those of PTA-anchored CsPbBr_3_ NCs. A significant PL quenching (69.2%) was observed in the presence of PTA ligands. In Figure 22f, TA kinetics show that the average lifetime (~42 µs) of CsPbBr_3_ NC sensitized ^3^PTA* shortened to ~5.8 µs for the complex of NC-PTA and trans-stilbene. This indicates an efficient TET-2 (86.1%) from ^3^PTA* to stilbene. This reaction was performed under 2.7 W 450 nm illumination; the CY was of 82.7% in 30 s. Thus, TET and photoisomerization can be comparable between surface-tethered and relayed systems.

Additionally, authors used the relay method to demonstrate the ring-closing isomerization of diarylethene (DAE), as shown in Figure 22c. The DAE open form can be converted into the DAE closed form via efficient TET-2 from the ^3^PTA* to DAE open form. This could be clearly seen since the colour changed from colourless to purple, corresponding to their absorption at of 270 nm and 565 nm of DAE open and closed forms, respectively. However, control experiments failed to convert the open-to-closed form with direct 450 nm laser illumination. Notably, the selective excitation of the DAE closed form with a 589 nm laser induced the conversion to the open form. This reversible photochromic reaction finds utility in applications such as light-induced information coding and patterning.

The relay method can also be extended to intermolecular [2 + 2] cycloaddition reactions of acenaphthylene (ACN) to dimerization. The ET of ACN was in the range of 1.87–1.95 eV, which is lower than that of PTA ligands.

### 9.4. Photocatalytic 2 + 2 Cycloadditions

**Figure 23:**
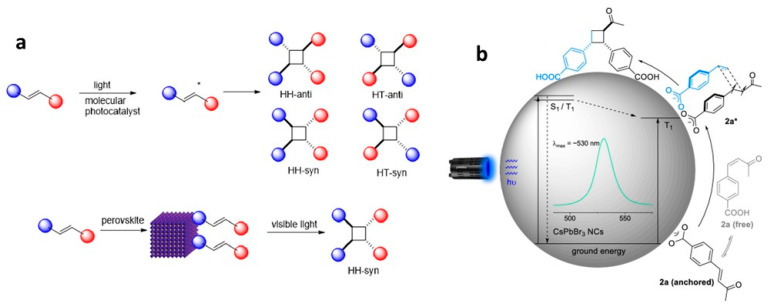
(**a**) Molecule- and perovskite-induced photocatalysis for TET based 2 + 2 cycloaddition. HH: head-to-head, HT: head-to-tail; star represents the excitation of the molecular photocatalyst; (**b**) proposed mechanism for the intermolecular 2 + 2 cycloaddition facilitated by CsPbBr_3_ NCs through TET induced by visible light. Reproduced with permission. Copyright: 2022, American Chemical Society.

The direct utilization of long-lived triplet states using molecular photocatalysts for organic molecule activation has been extensively explored. This often entails the TET from these molecular photocatalysts to organic substrates, inducing non-selective 2 + 2 cycloaddition reactions, as depicted in Figure 23a. In contrast, using PNC surfaces as templates, dynamically anchored substrates facilitated efficient TET to the substrate and achieved regioselective head-to-head (HH) 2 + 2 cycloaddition reactions in a *syn*-mode. This *syn*-binding mode promotes the formation of thermodynamically unfavourable 2 + 2 *syn*-cyclobutane products, a feat beyond the realm of molecular photocatalysis. Yan et al. demonstrated the photocatalytic synthesis of highly diastereomeric *syn*-selective 2 + 2 olefin cycloadditions using bulk-like PNCs as photosensitizers via TET [118]. Figure 23b exemplifies the photocatalytic 2 + 2 *syn*-cycloaddition reaction for substrate 2a (in Figure 23b) under optimized conditions, illustrated in the Figure 23b.

The anchoring of the substrate to the PNC surface through a carboxylic group along with its lower triplet energy than that of bulk-like PNCs mediated the TET. Comparative studies revealed that the same reaction with an Ir(ppy)_3_ molecular photocatalyst yields a mixture of products, with HH-anti and HH-*syn* as major and minor products, respectively, with trace amounts of HT-anti and HT-*syn* products, where the triplet energy acceptor remains in a free (unbound) state. Moreover, the applicability of photocatalytic 2 + 2 cycloadditions can be broadened to encompass both homo- and hetero-variations involving other substrates possessing triplet energies lower than those of PNCs.

## 10. Benzaldehyde Formation from Oxidation of Benzyl Alcohol and Toluene

The efficient and selective oxidation of saturated C–H bonds in alkanes or aromatics, such as toluene, leading to the generation of aromatic aldehydes and ketones, including benzyl alcohol, benzaldehyde, benzoic acid, and benzyl benzoate, serve as versatile intermediates for the production of pharmaceuticals, dyes, solvents, perfumes, plasticizers, dyestuffs, preservatives, and flame retardants [119]. The inherent thermodynamic strength and dynamic inertness of saturated C–H bonds necessitate harsh reaction conditions, involving high temperatures, high pressures, and the use of extra initiators or toxic reagents, in traditional methods [120,121,122]. Recognizing the environmental impact of such conventional processes, there is a growing emphasis on the development of green chemistry approaches, such as heterogeneous photocatalysis, that provide environment-benign routes for the conversion of alkanes or aromatics into value-added products.

### Benzaldehyde Formation Using Pb-Based Perovskite Heterostructures as Photocatalyst

The success of LHP nanoparticles (NPs) in various optoelectronic applications has led to their utilization in photocatalytic applications. Initial studies focused on simple organic molecule transformations, such as the formation of benzaldehyde through the oxidation of benzyl alcohol and toluene, owing to the favourable band alignment of LHPs. First, we would like to discuss the formation of benzaldehyde through the oxidation of benzyl alcohol, followed by that of toluene. The energy band structure of LHPs is well suited for the oxidation of benzyl alcohol. However, studies involving LHPs, such as FAPbBr_3_ and CsPbBr_3_, for benzyl alcohol oxidation, have shown a conversion rate of ~15% with a selectivity >99% under visible light illumination. The conversion rate and selectivity can be calculated using the following formula:

$$\mathrm{Conversion}\;(\%)=\left(c_{B}-c_{A}\right)/c_{B}\times 100$$

$$\mathrm{Selectivity}\;(\%)=c_{p}/(c_{B}-c_{A})\times 100,$$

where $c_{B}$, $c_{A}$, and $c_{p}$ are the concentration of reactant before and after the reaction, and the amount of target product, respectively.

**Figure 24:**
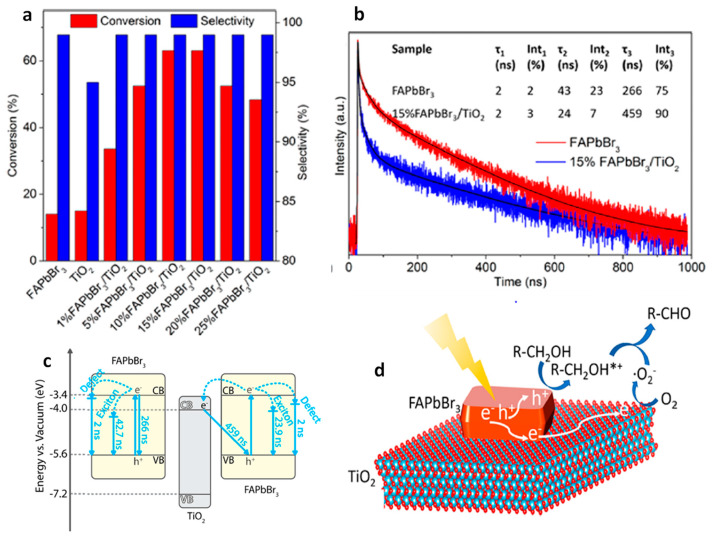
(**a**) Investigation of the photocatalytic oxidation of benzyl alcohol using pure FAPbBr_3_, TiO_2_, and a series of FAPbBr_3_/TiO_2_ hybrids; (**b**) analysis of PL decay spectra of the prepared FAPbBr_3_ and 15% FAPbBr_3_/TiO_2_ and their multiexponential fitting parameters; (**c**) experimentally supported mechanistic energy diagram of photoinduced charge transfer in FAPbBr_3_ and FAPbBr_3_/TiO_2_ hybrid; (**d**) schematic of the proposed selective photocatalytic oxidation of benzyl alcohol to benzaldehyde using the FAPbBr_3_/TiO_2_ hybrid. Reproduced with permission. Copyright: 2022, American Chemical Society.

Despite of having a suitable band structure, the relatively poor performance of LHPs might be attributed to the strong recombination of photogenerated carriers within the LHPs. Consequently, heterojunctions, such as type-II, direct-Z-scheme, and Schottky, were developed to significantly enhance the photocatalytic activity of LHPs. For example, Tüysüz and co-workers prepared CsPbBr_3_/TiO_2_ heterostructures by growing smaller-sized CsPbBr_3_ NCs (2–4 nm) onto the TiO_2_ NPs (P25). This resulted in a type-II band alignment at the interface of CsPbBr_3_ and TiO_2_, thus enhancing the conversion rate of benzyl alcohol up to 50% with a selectivity >99% [61]. Moreover, Roeffaers and co-workers improved the conversion rate of benzyl alcohol up to 63% (Figure 24a) without compromising the selectivity [92]. They achieved this by depositing FAPbBr_3_ particles onto TiO_2_ nanosheets, which help in the effective separation of charges due to type-II band alignment of FAPbBr_3_/TiO_2_. Additionally, TiO_2_ nanosheet morphology provides a large number of active catalytic sites. Figure 24b shows the PL lifetime decay profile, showing a faster decay for 15% FAPbBr_3_/TiO_2_ compared to FAPbBr_3_, thereby suggesting efficient charge carrier dissociation. The fitting parameters (shown in the inset of Figure 24b) indicated a decrease in the exciton PL lifetime (~43 to 24 ns) and an increase in free carrier lifetime contribution (from 266 to 459 ns) for the 15% FAPbBr_3_/TiO_2_ composite, indicating an efficient exciton dissociation at the interface of FAPbBr_3_/TiO_2_, as shown in the Figure 24c. As a result, FAPbBr_3_/TiO_2_ heterostructures exhibited a four-fold enhancement of photocatalytic conversion (63%) of benzyl alcohol with high selectivity (99%) compared to pristine FAPbBr_3_ and TiO_2_. In another study, Luo et al. used g-C_3_N_4_ as an electron extraction layer to establish a type-II band alignment with FAPbBr_3_ NPs. The resulting FAPbBr_3_/C_3_N_4_ heterostructures enhanced the conversion of benzaldehyde from 15% to ~46% with 99% selectivity [123].

Figure 24d shows the mechanism of the formation of benzaldehyde. The excitation of FAPbBr_3_/TiO_2_ heterostructures with visible light generates a photoexcited electron in the CB, leaving behind holes in the VB. The electron can then transfer to the low-lying CB of TiO_2_ due to favourable energy level alignment, either diffusing to the surface or being directly injected into O_2_, eventually leading to the formation of superoxide radicals (O_2_^•−^) in both scenarios. However, it should be noted that O_2_^•−^ formation cannot be ruled out when O_2_ directly interacts with the surface of FAPbBr_3_. Simultaneously, the photogenerated hole in the VB of CsPbBr_3_ is refilled by an electron from the alcohol, resulting in the creation of the alcohol radical cation, which subsequently reacts with the superoxide radical to generate the corresponding aldehyde, as shown in Figure 24d.

The type-II band alignment strategy has also been studied for the toluene oxidation. Li et al. demonstrated that the introduction of Cl into CsPbBr_3_/TiO_2_ heterostructures through anion exchange resulted in the formation of asymmetric Cl^−^ distribution in CsPbBr_x_Cl_3−x_ [124]. As a consequence, a funnel-like band structure with a Br-rich core and Cl-rich surface was formed, enhancing the photocatalytic activity of toluene oxidation compared to that of CsPbBr_3_/TiO_2_. Remarkably, the optimized CsPbBr_x_Cl_3−x_/TiO_2_ composite exhibited a benzaldehyde formation rate of 1874 µmol·g^−1^·h^−1^, which is approximately 1.5 times higher in performance compared to that of CsPbBr_3_/TiO_2_. In contrast, the CsPbBr_x_Cl_3−x_/TiO_2_ prepared with direct synthesis, with uniformly distributed Cl^−^, showed a maximum benzaldehyde conversion of 1625 µmol·g^−1^·h^−1^, which is 1.15 times lower than the anion exchange samples. The enhanced activity has been attributed to the shift in the VB with increasing Cl content in CsPbBr_3_/TiO_2_ and the unique graded band structure.

Zhu et al. used CsPbBr_3_/TiO_2_ heterostructures for toluene oxidation. Interestingly, a temperature increase enhanced the activity of toluene and benzyl alcohol oxidization. CsPbBr_3_/TiO_2_ composite showed the production rate of benzaldehyde gradually increased from 1089 µmol·g^−1^·h^−1^ at 45 °C with a selectivity of 84.2% to 2356 µmol·g^−1^·h^−1^ at 75 °C and with a selectivity of 80.3%; this value was four times higher than that of pristine CsPbBr_3_ NCs and three times higher than that of TiO_2_ NPs [125]. Moreover, the production rate of benzyl alcohol increased from 205 to 578 µmol·g^−1^·h^−1^ at 45 °C and 75 °C, respectively.

**Figure 25:**
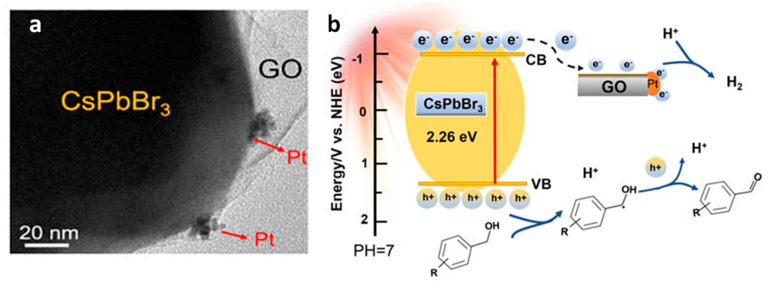
(**a**) TEM image of CsPbBr_3_/GO-Pt composite; (**b**) schematic illustration of photocatalytic H_2_ production integrated with benzaldehyde synthesis. Reproduced with permission. Copyright: 2022, Frontiers Media S.A.

Qian et al. demonstrated a Schottky junction by decorating CsPbBr_3_ with graphene oxide and platinum (GO-Pt) catalyst for preventing the recombination of excitons, as depicted in Figure 25a, to facilitate benzyl alcohol conversion and H_2_ generation [126]. In this setup, GO plays a crucial role in structurally directing the CsPbBr_3_ particles, leading to a reduction in their size compared with pristine CsPbBr_3_ NCs. Moreover, GO-Pt acts as an electron reservoir, effectively extracting the photoinduced electrons, which can be used for H_2_ formation. Additionally, photoinduced holes are involved in the oxidation of benzyl alcohol to benzaldehyde, as illustrated in Figure 25b. The optimal amount of CsPbBr_3_/GO-Pt exhibits 1050 µmol·g^−1^·h^−1^ of benzaldehyde formation with >99% selectivity and 1060 µmol·g^−1^·h^−1^ of H_2_ generation.

**Figure 26:**
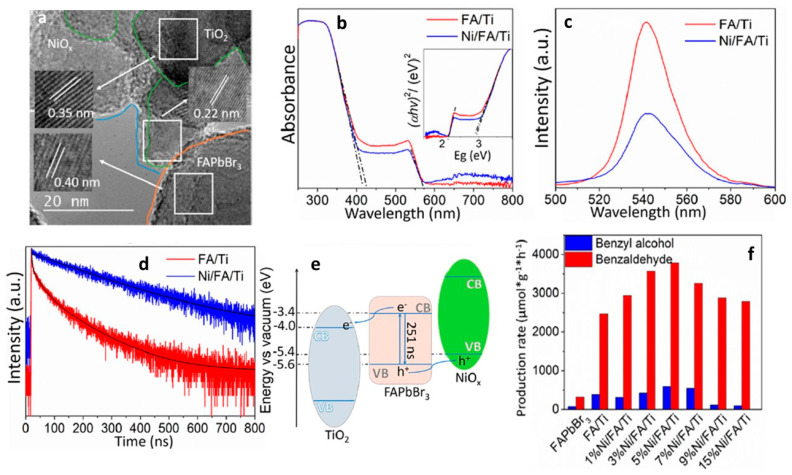
(**a**) HRTEM images of NiO_x_/FAPbBr_3_/TiO_2_ composite; (**b**) UV–vis DRS spectra with the corresponding bandgaps shown in the inset; (**c**) steady-state PL spectra and (**d**) PL decay spectra of the prepared FAPbBr_3_/TiO_2_ and 5% NiO_x_/FAPbBr_3_/TiO_2_; (**e**) experimentally supported mechanistic energy diagram of photoinduced charge transfer in NiO_x_/FAPbBr_3_/TiO_2_; (**f**) Investigation of the photocatalytic oxidation of toluene using pure FAPbBr_3_, FAPbBr_3_/TiO_2_, and a series of NiO_x_/FAPbBr_3_/TiO_2_ composites. Reproduced with permission. Copyright: 2019, American Chemical Society.

To enhance the spatial separation of electron and hole pairs, Roeffaers et al. designed a solar photocatalyst cell with the NiO_x_/FAPbBr_3_/TiO_2_ configuration, as shown in Figure 26a, for photocatalytic toluene oxidation via Csp^3^-H bond activation [97]. In this sandwich structure, NiO_x_ and TiO_2_ serve as hole and electron extracting layers, respectively. NiO_x_/FAPbBr_3_/TiO_2_ improved the photoinduced charge extraction compared to FAPbBr_3_/TiO_2_, as shown by the reduction in PL intensity in Figure 26c. Moreover, 2D NiO_x_ have great charge transmission capacities, thus improving the lifetime of the free charge carrier (Figure 26d,e). Figure 26f shows the photocatalytic oxidation of the C (sp^3^)-H of toluene to benzaldehyde and benzyl alcohol with a conversion rate of 320 and 54 µmol·g^−1^·h^−1^ for FAPbBr_3_ NPs and enhanced by eight-fold upon loading 15 wt% TiO_2_ under saturated O_2_ and simulated solar light (AM1.5 G). In addition to this, the loading of 5 wt% NiO_x_ on 15% FAPbBr_3_/TiO_2_ led to further enhancing the production rate by twelve-fold up to 3800 µmol·g^−1^·h^−1^ benzaldehyde with an 86% selectivity. A ten-fold decrease in the photocatalytic activity was observed when visible light was used for photoirradiation. This suggests that UV photons can be harvested by TiO_2_ to generate an exciton. NiO_x_/FAPbBr_3_/TiO_2_ photocatalyst also worked well for the oxidation of substituted toluene. It is noteworthy that cycloalkanes can be oxidised to their corresponding ketones with high selectivity (>99%).

Overall, the three component photocatalyst highlights the importance of designing a solar photocatalyst cell for strong absorption, charge separation, and transport. The mechanism of the formation of benzaldehyde is as follows: charge carriers are generated upon the photoexcitation of FAPbBr_3_, and electrons and holes are efficiently separated by TiO_2_ and NiO_x_, respectively. Moreover, molecular oxygen is reduced to a superoxide radical by interacting with electrons on TiO_2_, and the holes in the NiO_x_ reacted with toluene to form a toluene radical; this is the key step for the oxidation of C(sp^3^)-H bonds. Subsequently, toluene radicals would react with superoxide radicals to form the respective aldehyde. The formation of benzyl alcohol can be explained by the direct reaction between toluene radicals and dissolved free molecular oxygen in toluene. Furthermore, benzyl alcohol can be oxidised to benzaldehyde.

**Figure 27:**
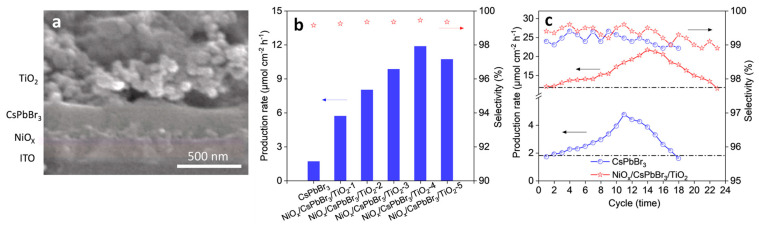
(**a**) Cross-sectional SEM of CsPbBr_3_-based solar photocatalyst cell; (**b**) selective photocatalytic oxidation of benzyl alcohol to benzaldehyde over films of CsPbBr_3_ and NiO_x_/CsPbBr_3_/TiO_2_ photocatalysts, where NiO_x_/CsPbBr_3_/TiO_2_-n (*n* = 1, 2, 3, 4, and 5) denotes various concentration of CsPbBr_3_ precursor solution: 0.05 M, 0.15 M, 0.25 M, 0.35 M, and 0.45 M, respectively, red color stars represents the selectivity of the photocatalysts; (**c**) assessment of the recycling test of NiO_x_/CsPbBr_3_/TiO_2_ solar photocatalyst cell and pure CsPbBr_3_ thin film photocatalyst. Reproduced with permission. Copyright: 2022, Elsevier.

Roeffaers and co-workers also employed the type-II heterojunction of NiO_x_/CsPbBr_3_/TiO_2_ in the form of planar architecture by making a thin film consisting of consecutive layers of NiO_x_, CsPbBr_3_, and TiO_2_ on top of tin-doped indium oxide (ITO) glass, as shown in the cross-section SEM image in Figure 27a. The main advantage of this planar architecture is that it improves the charge separation by supressing the recombination of excitons and protects the perovskite from the environment. In this type of catalyst, unlike solar cells, discontinuities in the thin film act as active sites. Thus, it is vital to establish a balance between a sufficient adsorption area and efficient charge separation. Intriguingly, the optimal NiO_x_/CsPbBr_3_/TiO_2_ photocatalyst exhibited 12.1 µmol·cm^−2^·h^−1^ of benzaldehyde formation from benzyl alcohol compared to CsPbBr_3_ films (1.1 µmol·cm^−2^·h^−1^), as shown in Figure 27b, and 8.7 µmol·cm^−2^·h^−1^ of benzaldehyde formation from toluene compared to CsPbBr_3_ films [127]. Moreover, the stability of the solar photocatalyst cell was evaluated using a recycling test of the cells for 23 successive cycles, where each cycle took 4 h; the results are shown in Figure 27c. Interestingly, an increase in photoactivity was observed with an increase in the reaction time for both systems. They achieved an average production rate of 16.3 µmol·cm^−2^·h^−1^ of benzaldehyde with a 99% selectivity after 90 h of testing with the NiO_x_/CsPbBr_3_/TiO_2_ photocatalyst, whereas CsPbBr_3_ films exhibit 4.8 µmol·cm^−2^·h^−1^ for 11 cycles of 4 h testing. In addition, the authors achieved an impressive TON of 45,300 for the first 23 cycles when using a NiO_x_/CsPbBr_3_/TiO_2_ photocatalyst. In contrast with many reports, where, beyond 2–3 cycles, a gradual decrease in the production rate of benzaldehyde was observed, in this case, the gradual increase in performance with the number of cycles was observed. This was attributed to improving the crystal symmetry and thereby increasing the mobility of charge carriers by trace amounts of water generated during the reaction. However, it has been inferred that this approach may not apply for the photocatalysts exhibiting a high conversion rate of benzaldehyde (ranging from a few hundred to several thousand µmol·g^−1^·h^−1^due to increase water generation, which can result in the perovskite deterioration.

Furthermore, this planar junction photocatalyst was also successful in oxidising other alcohols, such as 1-phenylethanol, 2-phenyl ethanol, and 4-fluorobenzylalcohol, thus confirming the general applicability of the solar photocatalyst cell.

Most recently, Yi et al. developed hierarchical CsPbBr_3_/TiO_2_ heterostructures by assembling CsPbBr_3_ NPs onto a nanoflower-shaped TiO_2_, which allowed for an intimate interfacial contact between CsPbBr_3_ NPs and TiO_2_ that greatly enhanced the toluene photocatalytic oxidation conversion [128]. This hierarchical heterojunction not only benefited from efficient charge separation due to its type-II band alignment but also offered a large surface area, allowed for increased absorption of light due to refraction and scattering, and enhanced toluene adsorption. As a result, the optimized CsPbBr_3_/TiO_2_ nanoflower-shaped photocatalyst demonstrated high performance, yielding a toluene conversion rate into benzaldehyde and benzyl alcohol of 8670 and 1530 µmol·g^−1^·h^−1^, respectively. This level of photoactivity surpasses that of pristine CsPbBr_3_ by 20-fold. This study highlights the critical role of TiO_2_ nanoflake morphology in elevating the overall performance of the photocatalyst.

## 11. Preparation of Singlet Oxygen Driven Oxidized Compounds Using g-C_3_N_4_/Perovskite

Singlet oxygen (^1^O_2_) is a highly reactive, short-lived species, valued for its role in facilitating [4 + 2] and [2 + 2] cycloaddition reactions as well as ene-reactions and epoxidations [129,130]. The generation of ^1^O_2_ through photochemical means is considered an environmentally friendly approach compared to chemical methods [131].

**Figure 28:**
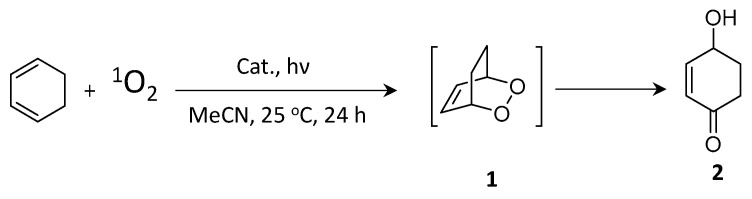
Hetero Diels–Alder (HDA) reaction of 1,3-cyclohexadiene and ^1^O_2_ using heterostructures g-C_3_N_4_ in combination with PEA_2_MX_4_.

Quadrelli et al. demonstrated the photochemical generation of ^1^O_2_ using oxidized g-C_3_N_4_ and its application in cycloaddition reactions [132]. Furthermore, recognizing the synergetic potential of heterostructures comprising metal halide perovskites and 2D materials for ^1^O_2_ generation, Corti et al. explored heterostructures involving g-C_3_N_4_ in combination with PEA_2_MX_4_ (PEA = phenylethylammonium; M = Pb and Sn; X = Br and Cl) and g-C_3_N_4_ in conjunction with DMASnBr_3_ (DMA = dimethylammonium). These heterostructures were employed in hetero Diels–Alder (HDA) as a benchmark reaction, employing 1,3-cyclohexadiene and ^1^O_2_ under the reaction conditions detailed in Figure 28, thereby leading to the formation of 4-hydroxycyclohex-2-en-1-one (2) (in Figure 28) [133]. Using g-C_3_N_4_/PEA_2_MX_4_ photocatalysts yielded moderate to good product yields, while DMASnBr_3_ reached yields of up to 63%. Additionally, various other reactions, including ene reactions and oxidations, have also been investigated. Nevertheless, certain limitations were observed in acyclic alkene oxidation, and poor chemo selectivity was noted in some cases.

## 12. Opening of Epoxide Reactions Using Lead-Free Cs_3_Bi_2_Br_9_ Perovskites

**Figure 29:**
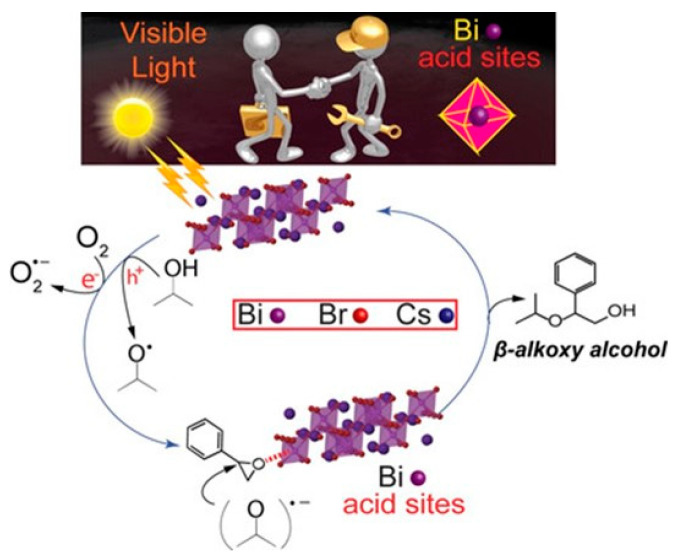
Proposed photocatalytic cycle for epoxide alcoholysis reaction using Cs_3_Bi_2_Br_9_ with proper Lewis acid sites on surface. Reproduced with permission. Copyright: 2019, John Wiley and Sons.

Dai et al. successfully demonstrated the use of lead-free Cs_3_Bi_2_Br_9_ perovskite in ring-opening reactions of epoxides to produce β-alkoxy alcohols [98]. This approach offers an alternative to the conventional method for the preparation of β-alkoxy alcohols, which often involves harsh conditions with corrosive, strong acids and high energy requirements. The Cs_3_Bi_2_Br_9_ perovskite was prepared in situ by adding a precursor solution of CsBr and BiBr_3_ in DMSO directly into the reaction medium containing the epoxide and alcohol. The alcohol acted as an antisolvent and nucleophile. Microscopy and a powder X-ray diffraction analysis revealed that larger aggregates of Cs_3_Bi_2_Br_9_ perovskite formed in the antisolvent method. The resulting Cs_3_Bi_2_Br_9_ perovskite was used as a photocatalyst for the synthesis of 2-isopropoxy-2-phenylethanol from styrene oxide and isopropanol (Figure 29). Remarkably, this reaction exhibited an activity of 1333 µmol h^−1^ g^−1^, a product yield of up to 86%, and an excellent selectivity (>99%) after 6 h irradiation under visible light. This facile photocatalytic system produced only the β-alkoxy alcohol regioisomer. The specific reaction conditions can be observed in the provided scheme.

In contrast to this, when the same reaction was carried out using CsPbBr_3_, BiBr_3_, CsBr, and Bi_2_O_3_ photocatalysts, a poor photocatalytic activity was observed, specifically a conversion rate of 1%, 13%, <1%, and 9% for CsPbBr_3_, BiBr_3_, CsBr, and Bi_2_O_3_, respectively. This is indicative of active sites related to Bi atoms for epoxide alcoholysis. Other type of ring opening of epoxides showed that the conversion rates were dependent on the electron density, steric hindrance of epoxide, and polarity of the solvent. An investigation on the epoxide opening mechanism suggests that weak Lewis-acid sites existing on the Cs_3_Bi_2_Br_9_ surface helps the product formation, which is in line with strong acidic conditions used for the ring opening of epoxides in the case of a thermal catalytic approach. The photocatalytic mechanism is shown in the Figure 29. The photoexcitation of the perovskite generates electrons and holes in the CB and VB, respectively, and molecular oxygen forms an oxygen radical anion by interacting with the excited electrons. The photogenerated holes interacting with alcohols result in the formation of an alcohol radical, which acts as an active nucleophile, while the Bi-based Lewis sites on the photocatalyst activate the epoxide by coordinating with the oxygen atom of the three membered heterocyclic ring. Finally, the attack of the nucleophile on the activated epoxide from the rear side results in the formation of the desired product.

## 13. Benzaldehyde Formation by Toluene Oxidation

### 13.1. Lead-Free Cs_3_Bi_2_Br_9_ Perovskites

**Figure 30:**
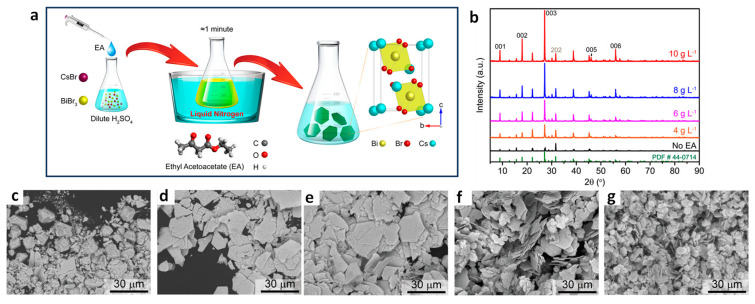
(**a**) The synthesis procedure for Cs_3_Bi_2_Br_9_ platelet crystals; (**b**) X-ray diffraction patterns of the Cs_3_Bi_2_Br_9_ microcrystals; (**c**) scanning electron microscopy (SEM) image of the irregular crystals grown without the addition of EA; (**c**–**g**) SEM images displaying platelet-shaped crystals grown in the presence of EA with different perovskite concentrations of 10, 8, 6, and 4 g L^−1^, respectively. Reproduced with permission. Copyright: 2021, John Wiley and Sons.

Dail et al. used lead-free Cs_3_Bi_2_Br_9_ platelets for toluene oxidation [134]. They prepared Cs_3_Bi_2_Br_9_ platelets via the rapid cooling of the precursor solution in dilute H_2_SO_4_ in the presence of ethyl acetoacetate (EA) under liquid nitrogen conditions (Figure 30a) [134]. The absence of EA resulted in irregularly shaped platelets with a wide size range (Figure 30a). However, the inclusion of EA at varying concentrations of halide perovskite in the dilute H_2_SO_4_ solution (ranging from 10 to 4 g/L) led to the formation of platelets with controlled dimensions. The basal plane size of the platelets decreased from 40 µm to 2–10 µm, while the thickness ranged from 500 to 100 nm. The presence of EA was found to induce the preferential growth of Cs_3_Bi_2_Br_9_ platelets along the (003) planes, as confirmed by powder X-ray diffraction (XRD) (Figure 30b) and a scanning electron microscopy (SEM) analysis (Figure 30c–g). This control over crystal growth was attributed to the strong interaction between the carbonyl groups of EA and Bi atoms.

Thinner Cs_3_Bi_2_Br_9_ platelets were employed as a photocatalyst for the C–H bond activation of toluene under open-air conditions and converted 232 µmol of toluene in 36 h of light irradiation. The photocatalytic oxidation of toluene resulted in the formation of benzaldehyde with a yield of 2.2% and a selectivity of at least 88%. As byproducts, benzaldehyde and benzoic acid were also observed. The interaction of photogenerated holes with toluene would result in the formation of benzyl radicals, followed by a reaction with superoxide radicals, which is formed via the interaction of molecular oxygen with excited electrons, such as the oxidation of toluene by the TiO_2_/FAPbBr_3_/NiOx composite.

### 13.2. Cs_3_Bi_2_Br_9_/Pd Heterostructures

Roeffaers and co-workers reported the development of Schottky junction-type heterostructures involving lead-free Cs_3_Bi_2_Br_9_ and Pd for benzyl alcohol oxidation and H_2_ generation [135,136]. They achieved this by using crystalline Cs_3_Bi_2_Br_9_ as the core material, complemented by amorphous Pd nanocubes forming the outer shell. Cs_3_Bi_2_Br_9_/Pd Schottky junction exhibited a four-fold enhancement in performance compared to pure Cs_3_Bi_2_Br_9_, boosting benzaldehyde formation with >99% selectivity and H_2_ production rates from 368 ± 30 and 341 µmol·g^−1^·h^−1^ to 1457 ± 78 and 1421 ± 91 µmol·g^−1^·h^−1^, respectively.

### 13.3. Cs_3_Bi_2_Br_9_/TiO_2_ Heterostructures

**Figure 31:**
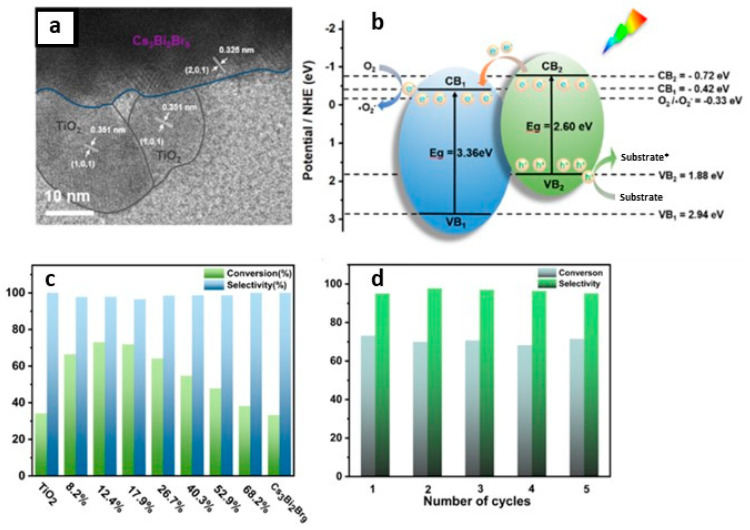
(**a**) HRTEM of 12.4% Cs_3_Bi_2_Br_9_/TiO2; (**b**) band structures of TiO_2_, Cs_3_Bi_2_Br_9_ and photoinduced charge carrier transfer under illumination. VB is valence band, and CB is conduction band. Substrate * represents the hole transfer to the substrate; (**c**) photocatalytic selective oxidation of benzyl alcohol over X% Cs_3_Bi_2_Br_9_/TiO_2_; (**d**) recycle test of photocatalytic oxidation of benzyl alcohol over 12.4% Cs_3_Bi_2_Br_9_/TiO_2_. Reproduced with permission. Copyright: 2022, Elsevier.

The lower conversion efficiency of toluene oxidation using Cs_3_Bi_2_Br_9_ platelets would be attributed to an electron and hole recombination before reaching the surface of the platelets, owing to their long distances. Lu and co-workers employed type-II heterojunctions of lead-free layered perovskite, i.e., Cs_3_Bi_2_Br_9_ and TiO_2_ for oxidation of benzyl alcohol to benzaldehyde [137]. Figure 31a shows the HRTEM image of the Cs_3_Bi_2_Br_9_/TiO_2_ heterostructure. The band alignment in Figure 31b shows the type-II heterojunction enables the effective separation of photogenerated electrons and supresses the electron–hole recombination. Figure 31c shows the conversion rate of benzyl alcohol to benzaldehyde, where Cs_3_Bi_2_Br_9_ showed a 33.2% yield, while an optimal Cs_3_Bi_2_Br_9_/TiO_2_ heterojunction shows a 73% conversion with near 100% selectivity and 1465 µmol·g^−1^·h^−1^ photocatalytic activity. Interestingly, the recycle test shows a similar conversion rate at up to five cycles. It is noted that the conversion rate of pristine lead-free Cs_3_Bi_2_Br_9_ perovskite shows ~5 times higher performance than LHPs. This could be attributed to the more positive VBM of lead-free Cs_3_Bi_2_Br_9_ perovskite than LHPs, which provides a strong driving force for the hole transfer and activates the oxidation of benzyl alcohol.

### 13.4. Cs_3_Bi_2_Br_9_/MXene Heterostructures

MXenes are derived from MAX phases with a general formula M_n+1_AX_n_, where *n* = 1–3, M is a transition metal, A is mostly a group 13 or 14 element, and X is carbon and/or nitrogen; Tx represents surface terminations [138]. These two-dimensional (2D) nanomaterials have garnered significant attention due to their excellent properties, such as high metallic electrical conductivity, mobility, and excellent hydrophobicity [139]. Moreover, its layered nature and abundant surface terminations offer excellent compatibility to construct heterojunctions [140].

**Figure 32:**
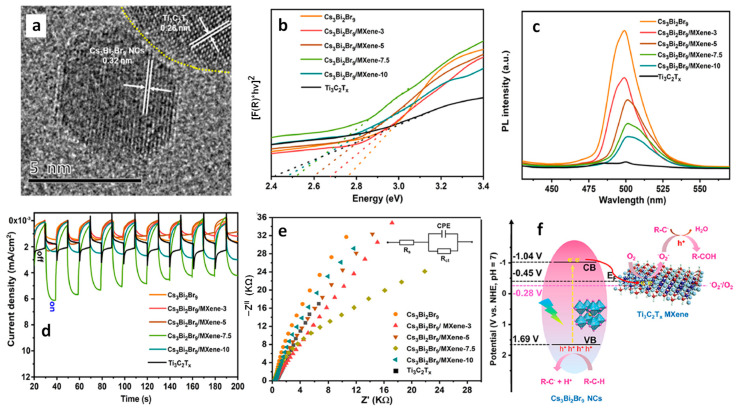
(**a**) High-resolution-TEM image Cs_3_Bi_2_Br_9_/MXene-7.5; (**b**) bandgap profile, (**c**) PL spectra (excitation at 380 nm); (**d**) transient photocurrent spectra and (**e**) electrochemical impedance spectroscopy (EIS) Nyquist plots of Cs3Bi2Br9 NCs, Cs_3_Bi_2_Br_9_/MXene-3, Cs_3_Bi_2_Br_9_/MXene-5, Cs_3_Bi_2_Br_9_/MXene-7.5, Cs_3_Bi_2_Br_9_/MXene-10, and Ti_3_C_2_T_x_; (**f**) Schematic representation of the separation of photoinduced carriers of the Cs_3_Bi_2_Br_9_/MXene heterostructure and the proposed mechanism for the photocatalytic reaction of alkane. Reproduced with permission. Copyright: 2021, American Chemical Society.

Yang and co-workers reported the type-II heterojunction of between Cs_3_Bi_2_Br_9_ NCs and Ti_3_C_2_T_x_ MXene for a toluene oxidation reaction [141]. Figure 32a shows the HRTEM image of Cs_3_Bi_2_Br_9_/Ti_3_C_2_T_x_ composite showing the highly crystalline nature of MXene and Cs_3_Bi_2_Br_9_. The bandgaps are calculated from the Tauc plots. The narrowing of the bandgap from 2.75 eV for pristine Cs_3_Bi_2_Br_9_ NCs to 2.47 eV for Cs_3_Bi_2_Br_9_/MXene-10 suggests the absorption of more visible light due to MXene’s smaller bandgap than that of Cs_3_Bi_2_Br_9_ (Figure 32b). Furthermore, steady-state PL shows a significant decrease in PL intensity upon increasing in the loading of MXene on Cs_3_Bi_2_Br_9_, indicative of the suppression of electron–hole recombination (Figure 32c). In addition, transient photocurrent spectra show the production of a high photocurrent (Figure 32d), and EIS Nyquist plots show less interfacial charge resistance (Figure 32e) for Cs_3_Bi_2_Br_9_/MXene-7.5 and suggests the appropriate composition for the photocatalyst. Cs_3_Bi_2_Br_9_/MXene-7.5 exhibits a higher photocatalytic toluene conversion than pristine Cs_3_Bi_2_Br_9_ NCs (2121 µmol·g^−1^·h^−1^ vs. 850 µmol·g^−1^·h^−1^) and with 100% selectivity to benzaldehyde under blue LED (5 W) illumination.

Interestingly, this performance was enhanced by almost two times under Xe lamp (300 W) without changing the selectivity. Furthermore, these catalysts can also be used for oxidizing electron-donating and -withdrawing group substituents. The impressive performance of the Cs_3_Bi_2_Br_9_/MXene-7.5 composite is due to the efficient separation of photoinduced electrons, allowed by the excellent electrical conductivity of the monolayer Ti_3_C_2_T_x_ MXene and the intimate Cs_3_Bi_2_Br_9_/MXene contact. Furthermore, the ultra-thin nature of 2D MXenes reduces the migration distance of charge carriers to the MXene surface, thus contributing to its enhanced capabilities.

### 13.5. Cs_3_Bi_2_Br_9_/g-C_3_N_4_ Heterostructures

Graphite-like carbon nitride, g-C_3_N_4_, is a metal-free n-type semiconductor and boasts distinct optical, electrical, structural, and physiochemical features [142]. These attributes establish g-C_3_N_4_ as an innovative material with a wide range of applications in multifunctional nanoplatforms, particularly in electronics, catalysis, and energy [143,144,145]. Its versatility is evident in its ability to create tailored hybrid photocatalysts with diverse compositions and structures, capturing interest for their effectiveness in solar radiation absorption and charge carrier separation, making g-C_3_N_4_ a key player in advancing photocatalysis.

Bai et al. designed a type-II heterojunction employing nanosheets of Cs_3_Bi_2_Br_9_ and g-C_3_N_4_. This heterojunction between nanosheets of amorphous g-C_3_N_4_ and crystalline Cs_3_Bi_2_Br_9_ allows for a large area interface sharing between Cs_3_Bi_2_Br_9_ and g-C_3_N_4_. Consequently, the lifetime of photogenerated charge carriers in Cs_3_Bi_2_Br_9_ perovskite will be enhanced by suppressing the electron–hole recombination [146]. The band offset between Cs_3_Bi_2_Br_9_ and g-C_3_N_4_ facilitates the transfer of photoinduced holes from Cs_3_Bi_2_Br_9_ to g-C_3_N_4_ and electrons from g-C_3_N_4_ to Cs_3_Bi_2_Br_9_ in the type-II heterojunction of Cs_3_Bi_2_Br_9_/g-C_3_N_4_.

The photocatalytic properties of the prepared catalysts were assessed for the selective oxidation of toluene under visible light illumination. The optimized photocatalyst 10% Cs_3_Bi_2_Br_9_/g-C_3_N_4_ composite demonstrated the highest benzaldehyde formation rate of 4.53 mmol·h^−1^·g^−1^, which is 41.8 and 2.3 times higher than g-C_3_N_4_ and Cs_3_Bi_2_Br_9_ nanosheets, respectively. Moreover, the selectivity of this composite was approximately 90%.

### 13.6. Cs_3_Bi_2_Br_9_/CdS Heterostructures

**Figure 33:**
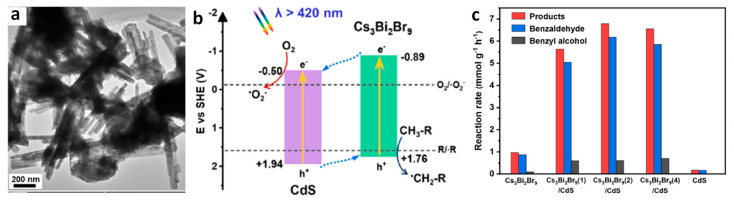
(**a**) TEM image of Cs_3_Bi_2_Br_9_/CdS hybrid; (**b**) band alignment of Cs_3_Bi_2_Br_9_ and CdS, and the proposed transfer pathways for photogenerated carriers and redox reactions under visible light (λ > 420 nm); (**c**) Photocatalytic activities of toluene oxidation using Cs_3_Bi_2_Br_9_/CdS, Cs_3_Bi_2_Br_9_, and CdS as photocatalysts. Reproduced with permission. Copyright: 2023, Elsevier.

Zou et al. prepared type-II heterojunction of Cs_3_Bi_2_Br_9_/CdS by the in situ growth of Cs_3_Bi_2_Br_9_ NPs on the preformed CdS nanorods as shown in Figure 33a [147]. The electronic band alignment of the Cs_3_Bi_2_Br_9_/CdS composite shows a type-II band structure (Figure 33b), allowing for photoinduced holes to accumulate in the VB of Cs_3_Bi_2_Br_9_ and electrons in the CB of CdS lead to the efficient photooxidation of toluene. Figure 33c shows the conversion rate of toluene for the pristine Cs_3_Bi_2_Br_9_ and Cs_3_Bi_2_Br_9_/CdS composites. As expected, the composite exhibits an excellent performance of 6.79 mmol·g^−1^·h^−1^, which is higher than 37.6- and 7.1-fold CdS and Cs_3_Bi_2_Br_9_, respectively. Furthermore, DFT studies suggests that the interaction between C/O atoms of intermediates (benzyl radical) and Cs^+^ of Cs_3_Bi_2_Br_9_ lead to C–C or O–C bonds. This indicates that Cs^+^ also play a key role in the surface activation of aromatic C–H bonds.

### 13.7. Cs_3_Sb_2_Br_9_/g-C_3_N_4_ Heterostructures

**Figure 34:**
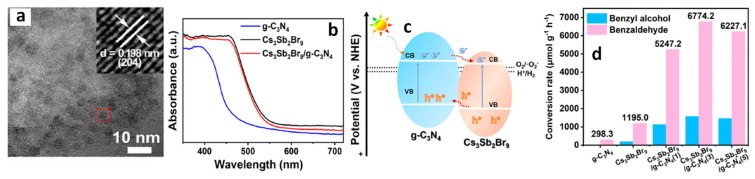
(**a**) TEM images of Cs_3_Sb_2_Br_9_/g-C_3_N_4_, inset shows the high-resolution image of single particle of the NC highlighted in the TEM image; (**b**) UV-visible absorption spectra of g-C_3_N_4_, Cs_3_Sb_2_Br_9_, and Cs_3_Sb_2_Br_9_/g-C_3_N_4_; (**c**) schematic illustration of the band position of Cs_3_Sb_2_Br_9_ and g-C_3_N_4_ and the proposed transfer pathways for photogenerated carriers; (**d**) photocatalytic oxidation of toluene conversion rate over diverse photocatalysts. Reproduced with permission. Copyright: 2021, John Wiley and Sons.

Kuang et al. demonstrated the type-II heterojunction of Cs_3_Sb_2_Br_9_/C_3_N_4_ by in situ growth of crystalline Cs_3_Sb_2_Br_9_ NPs (2–6 nm) on the g-C_3_N_4_ amorphous nanosheets, as shown in Figure 34a [148]. Figure 34b shows the absorption spectrum of Cs_3_Sb_2_Br_9_/C_3_N_4_ composite and their individual components, suggesting more visible light absorption of Cs_3_Sb_2_Br_9_/C_3_N_4_ composite due to Cs_3_Sb_2_Br_9_. Electronic band structure calculation shows that the interface of Cs_3_Sb_2_Br_9_/g-C_3_N_4_ composite is a type-II heterojunction as shown Figure 34c. Type-II band structure allows for the accumulation of photoinduced holes in the CB band of g-C_3_N_4_, which activate toluene to generate benzyl radical intermediates, while photoinduced electrons move to the CB of Cs_3_Sb_2_Br_9_ and reduce O_2_ to O_2_^−^. This efficient charge transfer due to the strong absorption of light results in high photocatalytic activity, specifically of 8346.8 µmol·g^−1^·h^−1^ for toluene oxidation, i.e., 26.6- and 6.0-fold higher than g-C_3_N_4_ and Cs_3_Bi_2_Br_9_, respectively (Figure 34d).

### 13.8. Z-Scheme Heterojunction Formation by Cosharing Atoms

**Figure 35:**
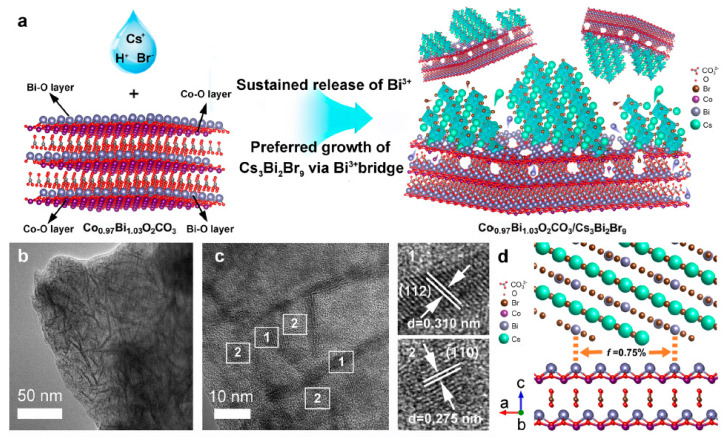
(**a**) The formation schematic diagram of Co_0_._97_Bi_1_._03_O_2_CO_3_/Cs_3_Bi_2_Br_9_ heterojunction; (**b**) TEM; (**c**) HRTEM images, box 1 and 2 represent ridge-like tiny nanosheets of and Cs_3_Bi_2_Br_9_ and Co_0_._97_Bi_1_._03_O_2_CO_3_ nanosheets, respectively; (**d**) the lattice match of Co_0_._97_Bi_1_._03_O_2_CO_3_/Cs_3_Bi_2_Br_9_ hybrid; Reproduced with permission. Copyright: 2023, Elsevier.

Teng et al. demonstrated the 2D/2D heterojunction of Co_x_Bi_2−x_O_2_CO_3_/Cs_3_Bi_2_Br_9_ (x = ~1) in the form of nanosheets [149]. The heterojunction was prepared by in situ acid etching strategy by taking Co_x_Bi_2−x_O_2_CO_3_ as the precursor and fundamental constituent of Bi^3+^. The Bi atoms of Co_x_Bi_2−x_O_2_CO_3_ nanosheet serve as the nucleation sites to grow the Cs_3_Bi_2_Br_9_ perovskite, as shown in Figure 35a. The TEM image in Figure 35b shows the ridge-like tiny nanosheets with a thickness of ~2 nm that emerged on the surface of Co_0_._97_Bi_1_._03_O_2_CO_3_; labels 1 and 2 in Figure 35c correspond to the Cs_3_Bi_2_Br_9_ and Co_0_._97_Bi_1_._03_O_2_CO_3_, respectively. It was found that the (112) plane of Cs_3_Bi_2_Br_9_ with a Bi-Bi distance of 14.2 Å is similar to the distance between five Bi atoms in (002) face of Co_0_._97_Bi_1_._03_O_2_CO_3_ with a small lattice mismatch (<0.75%). This led to the epitaxial growth of Cs_3_Bi_2_Br_9_ in the (001) face direction, forming an angle of 51.06° with the layer of Co_0_._97_Bi_1_._03_O_2_CO_3_, as shown in Figure 35d.

**Figure 36:**
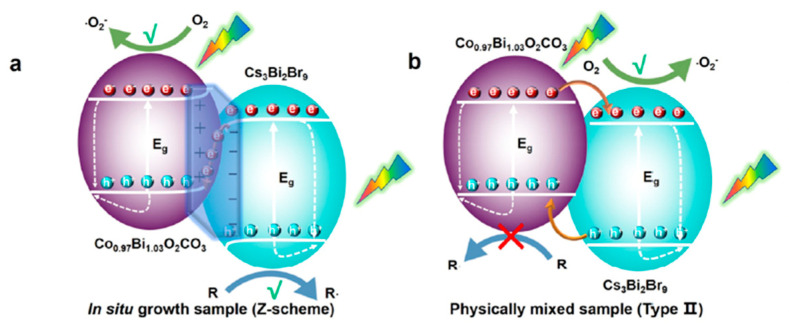
The photoinduced carriers transfer process of (**a**) in situ grown Co_0_._97_Bi_1_._03_O_2_CO_3_/Cs_3_Bi_2_Br_9_ heterojunction; (**b**) physically mixed Co_0_._97_Bi_1_._03_O_2_CO_3_/Cs_3_Bi_2_Br_9_ sample. Reproduced with permission. Copyright: 2023, Elsevier.

Experimental investigations and DFT analyses indicate the establishment of a staggered band structure, where the Fermi level of Co_0_._97_Bi_1_._03_O_2_CO_3_ was observed to be higher than that of Cs_3_Bi_2_Br_9_. Fermi level alignment occurs when these two materials come into close contact, thus leading to a redistribution of charges. This phenomenon induces band bending and an interfacial built-in electric field (IEF) in the direction of Co_0_._97_Bi_1_._03_O_2_CO_3_ to Cs_3_Bi_2_Br_9_ (Figure 36a). Furthermore, a Bader charge as high as 0.977 e was observed and corresponded to the electron transfer from Co_0_._97_Bi_1_._03_O_2_CO_3_ to Cs_3_Bi_2_Br_9_, indicative of a strong interfacial electronic coupling interaction, which will contribute to a large IEF. This kind of heterointerface with a large IEF can guide the photoinduced electrons and holes transporting via the Z-scheme route rather than type-II heterojunction. To investigate the type of heterojunction of between Co_x_Bi_2−x_O_2_CO_3_ and Cs_3_Bi_2_Br_9_, control studies were conducted with DMPO to capture ·OH species. The CBM of both Co_0_._97_Bi_1_._03_O_2_CO_3_ and Cs_3_Bi_2_Br_9_ can drive the production of O_2_^−^. In addition, the VBM of Co_0_._97_Bi_1_._03_O_2_CO_3_ is too negative to form OH radicals. Interestingly, the Co_0_._97_Bi_1_._03_O_2_CO_3_/Cs_3_Bi_2_Br_9_ composite showed strong peaks of OH radicals, whereas a physical mixture of Co_0_._97_Bi_1_._03_O_2_CO_3_ and Cs_3_Bi_2_Br_9_ failed to show OH radical formation. This suggests the Co_0_._97_Bi_1_._03_O_2_CO_3_/Cs_3_Bi_2_Br_9_ composite exhibits a Z-scheme-type heterojunction (Figure 36a), and their physical mixture shows type-II band alignment (Figure 36b).

To check the charge separation efficiency, the photocatalytic activity of toluene oxidation under visible light illumination was chosen because the VBM of Co_0_._97_Bi_1_._03_O_2_CO_3_ is not positive enough to form the benzyl radical from toluene. Notably, Co_0_._97_Bi_1_._03_O_2_CO_3_ and the physical mixture of Co_0_._97_Bi_1_._03_O_2_CO_3_ and Cs_3_Bi_2_Br_9_ show no activity. The Co_0_._97_Bi_1_._03_O_2_CO_3_/Cs_3_Bi_2_Br_9_ composite and Cs_3_Bi_2_Br_9_ showed an activity of benzaldehyde formation of 1837 and ~883 µmol·g^−1^·h^−1^. A physical mixture shows no activity due to photoinduced holes of Cs_3_Bi_2_Br_9_ that accumulate in the VB of Co_0_._97_Bi_1_._03_O_2_CO_3_, which cannot drive the toluene oxidation due to an unfavourable band alignment. These results further highlight the pivotal role of high-quality interfaces with an atomic-level close contact of Co_0_._97_Bi_1_._03_O_2_CO_3_/Cs_3_Bi_2_Br_9_ heterojunction.

### 13.9. Z-Scheme Heterojunction of Cs_3_Bi_2_Br_9_/d-BiOBr

**Figure 37:**
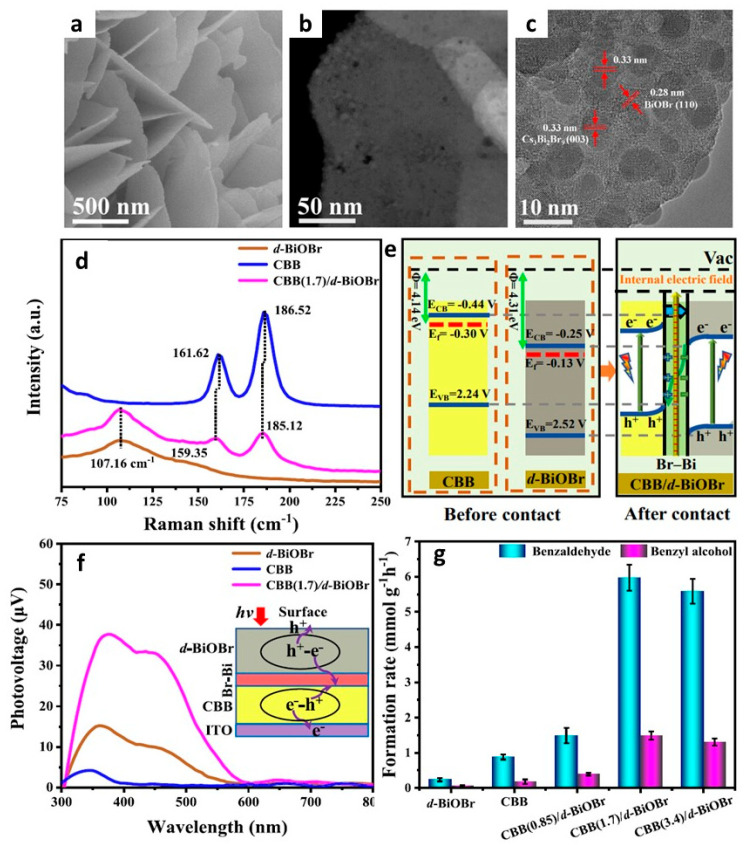
(**a**) SEM and (**b**) TEM images of d-BiOBr. (**c**) HRTEM images of CBB(1.7)/d-BiOBr. (**d**) Raman spectra of d-BiOBr, CBB, and CBB(1.7)/d-BiOBr; (**e**) represents the band alignment of CBB and d-BiOBr before and after contact; (**f**) SPV spectra; (**g**) comparison of photocatalytic toluene oxidation performance over d-BiOBr, CBB, and the CBB/d-BiOBr composite. Reproduced with permission. Copyright: 2022, American Chemical Society.

Bai et al. reported the construction of a Z-scheme heterojunction of Cs_3_Bi_2_Br_9_/d-BiOBr (d-defective) for the photocatalytic toluene oxidation [150]. Bi atoms in d-BiOBr serve as a source of Bi and nucleation sites for Cs_3_Bi_2_Br_9_ perovskite growth. Figure 37a,b show the SEM, TEM images of d-BiOBr nanosheets with a thickness of ~4.5 nm, and Figure 37c shows the HRTEM image of the Cs_3_Bi_2_Br_9_/d-BiOBr composite, indicating the epitaxial growth of Cs_3_Bi_2_Br_9_ nanodots with a particle size of ~12.1 nm on d-BiOBr ultrathin nanosheets. To know the interaction between Cs_3_Bi_2_Br_9_ and d-BiOBr, Raman measurements were conducted on the Cs_3_Bi_2_Br_9_, d-BiOBr, and Cs_3_Bi_2_Br_9_/d-BiOBr composite. The vibrational modes of BiBr_2_ (186.52 cm^−1^) and BiBr_3_ (161.62 cm^−1^) for the Cs_3_Bi_2_Br_9_/d-BiOBr composite are shifted towards a lower wave number, indicating the interaction between Br- of Cs_3_Bi_2_Br_9_ and [Bi_2_O_2_]^2+^ of d-BiOBr (Figure 37d). As a result, the Bi–Br bond stretched the [Bi_2_Br_9_]^3−^ cluster, which was evident from the increase of Bi–Br bond distance from 0.276 nm to 0.313 nm in the DFT calculations. Thereby, Bi–Br bond could function as an interfacial bridge between Cs_3_Bi_2_Br_9_ and d-BiOBr.

Band structure of d-BiOBr, Cs_3_Bi_2_Br_9_, Cs_3_Bi_2_Br_9_/d-BiOBr are shown in Figure 37e. The Fermi level (E_f_) of Cs_3_Bi_2_Br_9_, which is higher than that of d-BiOBr, leads to the diffusion of electrons from Cs_3_Bi_2_Br_9_ to d-BiOBr to establish a new equilibrium state. This results in generating a built-in electric field in the direction of Cs_3_Bi_2_Br_9_ to d-BiOBr at the interface. Further, steady-state surface photovoltage measurements showed a higher photovoltage for the Cs_3_Bi_2_Br_9_/d-BiOBr composite than the other two compositions at 300–520 nm. This implies that there is a migration of photoinduced holes in d-BiOBr to the illuminated side and migration of photoexcited electrons of Cs_3_Bi_2_Br_9_ to the non-illuminated side (Figure 37f). The remaining photogenerated electrons of d-BiOBr, recombining with the photoinduced holes of Cs_3_Bi_2_Br_9_ via Bi–Br interfacial bridge, led to efficient electron hole separation via Z-scheme route.

The photocatalytic performance of the catalysts was evaluated on the selective toluene oxidation under visible light illumination. The Cs_3_Bi_2_Br_9_(1.7)/d-BiOBr composite exhibited a conversion rate of 7.24 mmol·g^−1^·h^−1^, which is 24.1 and 6.8 times higher than that of d-BiOBr and Cs_3_Bi_2_Br_9_, respectively, and with a selectivity of 80% for benzaldehyde (Figure 37g). Furthermore, the Cs_3_Bi_2_Br_9_(1.7)/d-BiOBr composite showed high stability and repeatability of performance up to 5 cycles. In support with photocatalytic activity, the DFT calculation also suggests a lower E_ad_ and activation energy (−1.03 and 0.85 eV, respectively) for the Cs_3_Bi_2_Br_9_/d-BiOBr composite with two holes than d-BiOBr with one hole (−0.62 and 1.79). These results suggest that photogenerated holes transferred to the surface are higher in the Cs_3_Bi_2_Br_9_(1.7)/d-BiOBr composite than in d-BiOBr, thus demonstrating the pivotal role of a Z-scheme heterojunction in the photocatalytic activity.

Similarly, Roeffaers et al. constructed Z-scheme heterojunctions using a FAPbBr_3_/Bi_2_WO_6_ composite [151]. The VB position of Bi_2_WO_6_ is suitable for benzyl alcohol oxidation, whereas the CB position of FAPbBr_3_ is for CO_2_ reduction. FAPbBr_3_/Bi_2_WO_6_, 12.5%, exhibited high benzaldehyde formation (250 µmol·g^−1^·h^−1^), more than 16 and 4 times that of FAPbBr_3_ and BiWO_6_ under AM 1.5 G simulate solar light irradiation. In addition, the Z-scheme heterojunction approach was also used for the photooxidation of benzyl alcohol [152] and CO_2_ reduction [153,154].

### 13.10. Sb-Doped Cs_3_Bi_2_Br_9_ Perovskite

To enhance the absorption of visible light and activate catalytic sites of the Cs_3_Bi_2_Br_9_ nanosheets, Bai et al. doped the nanosheets with Sb for toluene oxidation [155]. They prepared various compositions of Cs_3_Bi_2−x_Sb_x_Br_9_ (x = 0, 0.2, 0.5, 1.3, and 2) nanosheets and observed that the bandgaps of x = 0.2, 0.5, 1.3 in Cs_3_Bi_2−x_Sb_x_Br_9_ were 2.40, 2.32, and 2.24 eV, respectively, which are smaller than those of Cs_3_Bi_2_Br_9_ (2.63 eV) and Cs_3_Sb_2_Br_9_ (2.45 eV). This phenomenon was attributed to the defect-related absorption resulting from the energy mismatch between Bi and Sb atomic orbitals in the Cs_3_Bi_2−x_Sb_x_Br_9_ perovskite [156].

**Figure 38:**
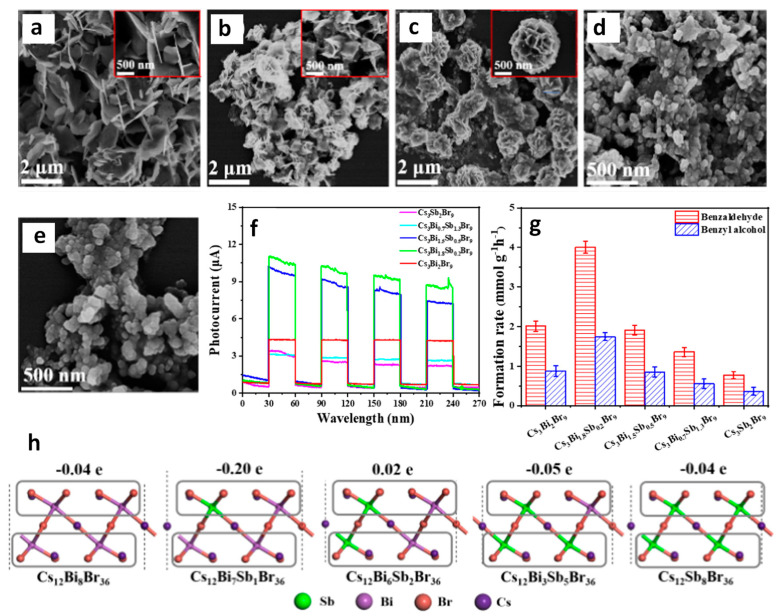
SEM image of (**a**) Cs_3_Bi_2_Br_9_, (**b**) Cs_3_Bi_1_._8_Sb_0_._2_Br_9,_ (**c**) Cs_3_Bi_1_._5_Sb_0_._5_Br_9_, (**d**) Cs_3_Bi_0_._7_Sb_1_._3_Br_9_, (**e**) Cs_3_Sb_2_Br_9_, inset in (**a**–**c**) show the magnified view of the SEM images; (**f**). Transient photocurrent response; (**g**) Photocatalytic performances of the prepared samples after irradiation of 3 h; (**h**) Bader electrons of upper layer minus that of lower layer over different models. Cs_12_Bi_8_Br_36_ is used as a model of Cs_3_Bi_2_Br_9_, Cs_12_Bi_8_Br_36_ were substituted by 0, 1, 2, 5, and 8 Sb atoms to simulate the experimental ratio of Sb doping. Reproduced with permission. Copyright: 2022, Elsevier.

SEM images for different compositions of Cs_3_Bi_2−x_Sb_x_Br_9_ revealed that Cs_3_Bi_2_Br_9_ exists in the form of nanosheets, and the morphology changed from nanosheets to NPs by increasing the amount of Sb (Figure 38a–e). Specifically for x = 0.2 and 0.5, a sheet-like structure was maintained, while for x = 1.3, NP formation was detected, resembling Cs_3_Sb_2_Br_9_.

Transient photocurrent measurements indicated that Cs_3_Bi_1_._8_Sb_0_._2_Br_9_ exhibited the highest photocurrent intensity, surpassing Cs_3_Bi_1_._5_Sb_0_._5_Br_9_, Cs_3_Bi_2_Br_9_, Cs_3_Bi_0_._7_Sb_1_._3_Br_9_, and Cs_3_Sb_2_Br_9_. This finding suggested that Cs_3_Bi_1_._8_Sb_0_._2_Br_9_ was the best composition in terms of charge carrier generation (Figure 38f). Furthermore, this behaviour was reflected in the photocatalytic activity of toluene conversion, with Cs_3_Bi_1_._8_Sb_0_._2_Br_9_ nanosheets displaying the highest toluene conversion rate of 5830 µmol h^−1^ g^−1^, which is 3.3 times higher than that of the NPs (Figure 38g) indicates the vital role of the high surface area with many active sites.

DFT studies were conducted to determine the effects of Sb doping on hole generation and, subsequently, the photocatalytic activity; DFT studies were conducted. A charge difference analysis for different compositions showed that Cs_12_Bi_7_Sb_1_Br_36_ had electrons of the layer 0.2 e lower than that of the other layer, indicating hole accumulation on the Sb side (Figure 38h). This suggested that when Sb doping was small, most of the Sb was located on one side, promoting hole generation. Consequently, Cs_3_Bi_1_._8_Sb_0_._2_Br_9_ nanosheets exhibited high activity due to extended absorption with efficient charge generation and separation [157].

Similar to this study, Cui et al. conducted a study on the impact of Sb-doping in Cs_3_BiBr_9_ for the photocatalytic oxidation of thioanisole, revealing that the strategic introduction of Sb in place of Bi resulted in a reduced bandgap and decreased Bi-related defects near the CBM, leading to enhanced carrier mobility [158]. Specifically, the composition Cs_3_Bi_2−x_Sb_x_Br_9_ (CBSB; x = 0.3) displayed outstanding performance in the photocatalytic oxidation of thioanisole to methyl phenyl sulfoxide in hexane. Notably, CBSB-0.3 demonstrated exceptional efficiency with a 95% conversion rate and 99% selectivity and maintained stability up to five cycles, outperforming all other compositions. In contrast, analogous studies using CsPbBr_3_ perovskite failed to demonstrate any noticeable activity, highlighting the promising potential of CBSB-0.3 perovskites in facilitating this oxidation reaction. Furthermore, control experiments established that the primary active species responsible for the formation of methyl phenyl sulfoxide were photogenerated holes and O_2^−^_.

### 13.11. Effect of A-Site Cation in A_3_Sb_2_Br_9_

**Figure 39:**
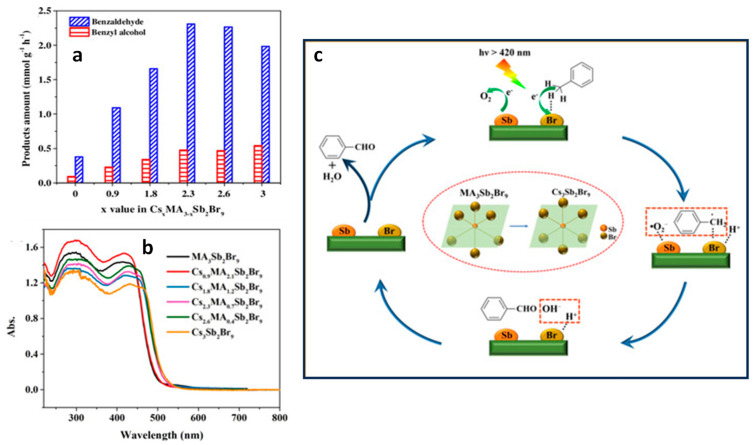
(**a**) Photocatalytic activity of toluene oxidation with Cs_x_MA_3−x_Sb_2_Br_9_ NP (x = 0–3) photocatalysts; (**b**) optical absorption spectra of Cs_x_MA_3−x_Sb_2_Br_9_ NPs (x = 0–3). Possible mechanism of A-site effect on C(sp^3^)–H bond activation; (**c**) photocatalytic process of toluene oxidation; middle part: octahedron distortion caused by A-site cations. Reproduced with permission. Copyright: 2020, John Wiley and Sons.

To study the influence of A-site cation in A_3_Sb_2_Br_9_ on the photocatalytic organic transformation, Zhang et al. used A_3_Sb_2_Br_9_ (A = MA, Cs) NPs for the visible light-driven photocatalysis of toluene oxidation via C–H bond activation [159]. Interestingly, in the Cs_x_MA_3−x_Sb_2_Br_9_ structure, Cs_3_Sb_2_Br_9_ NPs exhibited 5.3 times higher activity than MA_3_Sb_2_Br_9_ despite a slight narrowing of the bandgap (approximately 0.13 eV). The maximum photocatalytic activity was observed for x = 2.3 (Figure 39a). However, this significant enhancement in activity cannot be solely explained by the slight narrowing of the bandgap (Figure 39b). Instead, it is attributed to the raising of the VB, which reduces the oxidation ability of toluene.

XPS analysis was performed to reveal the mechanism of A-site on the photocatalytic activity. It was found that, with increasing Cs in Cs_x_MA_3−x_Sb_2_Br_9_, the binding energy of Sb 4d peaks shift to lower energy (0.25 to 0.31 eV), indicating that Sb became electron richer and, subsequently, Br became slightly electron deficient. The octahedron distortion values based on the c/a values suggests that the octahedron distortion increases with MA content in Cs_x_MA_3−x_Sb_2_Br_9_ (Figure 39c). Consequently, there was an increase in the electron cloud density of Br sites on the surface increases, which weakened the C–H activation process (Figure 39c). This octahedron distortion is caused by the Jahn–Teller effect via an A-site cation, which influences the crystal field splitting energy of [SbBr_6_] octahedra. In addition, Cs_3_Sb_2_Br_9_ NPs showed good stability for up to four cycles and a slight decrease in the photocatalytic activity from 2 to 1.62 mmol·h^−1^·g^−1^. The findings clearly indicated that the photocatalytic activity strongly correlated with the octahedral distortion of [SbBr_6_] affected by A-site cations.

### 13.12. Cs_3_Bi_2_Br_9_/SiO_2_ Heterostructures

**Figure 40:**
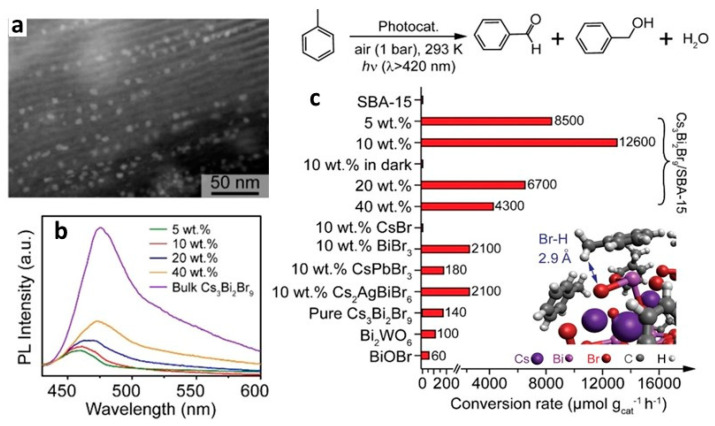
(**a**) HAADF-STEM images of 10 wt% loading; (**b**) PL spectra of supported and bulk Cs_3_Bi_2_Br_9_; (**c**) Toluene conversion rate over different samples. For all supported samples, SBA-15 was used as support and the effective catalyst mass was the weight of loaded halide perovskite phase. Inset. Electronic structure calculations. Focus on a typical Br-H geometry. Reproduced with permission. Copyright: 2020, John Wiley and Sons.

Dai et al. introduced a novel approach to enhance charge separation efficiency and active catalytic centres by integrating small Cs_3_Bi_2_Br_9_ nanoparticles (2–5 nm) into ordered mesoporous SBA-15 silica for photocatalytic C–H bond activation [160]. These nanoparticles are confined within the pore channels of SBA-15, as evidenced by the high-angle annular dark-field mode-scanning electron microscopy (HAADF-STEM) image (in Figure 40a), and they provide a significant number of catalytically active centres. The PL spectrum reveals a decrease in PL intensity for Cs_3_Bi_2_Br_9_-loaded SBA-15 compared to bulk Cs_3_Bi_2_Br_9_, indicating the suppression of radiative recombination of electron–hole pairs (Figure 40b). Notably, the compositions with 5 and 10 wt% Cs_3_Bi_2_Br_9_-loaded SBM-15 exhibited lower PL intensity, indicating an enhanced charge separation within these configurations. The Cs_3_Bi_2_Br_9_/SBA-15 composite was employed for the C–H bond activation of toluene in the presence of air and under visible light irradiation. Comparing different compositions, the 10 wt%-loaded SBA-15 demonstrated the highest conversion rate of 12,600 µmol g^−1^·h^−1^, with a high selectivity of 90%, as shown in the Figure 40c. Moreover, the extension of the photocatalytic study to other aliphatic and aromatic hydrocarbons results in an excellent performance. Notably, the highest conversion rate of 32,900 µmol g^−1^·h^−1^ was reported for the transformation of ethyl benzene to acetophenone.

DFT studies conducted to gain more insight into the interactions between the catalyst and toluene revealed that the interaction of benzene rings with Bi or Cs occurs through dispersion and charge transfer (π system to Bi σ* orbitals). Additionally, the hydrogen atoms of the -CH_3_ groups in toluene are located near the surface Br atoms of the catalyst, as shown in the inset of Figure 40c. Furthermore, the VBM of Cs_3_Bi_2_Br_9_ is mainly dominated by Br 4p orbitals, while Bi p-orbitals contribute to the CBM. As a result, the C–H bond activation induced by photogenerated holes on the bromine atoms results in efficient toluene oxidation.

### 13.13. DP NCs

**Figure 41:**
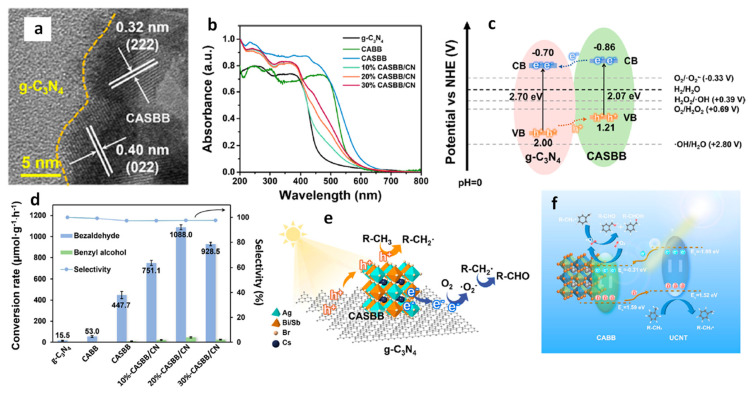
(**a**) HRTEM images of the 20% CASBB/CN composite; yellow dotted line represents the interface between g-C_3_N_4_ and Cs_2_AgBiBr_6_; (**b**) UV-vis DRS of g-C_3_N_4_, Cs_2_AgBiBr_6_, CASBB, and CASBB/CN composites; (**c**) band alignment of g- C_3_N_4_ and 20% CASBB/CN; (**d**) photocatalytic performance of toluene conversion and selectivity of benzaldehyde over the difference photocatalysts; (**e**) schematic of charge migration for the photocatalytic reactions in 20% CASBB/CN. Reproduced with permission. Copyright: 2023, American Chemical Society; (**f**) Possible mechanism for the photocatalytic oxidation of toluene by CABB-80@UCNT. Reproduced with permission. Copyright: 2023, Elsevier.

Ag incorporation into 2D-layered Cs_3_Bi_2_Br_9_ (CBB) perovskite led to the formation of 3D Cs_2_AgBiBr_6_ (CABB) double perovskite, thus eliminating the strong localisation of electron–hole pairs [161]. Consequently, CABB exhibited better properties than CBB, such as a low exciton binding energy, effective carrier mass, higher carrier mobility, long carrier lifetimes. In addition, the bandgap becomes narrow (from 2.7 eV of CBB to 2.1 eV of CABB) due to the participation of Ag 4d orbitals in the VB. To further narrow down the bandgap of CABB, Bi^3+^ was replaced with Tl^3+^, Sb^3+^, In^3+^, and Fe^3+^ in Cs_2_AgBiBr_6_ [162,163,164]. However, Sb-substitution is more suitable for photocatalytic applications; the introduction of 37.5% Sb in CABB narrows the bandgap up to 1.86 eV owing to the rise in VB by participating Sb 5s orbitals above the Bi 6s orbitals [163]. Moreover, introducing Sb^3+^ in Bi-based perovskites prolongs the charge carrier lifetimes. To have strong light absorption, along with excellent carrier separation, Li et al. used Sb-substituted Cs_2_AgBiBr_6_/g-C_3_N_4_ (CASBB/CN) composite for photocatalytic C–H bond activation [165]. The CASBB/CN composite was made by mechanical grinding powders of ASBB and using different ratios of g-C_3_N_4_. Figure 41a shows the HRTEM image of the 20% CASBB/CN composite, where a clear boundary can be seen between crystalline CASBB perovskite and amorphous g-C_3_N_4_. The origin of the chemical connectivity has been attributed to a strong interaction between NH_x_ groups of g-C_3_N_4_ and bromide ions.

Figure 41b shows the absorption spectrum, where the g-C_3_N_4_ shows an absorption edge of 450 nm (2.7 eV). Notably, the absorption of visible light enhanced by increasing the loading amount of Sb in the CASBB/CN composite was compared with CABB. Furthermore, the band alignment of the CASBB/CN composite shows a type-II heterojunction (in Figure 41c), which facilitates excellent charge separation. The photocatalytic activity of toluene oxidation is shown in Figure 41d. Among all the compositions, CASBB/CN composites showed the highest activity of toluene conversion, followed by CASBB, CABB, and g-C_3_N_4_ with high selectivity 96%. The 20% CASBB/CN composite showed the highest activity of benzaldehyde, with a production rate of 1088 µmol·g^−1^·h^−1^. The high activity is due to extended wavelengths of light absorption and the improved charge separation. Figure 41e shows the schematic mechanism of benzaldehyde formation.

In a similar strategy, Song et al. also demonstrated the formation of heterostructures of CABB with an ultra-thin carbon nitride (UCNT) sheet for photocatalytic oxidation of toluene [166]. The in-situ growth of Cs_2_AgBiBr_6_ on a UCNT sheet led to strong chemical interaction between perovskite and UCNT. Moreover, the type-II band alignment of CABB/UCNT facilitates the efficient charge separation. In the case of the CABB/UCNT composite, the photogenerated electrons transfer to the CB of CABB, whereas photogenerated holes transfer to the VB of the UCNT, as shown in Figure 41f; this behaviour is opposite to the transfer of photogenerated carriers in the CASBB/CN composite. As shown in the scheme, the reduction of O_2_ takes place on the surface of perovskite, and toluene becomes oxidized on the surface of the UCNT. Consequently, the reaction between the benzyl radical and superoxide radical leads to the formation of benzaldehyde and benzyl alcohol. Thus, the best conversion rate of toluene, 2630 µmol·g^−1^·h^−1^, has been achieved using the CABB-80/UCNT composite.

### 13.14. A_4_M^II^M_2_^III^X_12_ LDP NCs

Solis-Ibarra and co-workers conducted a study revealing that Cs_4_CuSb_2_Cl_12_ LDP exhibits promising optical properties for solar and photocatalytic applications. Notably, Cs_4_CuSb_2_Cl_12_ LDPs displayed a significantly higher electrical conductivity, approximately one order of magnitude greater than that of MAPbI_3_ perovskite. This suggests that these lead-free materials hold great potential for future photocatalytic applications.

**Figure 42:**
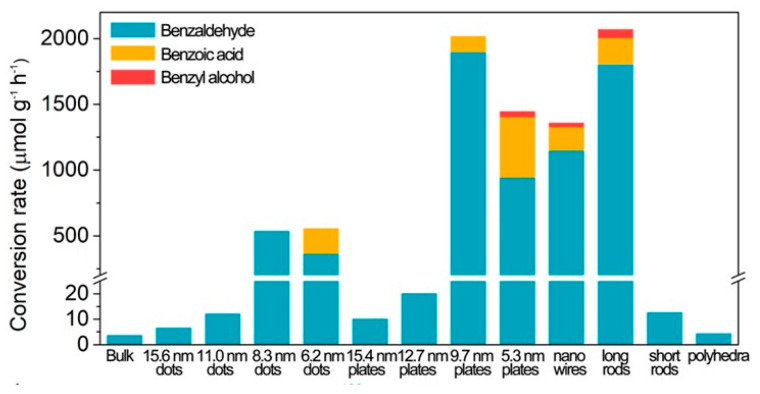
Toluene conversion rate over different samples of Cs_4_ZnSb_2_Cl_12_. Reproduced with permission. Copyright: 2023, American Chemical Society.

In a more recent study, Caruso and co-workers explored the tunability of size and morphology in Cs_4_ZnSb_2_Cl_12_ LDP NCs and their impact on toluene photooxidation reactions. Figure 42 illustrates the conversion rate of toluene into benzaldehyde, with benzyl alcohol and benzoic acid as the byproducts, using various Cs_4_CuSb_2_Cl_12_ LDP NC morphologies. Notably, 9.7 nm nanoplates exhibited a remarkable performance compared with other photocatalysts given in the Table 2, achieving a high benzaldehyde formation rate of up to 1893 µmol·g^−1^·h^−1^ with an impressive selectivity of 95%. This achievement is particularly remarkable given that the NCs were capped with long chain ligands, specifically oleylamine/oleic acid ligands. Furthermore, the concept of creating heterostructures by combining A_4_M^II^M_2_^III^X_12_ LDPs with 2D materials holds the promise of significantly enhancing photoactivity beyond what A_4_M^II^M_2_^III^X_12_-type NCs can achieve on their own. This opens exciting possibilities for advancing photocatalytic applications.

## 14. Oxidation of 5-Hydroxymethylfurfural to 2,5-Diformylfuran

Biomass has garnered significant attention as a vital renewable carbon feedstock, serving as a promising alternative to petroleum resources to produce sustainable fuels and high-value fine chemicals. Within the realm of biomass-derived compounds, 5-hydroxymethylfurfural (HMF) possesses the remarkable ability to undergo selective transformations, leading to the creation of a variety of multifunctional furanic products. One of the most noteworthy outcomes is 2,5-diformylfuran (DFF), which has found widespread application in both the chemical and pharmaceutical industries.

The traditional method of oxidation for this transformation relies on copious stoichiometric oxidants, resulting in substantial waste generation and environmental pollution. However, for economic and sustainable development considerations, there is a strong preference for a greener approach. The use of green photocatalytic oxidation, employing molecular oxygen to convert HMF into DFF, has emerged as an attractive synthetic pathway.

Lv and co-workers employed bulk MAPbBr_3_ perovskite-based photocatalysts for the highly selective oxidation of HMF to DFF [170].

**Figure 43:**
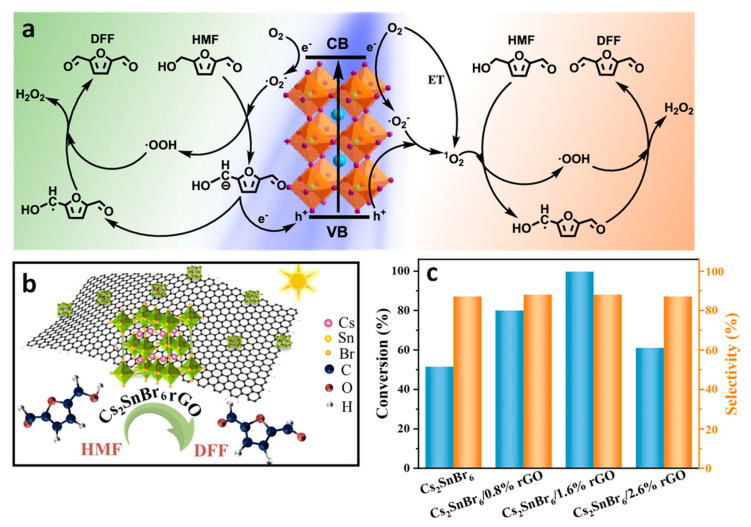
(**a**) Proposed catalytic mechanism on the selective oxidation of HMF utilizing MAPbBr_3_ Photocatalyst and Atmospheric O_2_; (**b**) schematic representation for the photocatalytic selective oxidation of HMF based on the Cs_2_SnBr_6_/rGO catalytic system. Reproduced with permission. Copyright: 2020, American Chemical Society; (**c**) photocatalytic selective oxidation of HMF performance using different compositions of Cs_2_SnBr_6_/rGO. Reproduced with permission. Copyright: 2023, John Wiley and Sons.

This photocatalytic process was conducted in an acetonitrile solvent using an O_2_ atmosphere and 450 nm LED light irradiation. This resulted in the complete conversion of HMF into DFF, with an impressive selectivity of over 90% and a yield exceeding 96%. In Figure 43a, the mechanism behind the formation of DFF from HMF is depicted, wherein photogenerated electrons are transferred to O_2_, forming ·O_2_^−^ and singlet oxygen (^1^O_2_). These intermediates then react with HMF to yield the final product, DFF, along with the byproduct, H_2_O_2_. It is worth noting that this reaction proved unsuccessful when using MAPbCl_3_ and MAPbI_3_ photocatalysts. In the case of MAPbCl_3_, the self-decomposition of HMF occurred under 365 nm LED light illumination, rendering the reaction unfeasible. Similarly, MAPbI_3_ decomposed under 450 nm LED illumination.

Wang and co-workers carried out a similar reaction by employing a Schottky heterojunction consisting of lead-free Cs_2_SnBr_6_ (bandgap 2.86 eV) and reduced graphene oxide (rGO), as shown in Figure 43b [171], using a 300 W Xe lamp with a 400 nm cut-off filter. The outcome was a selective conversion of HMF to DFF (>99.5%), while a selectivity of 88% for DFF was obtained when using Cs_2_SnBr_6_/rGO heterostructures (Figure 43c). Additionally, the Cs_2_SnBr_6_/rGO combination produced an overoxidation product of DFF, namely 5-formyl-2-furancarboxylic acid (FFCA). It is important to note that Cs_2_SnBr_6_ differs from typical perovskite AM^II^X_3_ materials, forming scattered octahedra [SnI_6_]^2−^ in a vacancy-ordered double perovskite structure. While this structure has inherent limitations in charge transport, the creation of a Schottky heterojunction significantly enhances the charge extraction efficiency, leading to a notable improvement in the performance of Cs_2_SnBr_6_/rGO heterojunctions compared to pristine Cs_2_SnBr_6_.

## 15. Csp^3^-Csp^3^ Coupling Reaction Using Lead-Free Cs_3_Sb_2_Br_9_ NCs

**Figure 44:**
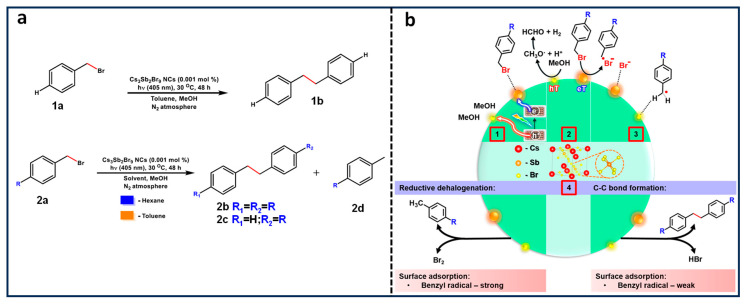
(**a**) C-C coupling reactions of benzyl bromide (1a), para-substituted benzyl bromide (2a); (**b**) proposed mechanism of the photocatalytic benzyl bromide photoreduction; (1). photoinduced electron and hole generation, charge diffusion, and substrate–surface approximation; (2). electron transfer to the substrate to generate the radical anion of the benzyl bromide and hole transfer to the MeOH (formaldehyde and H_2_ formation); (3) adsorption of the Br anion and benzyl bromide radical to the NC surface; the strength of the binding of the benzyl radical to the NC surface would depend on the electronegativity of the substituent at the p-position; and (4) competitive formation of the reductive dehalogenation product vs. the C–C coupling product.

Perez-Prieto et al. reported a significant enhancement in the performance of the C–C coupling reaction when using a Cs_3_Sb_2_Br_9_ NCs photocatalyst for benzyl bromide and its substituted aryl bromides [172]. The TON reached approximately 105,000. This high TON was attributed to the favourable alignment of CBM of the Cs_3_Sb_2_Br_9_ NCs relative to the unoccupied molecular orbital (LUMO) of benzyl bromide, which eased the photogenerated electron transfer. The C–C coupling reaction was carried out under a N_2_ atmosphere, using methanol as a hole scavenger and toluene as the solvent and a 405 nm light excitation. The choice of solvent was found to be crucial in the photoreduction of 1a (in Figure 44a). In the presence of toluene, 1a forms 1b (in Figure 44a) with a yield of 77%, whereas this yield decreased for other solvents, such as hexane (~34), chlorobenzene (~24), and ethyl acetate (~14).

When the same reaction was performed for *p*-substituted benzyl bromides in the presence of toluene, three types of products were formed: homocoupling product 1b from substrate 1a, 2b from substrate 2a (in Figure 44a), heterocoupling product 2c from substrate 2a and toluene solvent, and the dehalogenated product 2d. On the other hand, when the same reaction was performed under a hexane solvent, only b and d products were formed. The number and yield of the product can be modulated by changing the solvent and electron-donating/electron-withdrawing nature of the substituent. The following trend for the C–C homocoupling has been observed: OCH_3_ < H < tBu < Br ≈ Cl in hexane solvent. An electron-donating substituent favours the dehalogenated product formation, while p-nitrobenzyl bromide did not undergo photoreduction in any of the solvents. These results suggest the different interaction of the substrate with the NC surface.

Furthermore, investigations into lead-free perovskite NCs have extended to their application in Csp^3^-Csp^3^ coupling reactions, such as α-alkylation of aldehydes. In a study conducted by Zhao and collaborators, oleic acid-capped Cs_2_AgSbCl_6_ double perovskite NCs, with a bandgap of 2.65 eV, were employed for the C–C coupling reaction involving 2-bromoacetophenone and n-octanal as a model reaction, mirroring the depicted reaction in Figure 7a [173]. The band alignment of Cs_2_AgSbCl_6_ NCs was found to resemble that of octylamine-capped CsPbBr_3_ NCs utilized for α-alkylation, as illustrated in Figure 7a. The authors achieved important reaction yields with a TON of ~14,800, particularly for substrates featuring both electron-donating and electron-withdrawing substituents on the phenyl ring of 2-bromoacetophenone. The meticulous washing of the NCs was imperative to facilitate efficient charge transfer and attain high product yields. The reaction mechanism involves a radical-mediated pathway akin to path III, illustrated in Figure 7b.

## 16. Conclusions

In this comprehensive review, we have introduced the recent advances in the field of metal halide perovskite-based organic molecular transformations, which have emerged as promising candidates for photocatalysis applications owing to their favourable optical and electronic properties as well as redox characteristics. The ease of preparation and recyclability further positions perovskite-based heterogeneous photocatalysis as superior to metal oxide/sulfide-based heterogeneous photocatalysts and molecular-based homogeneous photocatalysis.

Motivated by the success of Pb-based perovskite materials in solar cell applications, researchers have shifted their focus towards investigating organic molecular transformations. Pb-based perovskites have demonstrated remarkable efficacy in visible light-driven chemical reactions via charge and energy transfer processes, encompassing various organic reactions, such as C–H bond activation, C–X (X = C, N, O) bond formation, cycloaddition reactions, asymmetric synthesis, and chiral molecule synthesis. Leveraging the defect-tolerant nature of Pb-based PNCs with high PL QY, energy transfer-based photocatalytic reactions have yielded moderate-to-high reaction yields. It is noteworthy that smaller NCs exhibit superior photocatalytic activity, albeit with perovskite degradation becoming apparent after a few catalytic cycles.

In response to the instability observed in 3D Pb-based perovskites, researchers have explored low-dimensional and layered perovskites by incorporating larger-sized organic ligands. This approach not only enhances stability, particularly in polar solvents, such as water, but also yields promising results in decarboxylation and dehydrogenation reactions. Furthermore, efforts to address Pb-toxicity concerns have led to the proposal of Pb-free perovskite materials, including lower-dimensional structures, such as DPs and layered DPs. Surprisingly, lower-dimensional perovskite materials have exhibited superior photocatalytic activity compared to Pb-based perovskites, showcasing promise in toluene oxidation and C–C coupling reactions. Lead-free counterparts have demonstrated the ability to facilitate novel chemical reactions, e.g., by the ring-opening reactions of epoxides via Cs_3_Bi_2_Br_9_ due to their Lewis acidic character, along with high selectivity and turnover numbers for benzyl bromide coupling reactions. However, challenges persist due to the poor defect-related luminescence of lead-free counterparts, limiting their application in energy transfer-based photocatalytic reactions.

To optimize visible light absorption, electron–hole separation, and band alignment for accessing redox energy levels, researchers have successfully developed heterostructures, including type-II, Z-scheme, and Schottky heterojunctions. These heterostructures not only safeguard perovskite NCs but also enhance photocatalytic activity and stability. Nevertheless, challenges arise in directly comparing photocatalytic activity due to variations in material bandgap, morphology, and experimental conditions. Thorough characterization of heterostructures is crucial, with the potential existence of p-n junction characteristics in reported type-II heterojunctions requiring confirmation through comprehensive Fermi level characterization. Alternatively, tuning type-II heterojunctions into p-n heterojunctions through doping and changing the stoichiometry of perovskite [174] and other semiconductors (g-C_3_N_4_, Mxene) [175,176] could enhance the spatial separation of electrons and holes, thereby promoting efficient photocatalysis.

Furthermore, the engineering of materials and optimization of reaction conditions are vital for surpassing the current state-of-the-art methods in photocatalytic organic chemical transformations. Layered perovskites, exemplified by Cs_4_ZnSb_2_Cl_12_, exhibit promising bandgaps, excellent transport properties, and quantum confinement effects, surpassing the performance of both Pb-based and other Pb-free perovskites. The creation of heterostructures holds immense potential for expanding the scope of photochemical reactions [177].

Despite achieving high performance, the recycling stability of the state-of-the-art photocatalysts is currently limited to three to five cycles. To address this challenge, a promising avenue involves exploring core/shell heterostructure nanocrystals (NCs), with a specific focus on enhancing stability through optimized shell thickness and encapsulation strategies. Examples include CsPbBr_3_@CdS [178], CsPbBr_3_@Cs_4_PbBr_6_, CsPbBr_3_@CsPb_2_Br_5_ [179], CsPbBr_3_@Rb_4_PbBr_6_ [180], CsPbBr_3_@PbBr_2_ [181]. Additionally, encapsulating the active material with inorganic oxides such as SiO_2_ [182], TiO_2_ [183], AlO_x_ [184], ZrO_2_ [185], silsesquioxane [186], and ZrN_x_ [187] as well as hydrophobic polymer matrices, e.g., polystyrene [188], poly(maleic anhydride-alt-1-octadecene) [189], and PMMA [190].

While, porous metal–organic frameworks (MOFs) and covalent organic frameworks (COFs) are currently the primary choices for encapsulation [191,192], there is a need for the development of novel, easily synthesizable encapsulation materials to usher in a new era of stability. Semiconducting polymers and monodispersed capping agents, boasting suitable porous structures, hydrophobicity, chemical interactions, and band energies, present themselves as potential game-changers in the stabilization paradigm [193]. Future investigations should pivot towards combined approaches that simultaneously elevate both activity and stability.

Incorporating surface functional groups and capping layers onto quantum dots (QDs) and NCs allows for harnessing the high activity of small-sized halide perovskite photocatalysts without compromising stability [194]. Moreover, the strategic construction of heterojunctions, specifically type-II and Z-scheme, at the nano and sub-nanoscale within porous semiconductor networks holds significant promise for revitalizing perovskite-based photocatalysis, ensuring heightened performance on both the activity and stability fronts [179,195,196,197,198].

Recent reports on perovskite solar cells suggest that orienting perovskite precursors, such as PbI_2_, in certain directions on the film can modulate the final perovskite crystal orientations (topochemical assembly) and minimize the defect state density of the perovskite crystal lattice, ultimately realizing high solar cell efficiencies with long stability [199]. This strategy can be applied in solar cell-type-based photocatalysts. Similar strategies can be extended to colloidal heterostructure NCs, such as using PbS QDs as seeds and allowing for the crystal growth of perovskite on the surface of PbS, enabling the formation of heterojunctions with smaller lattice mismatch and showing more stable perovskite crystal structures, especially in polar solvent mediums [195,200,201]. This methodology allows for stable iodide-based perovskites with narrow bandgaps for solar cell and photocatalytic applications [202].

Additionally, the investigation of lead-free perovskite materials, such as copper halides and chalcogenides, with favorable opto-electronic properties stands as a promising direction for advancing the field of photocatalysis [203,204].

In conclusion, this review not only highlights current achievements but also points towards exciting avenues for future research in the dynamic field of perovskite-based photocatalysis. The continuous exploration of materials, optimization strategies, ligand chemistry, and innovative heterostructure designs promises to propel the field towards more sustainable and efficient photocatalytic organic transformations.

## Figures and Tables

**Table 2:** State-of-the-art of the photocatalytic toluene oxidation of lead and lead-free perovskites. C% and S% represent conversion and selectivity, respectively.

Photocatalyst	Light Source	Band Gap	Synthesis Method	C %	S %	Activity (µmol·g^−1^·h^−1^)	Ref
FAPbBr_3_	150 W Xe lamp, AM 1.5 G simulated light irradiation	2.2	LARP	0.075	81	320	[97]
CsPbBr_3_	300 W Xe lamp with 420 nm filter	2.3	LARP	-	-	710	[124]
CdS	300 W Xe lamp, visible light (λ > 420 nm)	2.25	room temp.	33	100	240	[167]
TiO**_2_**	Six 6 W UV lamps (310 nm)	~3.7	Hydrothermal	21%	90	94	[168]
Flower-like Bi_2_WO_6_	300 W Xe lamp, visible light (λ > 420 nm)	2.96	Hydrothermal	1.5%	~85	464	[169]
CsPbBr_3_**_−_**_x_Cl_x_/TiO_2_	300 W Xe lamp with 420 nm filter	2.62	LARP & anion exchange	-	-	1874	[124]
5% NiO_x_/FAPbBr_3_/TiO_2_	150 W Xe lamp, AM 1.5 G	-	LARP	0.85%	86	3800	[97]
Cs_3_Bi_2_Br_9_ NCs	300 W Xe lamp	2.75	LARP	-	100	850	[141]
Cs_3_Bi_2_Br_9_/Mxene-7.5	300 W Xe lamp	~2.5	In-situ growth	-	-	4011	[141]
Cs_3_Bi_2_Br_9_/SBA-15(10%)	300 W Xe lamp with 420 nm filter	2.74	wetness impregnation	-	90	12,600	[160]
Cs_3_Sb_2_Br_9_	150 W Xe lamp, AM 1.5 G with 420 nm filter	2.3	Anti-solvent precipitation	-	-	1195	[148]
g-C_3_N_4_	150 W Xe lamp, AM 1.5 G with 420 nm filter	2.67	Pyrolysis	-	-	298.3	[148]
Cs_3_Sb_2_Br_9_/g-C_3_N_4_	150 W Xe lamp, AM 1.5 G with 420 nm filter	2.3	Anti-solvent precipitation	-	-	8347	[148]
Cs_2_AgBiBr_6_	500 W Xe lamp, with 420 nm filter	2.21	hydrothermal	-	10	53	[165]
Cs_2_AgSb_x_Bi_1−x_Br_6_	500 W Xe lamp, with 420 nm filter	2.07	hydrothermal	-	40	448	[165]
Cs_2_AgSb_x_Bi_1−x_Br_6/_CN (20%)	500 W Xe lamp, with 420 nm filter	-	Mechanical grinding	-	96	1088	[165]
Cs_4_ZnSb_2_Cl_12_ nanoplates	500 W Xe lamp, with 420 nm filter	2.36	Hot injection	-	95	1893	[29]
